# Coumarins by Direct Annulation: β‐Borylacrylates as Ambiphilic C_3_‐Synthons

**DOI:** 10.1002/anie.202012099

**Published:** 2020-11-09

**Authors:** Max Wienhold, John J. Molloy, Constantin G. Daniliuc, Ryan Gilmour

**Affiliations:** ^1^ Organisch Chemisches Intitut Westfälische Wilhelms-Universität Münster Corrensstraße 36 48149 Münster Germany

**Keywords:** annulation, boron, catalysis, heterocycles, isomerisation

## Abstract

Modular β‐borylacrylates have been validated as programmable, ambiphilic C_3_‐synthons in the cascade annulation of 2‐halo‐phenol derivatives to generate structurally and electronically diverse coumarins. Key to this [3+3] disconnection is the BPin unit which serves a dual purpose as both a traceless linker for C(sp^2^)–C(sp^2^) coupling, and as a chromophore extension to enable inversion of the alkene geometry via selective energy transfer catalysis. Mild isomerisation is a pre‐condition to access 3‐substituted coumarins and provides a handle for divergence. The method is showcased in the synthesis of representative natural products that contain this venerable chemotype. Facile entry into π‐expanded estrone derivatives modified at the A‐ring is disclosed to demonstrate the potential of the method in bioassay development or in drug repurposing.

## Graphical abstract

Modular β‐borylacrylates are validated as programmable, ambiphilic C_3_‐synthons for the annulation of 2‐halo‐phenol derivatives to generate diverse coumarins. Key to this [3+3] disconnection is the BPin unit which serves a dual purpose as both a traceless linker for C(sp^2^)–C(sp^2^) coupling, and as a chromophore extension to enable *E* → *Z* alkene isomerisation. Inverting alkene geometry is a pre‐condition to access 3‐susbtituted derivatives. This approach capitalises upon the abundance of 2‐halophenols in biology and may facilitate natural product/drug re‐purposing and the development of imaging tools.

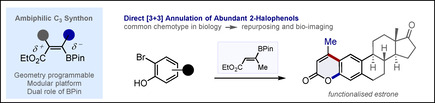

Innovative advances in annulation chemistry have been a defining feature in the evolution of organic synthesis: This reflects the importance of heterocycles in target synthesis, and in the design of functional small molecules in a broader sense.^[1]^ As exemplified by the venerable Huisgen cycloaddition,[^2^, ^3^] operationally simple “*click*” processes to construct unnatural heterocyclic scaffolds have been intensively pursued and enjoy widespread application in chemical biology.^[4]^ Predicated on high efficiency, tolerance and atom economy,^[5]^ these enduring processes serve as valuable guidelines for the conception of annulation routes to naturally‐occurring heterocyclic scaffolds. In particular, the revered history of the coumarin nucleus as a drug module and natural product core, together with its importance in functional materials, constitute persuasive arguments to devise more effective synthesis routes based on direct annulation.^[6]^ The long‐standing importance of this bicyclic framework is evident from the early works of Perkin^[7]^ and Pechmann,^[8]^ which have since been complemented by an array of transition metal,^[9]^ photochemical^[10]^ and modified Knoevenagel condensation approaches.^[11]^ To further strengthen this diverse synthesis arsenal with a direct coupling, a [3+3] approach was envisaged that would unify halogenated phenols (**I**) with a simple ambiphilic C_3_ fragment (**II**) via two successive bond‐forming events (Figure 1). β‐Borylacrylates (**II**)^[12]^ were conceived as attractive coupling partners for a Suzuki–Miyaura/substitution cascade due to their ease of preparation and structural tenacity.^[13]^ Moreover, the BPin unit in this modular acrylate platform serves as both a traceless linker for C(sp^2^)–C(sp^2^) coupling, and as a chromophore antenna to enable inversion of the alkene geometry^[14]^ via selective energy transfer catalysis.^[15]^ In the absence of base‐mediated isomerisation,^[16]^ regulating alkene geometry is necessary to facilitate lactonisation to access 3‐substituted coumarins.

**Figure 1:**
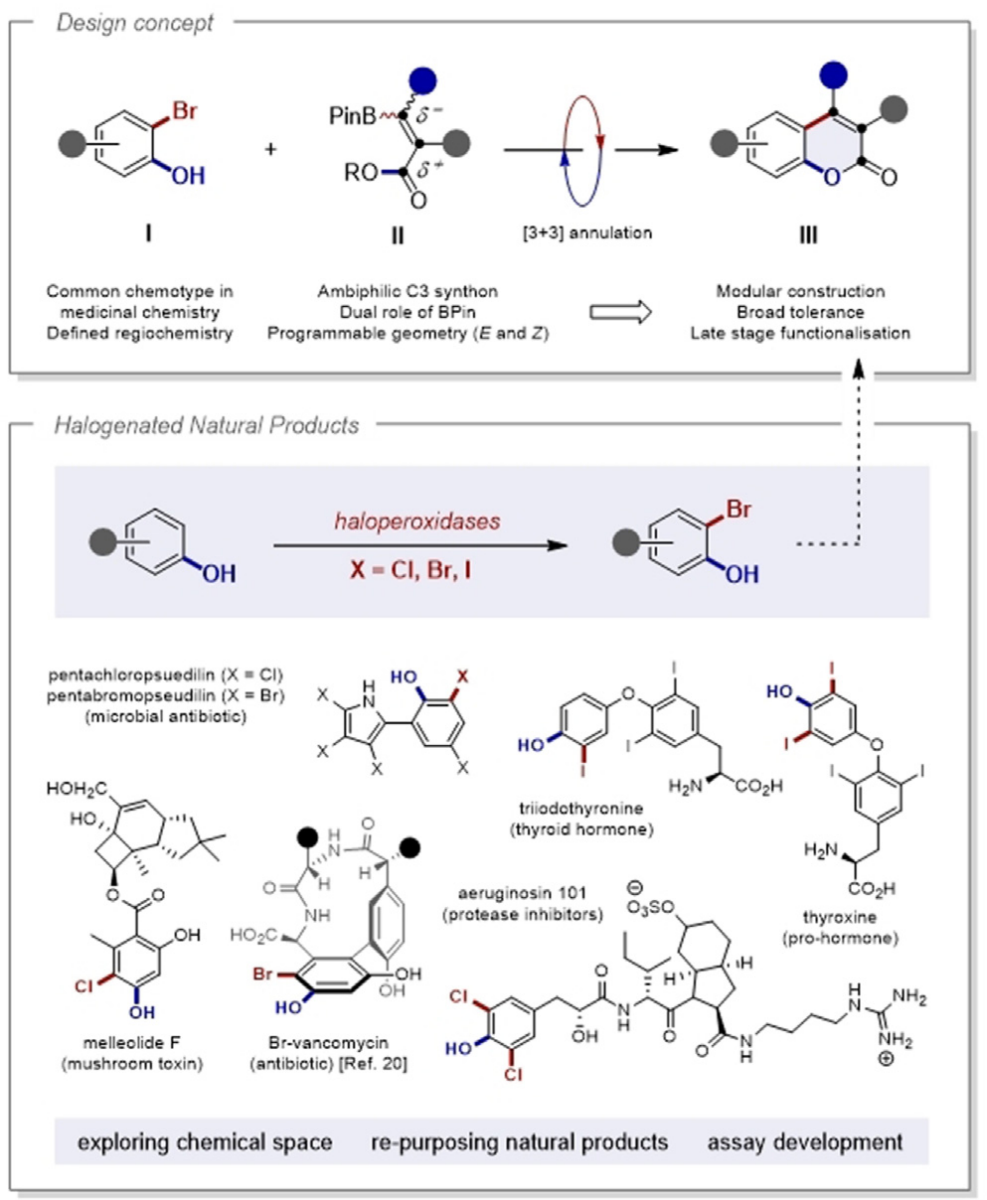
Top: Conceptual framework for a direct 2‐halo‐phenol to coumarin annulation using β‐borylacrylates. Bottom: Halogenated natural products.^[17]^

This disconnection capitalises upon abundance of 2‐halophenols in biology (Figure 1, bottom),^[17]^ their regioselective halogenation chemistries[^17^, ^18^] and the importance of the coumarin scaffold (**III**) in both drug discovery and molecular imaging.^[19]^ Given the notable methodological advances in selective halogenation,^[20]^ it was envisaged that this [3+3] annulation may facilitate natural product/drug re‐purposing.^[21]^

To validate the conceptual framework outlined in Figure 1, the coupling of 2‐bromophenol and β‐borylacrylate ***Z*** **‐2** (1.0 equiv.) was explored (Table 1). Gratifyingly, exposing the coupling partners to Pd(OAc)_2_ and SPhos in 1,4‐dioxane at 80 °C generated the desired coumarin **3** in 30 % yield after 16 h (entry 1). Switching solvent to *N*,*N*‐dimethylformamide led to a modest improvement in yield (41 %, entry 2), whereas DMSO was detrimental (32 %, entry 3). Utilising 2.0 equiv. of β‐borylacrylate ***Z*** **‐2** in DMF led to an increased yield of 53 % (entry 4) and so these parameters were fixed for the reminder of the optimisation. Variation in the Pd source (entries 5 and 6) and base (entries 7 and 8) was then explored, but these alterations did not confer a notable advantage.

**Table 1:** Reaction optimisation in the formation of coumarin **3**. 
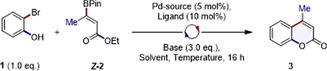

Entry	Catalyst	Ligand	Base	Solvent	H_2_O (equiv)	*T* [°C]	Yield [%]^[a]^
1^[b]^	Pd(OAc)_2_	SPhos	K_3_PO_4_	1,4‐dioxane	5.0	80	30
2^[b]^	Pd(OAc)_2_	SPhos	K_3_PO_4_	DMF	5.0	80	41
3^[b]^	Pd(OAc)_2_	SPhos	K_3_PO_4_	DMSO	5.0	80	32
4	Pd(OAc)_2_	SPhos	K_3_PO_4_	DMF	5.0	80	53
5	Pd(PPh_3_)_4_	–	K_3_PO_4_	DMF	5.0	80	37
6	Pd(dppf)Cl_2_^[c]^	–	K_3_PO_4_	DMF	5.0	80	45
7	Pd(OAc)_2_	SPhos	K_2_CO_3_	DMF	5.0	80	50
8	Pd(OAc)_2_	SPhos	CsCO_3_	DMF	5.0	80	31
9	Pd(OAc)_2_	SPhos	K_3_PO_4_	DMF	5.0	50	41
10	Pd(OAc)_2_	SPhos	K_3_PO_4_	DMF	5.0	100	39
11	Pd(OAc)_2_	SPhos	K_3_PO_4_	DMF	/	80	82(76)^[d]^

[a] Yields determined by NMR using 1,3,5‐trimethoxybenzene as internal standard. [b] 1.0 equiv. of ***Z*** **‐2** was used. [c] DCM complex. [d] Numbers in parenthesis are isolated yields.

Altering the temperature had a negative impact on yield (entries 9 and 10), but exclusion of water proved to be beneficial (82 %, entry 11). This observation, together with the yield enhancement when using 2.0 equiv., suggests that ***Z*** **‐2** displays limited stability under basic aqueous conditions. Given the involvement of an oxopalladium complex in the Suzuki–Miyaura mechanism,^[22]^ it is pertinent to note that the DMF used is not fully anhydrous and thus water is likely present in the reaction mixture.

Having identified optimised reaction conditions for the catalytic annulation, the scope and limitations were investigated using a range of electronically and structurally modified 2‐bromophenols (Figure 2). Systematically increasing the steric demand of the β‐substituent of the β‐borylacrylate from *n*‐propyl (**4**) to cyclopropyl (**5**) and methylcyclohexyl (**6**) was well tolerated (69 %, 67 % and 75 % yield, respectively). Incorporation of an aryl group furnished **7** in 73 % yield. The addition of an α‐substituent was also well tolerated enabling the formation of 3,4‐dimethylcoumarin (**8**) in 83 %. Given the importance of fluorinated aryl systems in drug discovery,^[23]^ the influence of bioisosteric H to F substitution on the phenol coupling partner was examined. This led to smooth formation of **9** in 76 % yield. Free amines were also compatible with the general conditions (**10**, 71 %), as were anisole derivatives enabling formation of **11** (63 %). Expected chemoselective C(sp^2^)–C(sp^2^) coupling was confirmed by formation of the chlorinated derivative **12** (63 %). To demonstrate the utility of the method in accessing more sterically congested, 2‐bromo‐6‐methylphenol and 1‐bromo‐2‐naphtol were exposed to the standard annulation conditions enabling formation of **13** (85 %) and **14** (80 %), respectively. The latter system demonstrates the suitability of this approach in accessing the naphthopyran‐2‐one class of photochromic dyes.^[24]^ Furthermore, the transformation enables formation of π‐expanded systems such as the biaryl derivative **15** in 68 %.^[6b]^

**Figure 2:**
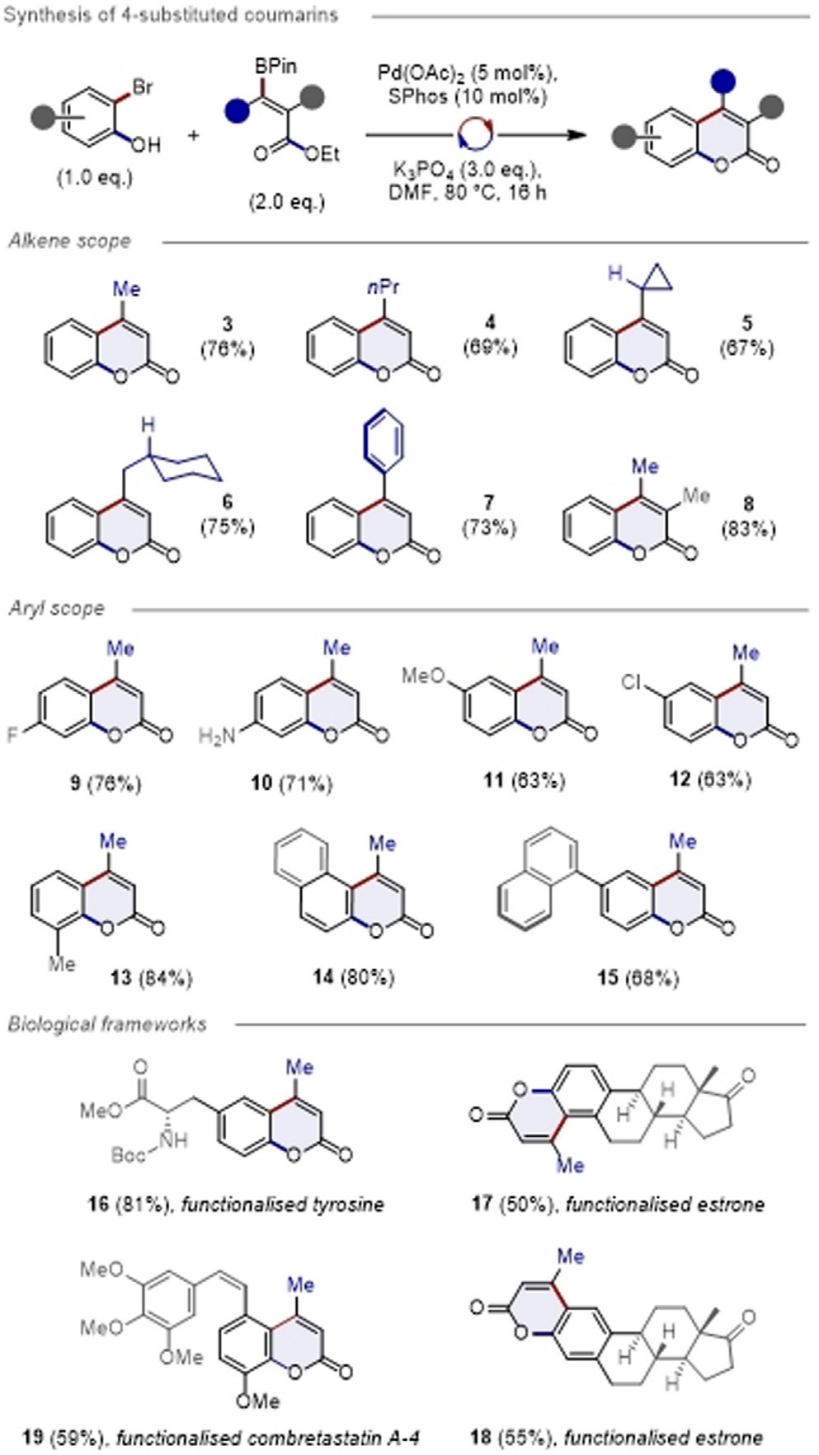
Scope of the annulation and application in natural product synthesis and derivatisation.

To demonstrate the synthetic utility of β‐borylacrylates in derivatising biological frameworks, commercially available 3‐bromotyrosine was processed to **16** in 81 %. Moreover, the terpene derivatives 2‐ and 4‐bromoestrone could be smoothly transformed into **17** and **18** (50 % and 55 %, respectively), enabling entry into novel π‐extended analogs of the parent hormone. A brominated derivative of the anti‐tumour agent combretastatin A‐4 underwent smooth annulation, enabling formation of the coumarin derivative **19** in 59 % yield. It is pertinent to note that combretastatin A‐4‐phosphate is currently in clinical development for ovarian cancer.^[25]^

The structure of compound **18** was unequivocally established by single‐crystal X‐ray analysis (CCDC number 2022960, Figure 3, top). This compound crystallised in the chiral space group *P*2_1_2_1_2_1_ (Flack parameter was refined to 0.00(6)). In the packing diagram the formation of linear chains along the *a*‐axis involving π**⋅⋅⋅**π interactions (*C*g1**⋅⋅⋅** *C*g1 3.286 Å) supported by additional C−H**⋅⋅⋅**O interactions (C8–H8B**⋅⋅⋅**O2 3.418(2) Å; 157.9° and C22–H22B**⋅⋅⋅**O2 3.434(2) Å; 159.6°) were observed (Figure 3, bottom). The prominence of π**⋅⋅⋅**π interactions between the aromatic units of estrone derivative **18** is presented in Figure 2 d in the Supporting Information. The 3D network of estrone derivative **18** is compact, Moreover, the influence of the coumarin in generating linear chains due to additional C−H**⋅⋅⋅**O interactions is evident from the extended packing analysis (Figure 3, bottom).

**Figure 3:**
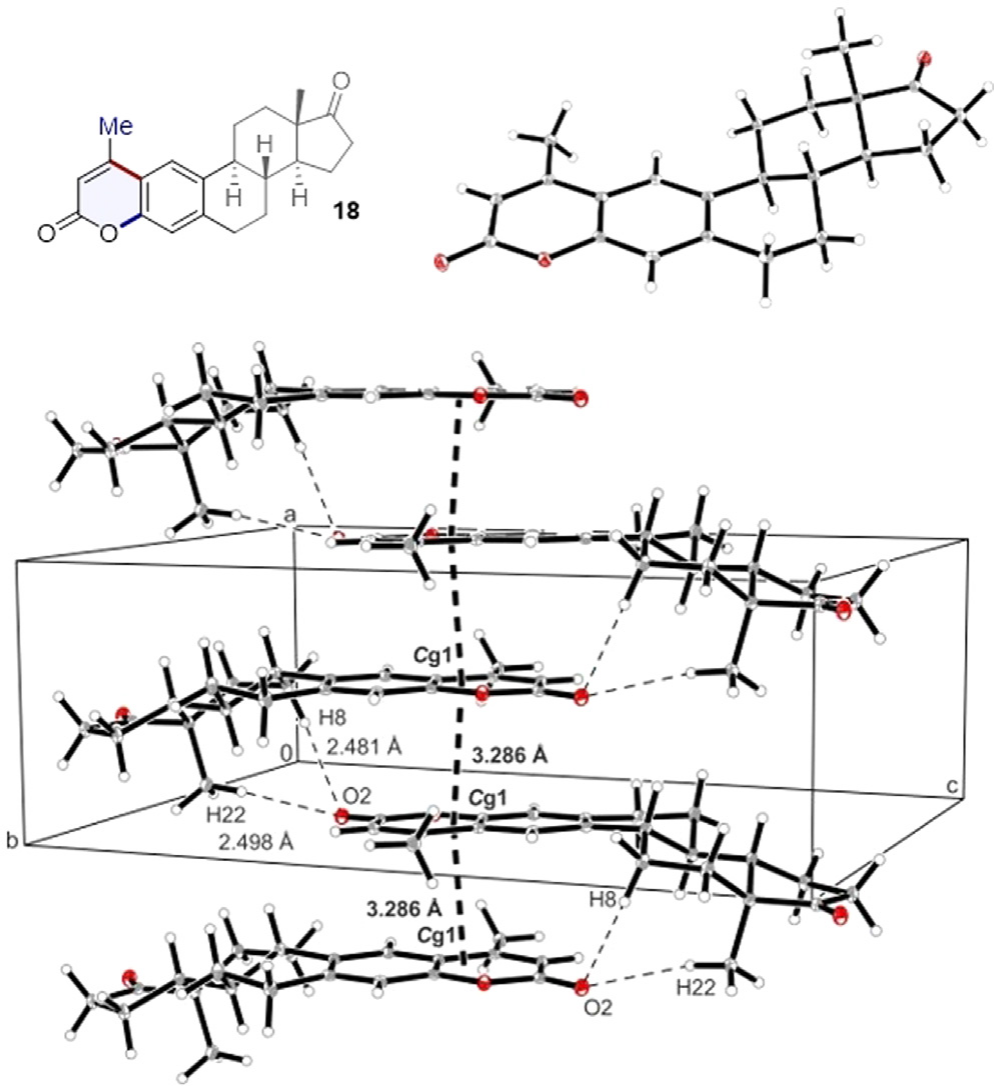
Top: The X‐ray crystal structure of estrone derivative **18** (Deposition Number 2022960 contains the supplementary crystallographic data for this paper. These data are provided free of charge by the joint Cambridge Crystallographic Data Centre and Fachinformationszentrum Karlsruhe Access Structures service www.ccdc.cam.ac.uk/structures.). Thermal ellipsoids are shown at 30 % probability. Bottom: Excerpt of the packing diagram of compound **18** presenting the formation of a linear chain along *a*‐axis involving π**⋅⋅⋅**π and C−H**⋅⋅⋅**O interactions.

In the course of this scope investigation, it was noted that β‐borylacrylates that were unsubstituted in the β‐position were problematic (Figure 4). Indeed, exposing compound ***E*** **‐20** (R=H) to the general reaction conditions yielded the cross‐coupled product **22** exclusively, indicating that in situ isomerisation does not occur. This selectivity was also noted for ***E*** **‐21** (R=Me), generating product **23** in 58 %. Although attractive from the perspective of divergence, a solution was required to expand the substrate scope. To that end, a photocatalytic isomerisation of the β‐borylacrylate was performed under the auspices of selective energy transfer catalysis to generate substrates ***Z*** **‐20** and ***Z*** **‐21**.^[15]^ Exposure to the general condition reported above enabled formation of the desired coumarins **24** and **25** (55 % and 90 %, respectively). The direct annulation was employed in a short natural product synthesis as indicated in Figure 4 (bottom). Since the 3‐ and 4‐positions of angelicin (**26**) are unsubstituted, the *Z*‐configured C_3_‐synthon ***Z*** **‐20** was employed. Under the general conditions developed, this direct [3+3] annulation proceeded smoothly to deliver the target natural products Angelicin **26**. By employing the geometric isomer ***E*** **‐20**, it was possible to access compound **27** which is the core of Pongamol and Lanceolatin B.^[26]^

**Figure 4:**
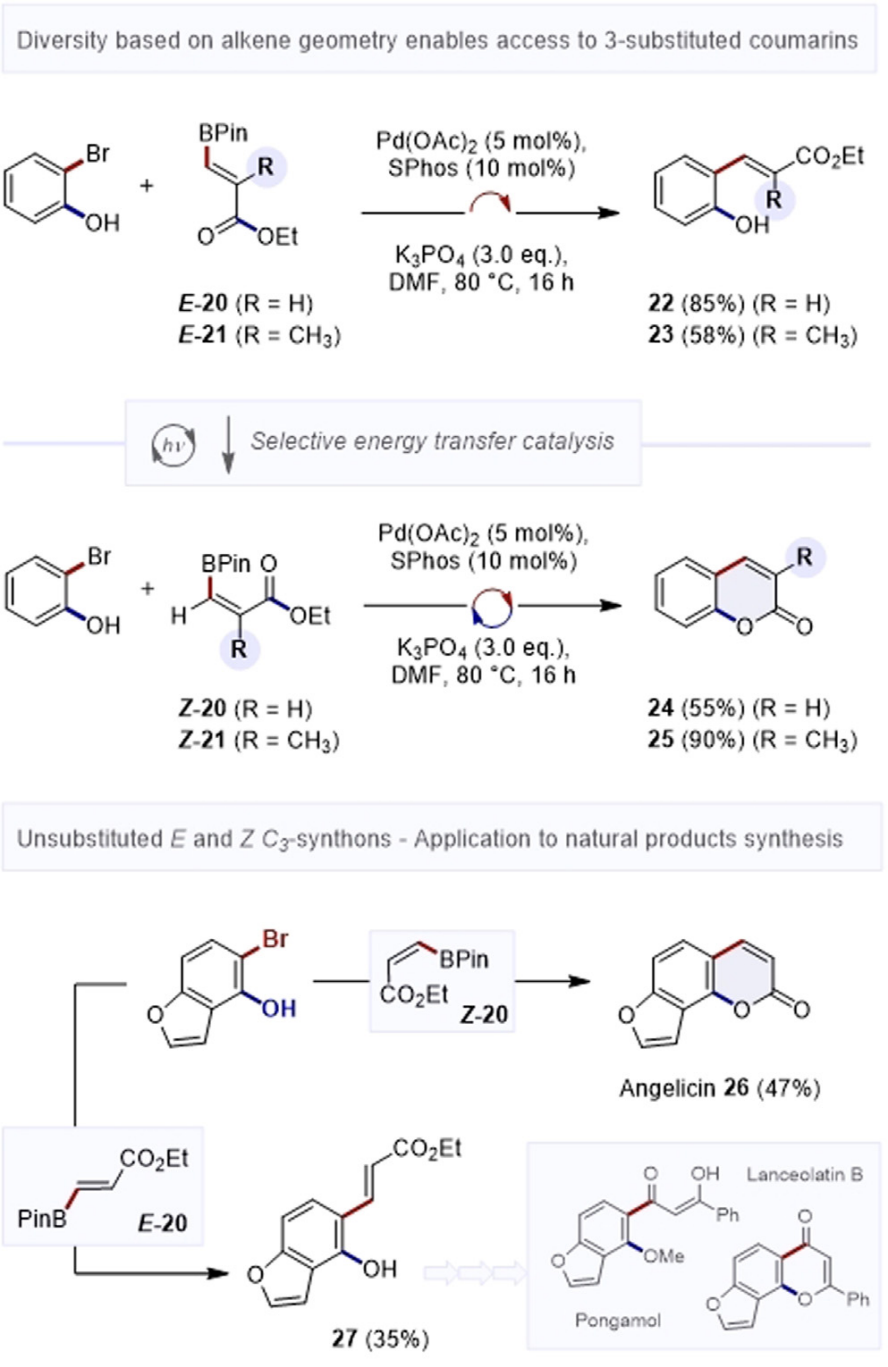
Merging energy transfer catalysis with the Suzuki–Miyaura/substitution cascade to enable divergence to 3‐substuted coumarins.

Finally, preliminary validation of the strategy in modulating the photophysical properties of selected systems was conducted (Figure 5). As illustrated, clear peaks that correspond to the coumarin scaffold are clearly visible in **14**, **16**, **17** and **18** which supports the notion that this simple annulation strategy based on β‐borylacrylates may find application in the development of tools for molecular imaging.

**Figure 5:**
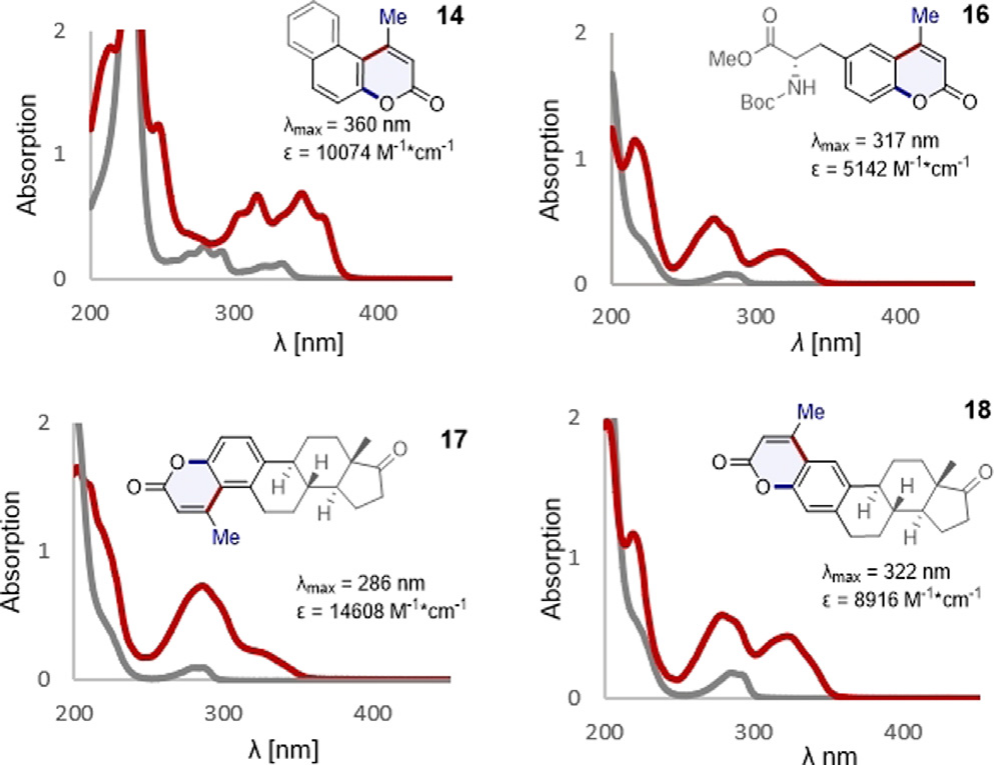
Comparative analysis of the UV/Vis spectra of selected 2‐halophenols (shown in grey) and the corresponding coumarin adducts **14**, **16**, **17** and **18** (shown in red).

In conclusion, a direct annulation of 2‐halophenols has been devised utilising simple, modular β‐borylacrylates as ambiphilic C_3_‐synthons. This study contributes to the venerable role of organoborons in creating well‐defined synthons for organic synthesis.[^27^, ^28^] In this modular platform, the boron substituent serves a dual purpose as both a traceless linker for C(sp^2^)–C(sp^2^) coupling, and as a chromophore extension to enable inversion of the alkene geometry via selective energy transfer catalysis. This latter attribute enables access to C‐3 and C‐4 unsubstituted coumarins and provides a basis for divergence. The transformation is operationally simple, displays high functional group tolerance and can be employed for late‐stage functionalisation. Application in short natural product syntheses is showcased and also in the facile conversion of the hormone estrone to a π‐extended analogue. It is envisaged that such transformations may be valuable in re‐purposing existing bioactive small molecules or in modifying them for analytical purposes.

## Conflict of interest

The authors declare no conflict of interest.

## Supporting information

As a service to our authors and readers, this journal provides supporting information supplied by the authors. Such materials are peer reviewed and may be re‐organized for online delivery, but are not copy‐edited or typeset. Technical support issues arising from supporting information (other than missing files) should be addressed to the authors.

Supplementary
