# Supplementary material for: Coumarins by Direct Annulation: β‐Borylacrylates as Ambiphilic C3‐Synthons
Source: Angew Chem Int Ed Engl. 2020 Nov 9;60(2):685–9. doi: 10.1002/anie.202012099 (PMC7839779; doi:10.1002/anie.202012099)
Supplement: Supplementary file 1 — Supplementary [file ANIE-60-685-s001.pdf]

Angewandte Chemie
Eine Zeitschrift der Gesellschaft Deutscher Chemiker

Supporting Information

Coumarins by Direct Annulation: β-Borylacrylates as Ambiphilic C_3-Synthons

*Max Wienhold, John J. Molloy, Constantin G. Daniliuc, and Ryan Gilmour**

anie_202012099_sm_miscellaneous_information.pdf

Table of Contents

1. General Information 3

2. Experimental Section 4

2.1 Synthesis of *ortho*-Bromophenols 4

2.2 Synthesis of *β*-Boryl Acrylates 13

2.3 Photocatalyzed Isomerization of *β*-Boryl Acrylates 20

2.4 Synthesis of Coumarin Derivatives 21

3. X-ray Crystallographic Analysis 32

4. NMR-Spectra of Key Compounds 36

5. References 78

1. General Information

Commercially available chemicals were purchased at *ABCR, Acros Organics, Sigma Aldrich, Alfa Aesar, fluorochem* or *Merck* and used as received. Solvents for purification (extraction and chromatography) were purchased as technical grade and distilled on the rotary evaporator prior to use. Dry solvents were obtained from a solvent purification system (activated Alumina under a positive pressure of argon). Degassed solvent refers to bubbling argon through the solvent for a minimum of 15 min. Analytical thin layer chromatography (TLC) was performed on plates from *Merck* (*silica gel 60, F_{254}*) and visualised with a UV lamp (λ = 254 nm and λ = 366 nm), $KMnO_4$ and CAM stain solutions. Column chromatography was carried out on silica gel 60 (40 -63 μm) from *Merck*. Concentration in vacuo was performed at ~10 mbar and 40 °C, drying at ~10^{-2} mbar and ambient temperature. NMR-spectra were recorded by the NMR service at the Organisch-Chemisches Institut, Westfälische Wilhelms-Universität Münster on a *Bruker AV300* (300 MHz)*, Bruker AV400* (400 MHz), *Agilent DD2 500* (500 MHz) and an *Agilent DD2 600* (600 MHz). Chemical shifts δ are stated in ppm and referenced to the residual solvent signal. The resonance multiplicity is abbreviated as: s (singlet), bs (broad singlet), d (doublet), t (triplet), q (quadruplet), quint(quintet), sext (sextet), sep (septet), m (multiplet). Assignments of unknown compounds are based on DEPT, COSY (HH), HMBC, HSQC and NOESY spectra. IR measurements were carried out on a *Perkin-Elmer 100 FT-IR spectrometer* and the intensities of the bands are assigned as follows: w (weak), m (medium), s (strong). High-resolution mass spectra were measured by the MS service of the Organisch-Chemisches Institut, Westfälische Wilhelms-Universität Münster. Melting points were determined on a *Büchi B-545* melting-point apparatus in open capillaries. Optical rotations were measured on a JASCO P2000 polarimeter. UV/vis absorption spectra were measured on an Agilent Cary 60 UV-Vis Spectrophotometer, baseline correction was performed with the corresponding solvent. Photoreactions were performed utilizing a set-up of 4 Winger WEPIV3-S2 UV Power LED Star (Schwarzlicht) 1.2 W lamps (emission spectrum see Figure 1). The forward current per chip was set to 700 mA, the resulting forward voltage was 3.4 V while the resulting radiant flux was 1200 mW. The distance between the reaction vessels and the UV-lamp was set at approximately 0.5 cm for all reactions.

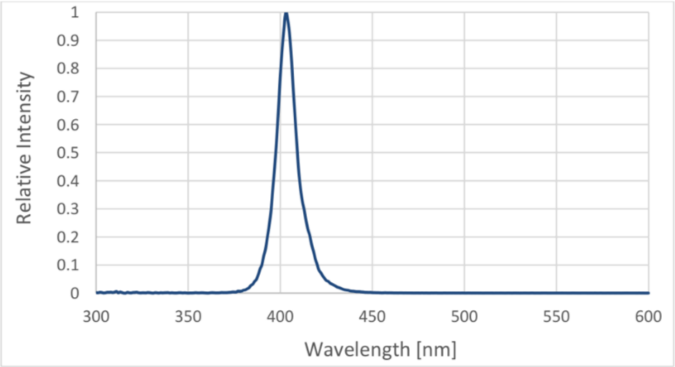

Figure 1: Emission spectrum of the utilised a *Winger WEPUV3-S2 UV Power LED Star* (402 nm).

2. Experimental Section

2.1 Synthesis of *ortho*-Bromophenols

Synthesis of 5-amino-2-bromophenol (S1)

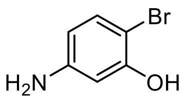

2-Bromo-5-nitrophenol (109 mg, 0.5 mmol, 1.0 eq) was dissolved in water (3 mL) and EtOH (3 mL) before the addition of NH_4Cl (147.1 mg, 2.75 mmol, 5.5 eq.) and Fe-powder (167.4 mg, 3.0 mmol, 6.0 eq). The reaction mixture was stirred at 80 °C for 5 h. After cooling to ambient temperature the mixture was filtered through a plug of Celite and the solvent was evaporated under reduced pressure. The crude residue was purified by column chromatography (SiO_2, DCM) to give the desired product as a yellow solid (56 mg, 60%).

R_f = 0.16 (DCM)

^{1}H-NMR (300 MHz, $CDCl_3$): δ (ppm) = 7.17 (d, J = 8.6 Hz, 1H), 6.36 (d, J = 2.6 Hz, 1H), 6.18 (dd, J = 8.6, 2.6 Hz, 1H), 5.37 (br s, 1H) 3.68 (br s, 2H).

Analytical data in agreement with literature data.[1]

Synthesis of 2-bromo-4-methoxyphenol (S2)

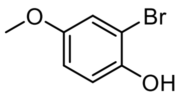

4-Methoxyphenol 496.6 mg, 4.0 mmol, 1.0 eq.) was dissolved in MeCN (32 mL) and *N*-bromosuccinimide (708 mg, 4.0 mmol, 1.0 eq.) was added. The mixture was stirred at ambient temperature for 2 h before the solvent was evaporated under reduced pressure. The crude residue was purified by column chromatography (SiO_2, 10% EtOAc/*n*-pentane) to give the desired product as a red oil (204.0 mg, 25%).

R_f = 0.34 (EtOAc/*n*-pentane 1:9)

^{1}H-NMR (400 MHz, $CDCl_3$): δ (ppm) = 7.01 (d, *J* = 2.9 Hz, 1H), 6.94 (d, *J* = 8.9 Hz, 1H), 6.80 (dd, *J* = 8.9, 2.9 Hz, 1H), 5.13 (s, 1H), 3.75 (s, 3H).

Analytical data in agreement with literature.[2]

Synthesis of 4-(naphthalen-1-yl)phenol (S3)

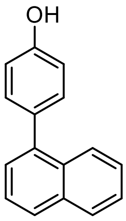

A Schlenk-tube, equipped with naphthalen-1-ylboronic acid (413 mg, 2.4 mmol, 1.2 eq.), 4-bromophenol (346 mg, 2.0 mmol, 1.0 eq.), $Pd(PPh_3)_4$ (116 mg, 0.1 mmol, 5 mol%) and K_2CO_3 (1.38 g, 10.0 mmol, 5.0 eq.), was sealed and purged with argon. Subsequently degassed DMF (16 mL) and water (4 mL) were added and the mixture was stirred at 80 °C for 14 h. After cooling to ambient temperature the mixture was diluted with water (20 mL) and the organics were extracted with EtOAc (3 x 25 mL). The combined organic layers were washed with brine (20 mL), dried over $MgSO_4$ and evaporated under reduced pressure. The crude residue was purified by

column chromatography (SiO_2, 10% Et_2O/*n*-pentane) to give the desired product as a sticky colourless solid (370 mg, 85%).

R*f* = 0.21 (EtOAc/*n*-pentane 1:9).

^{1}H-NMR (300 MHz, $CDCl_3$): δ (ppm) = 7.91 (tt, *J* = 6.6, 0.8 Hz, 2H), 7.84 (d, *J* = 8.3 Hz, 1H), 7.54 – 7.41 (m, 4H), 7.38 (d, *J* = 8.5 Hz, 2H), 6.96 (d, *J* = 8.5 Hz, 2H), 4.83 (bs, 1H).

Analytical data in agreement with literature data.[3]

Synthesis of 2-bromo-4-(naphthalen-1-yl)phenol (S4)

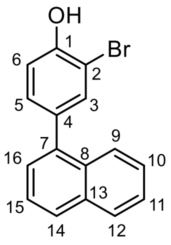

S3 (220 mg, 1.0 mmol, 1.0 eq.), *N*-bromosuccinimide (178 mg, 1.0 mmol, 1.0 eq.) and $AlCl_3$ (13.3 mg, 0.1 mmol, 10 mol%) were dissolved in DCM (5 mL) and the mixture was stirred at ambient temperature for 24 h. After the reaction was complete, water (5 mL) was added and organics were extracted with EtOAc (3 x 10 mL). The combined organic layers were dried over $MgSO_4$, evaporated under reduced pressure and the crude residue was purified by column chromatography (2% EtOAc/*n*-pentane) to give the desired product as a colourless gum (81 mg, 27%).

R*f* = 0.37 (SiO_2, EtOAc/*n*-pentane 1:9)

^{1}H-NMR (599 MHz, $CDCl_3$): δ (ppm) = 7.95 – 7.86 (m, 3H, H9, H12, H14), 7.64 (d, J = 2.1 Hz, 1H, H3), 7.56 – 7.44 (m, 3H, H10, H11, H15), 7.42 – 7.35 (m, 2H, H5, H16), 7.17 (d, J = 8.3 Hz, 1H, H6), 5.68 (s, 1H, O*H*).

^{13}C-NMR (126 MHz, $CDCl_3$): δ (ppm) = 151.7 (C1), 138.5 (C7), 134.8 (C4), 133.9 (C13), 133.3 (C3), 131.7 (C8), 131.1 (C5), 128.5 (C9), 128.0 (C14), 127.2 (C16), 126.4 (C10), 126.0 (C15), 125.8 (C9), 125.5 (C11), 115.9 (C6), 110.2 (C2).

IR (ATR) $\tilde{\nu}$ = 3354 (w), 3056 (w), 1603 (w), 1495 (m), 1393 (m), 1329 (w), 1276 (m), 1249 (w), 1175 (m), 1040 (m), 973 (w), 827 /m), 800 (s), 776 (s), 697 (s).

HR-ESI-MS exact mass calculated for $[M-H]^-$ $(C16H10BrO)^-$ requires m/z 296.9921, found m/z 296.9929.

Synthesis of methyl(*S*)-3-(3-bromo-4-hydroxyphenyl)-2-((*tert*-butoxycarbonyl)amino)propanoate (S5)

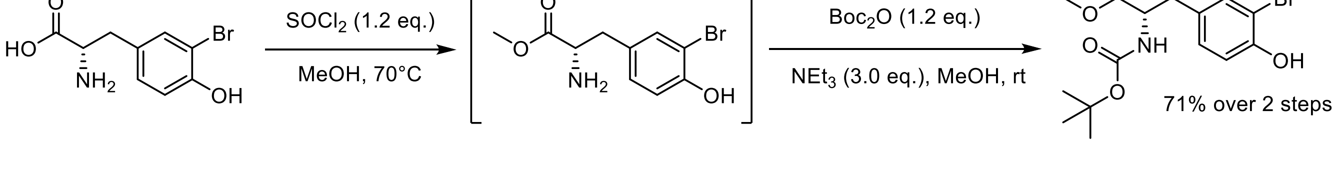

According to a procedure by Restrepo *et al.*[4], $SOCl_2$ (72 μL, 119 mg, 1.1 mmol, 1.1 eq.) was added to MeOH (2 mL) at -5 °C before the addition of 3-bromotyrosine (260 mg, 1.0 mmol, 1.0 eq.). The reaction mixture was heated to 70 °C with stirring for 8 h. After the reaction was complete, the mixture was cooled

to ambient temperature and stirred for an additional 10 h. After the addition of Et_2O (1 mL), the formed precipitate was filtered off and dried under reduced pressure. The crude product was dissolved in MeOH (2 mL) before the addition of Boc_2O (262 mg, 1.2 mmol, 1.2 eq.) and NEt_3 (415 μL, 303 mg, 3.0 mmol, 3.0 eq.). The reaction mixture was stirred at ambient temperature for 24 h. After the reaction was complete, the mixture was adjusted to pH = 3 by the addition of HCl (1 M) and organics were extracted with EtOAc (3x10 mL). The combined organics were dried over $MgSO_4$ and the solvent was evaporated under reduced pressure. The crude residue was purified by column chromatography (SiO_2, 30% EtOAc/*n*-pentane) to give the desired product as a colourless solid (269 mg, 71%).

R_f = 0.53 (30 % EtOAc/*n*-pentane).

^{1}H-NMR (300 MHz, $CDCl_3$): δ (ppm) = 7.23 (d, *J* = 1.9 Hz, 1H), 6.98 (dd, *J* = 8.3, 1.9 Hz, 1H), 6.93 (d, *J* = 8.3 Hz, 1H), 5.50 (s, 1H), 5.00 (d, *J* = 8.2 Hz, 1H), 4.52 (q, *J* = 6.6 Hz, 1H), 3.72 (s, 3H), 3.05 (dd, J = 14.0, 5.7 Hz, 1H), 2.95 (dd, *J* = 14.0, 6.0 Hz, 1H), 1.43 (s, 9H).

ORD ($CHCl_3$, c 1.00): $[\alpha]_D^{24}$= 24.023.

Analytical data in agreement with literature data.[4]

Bromination of estrone

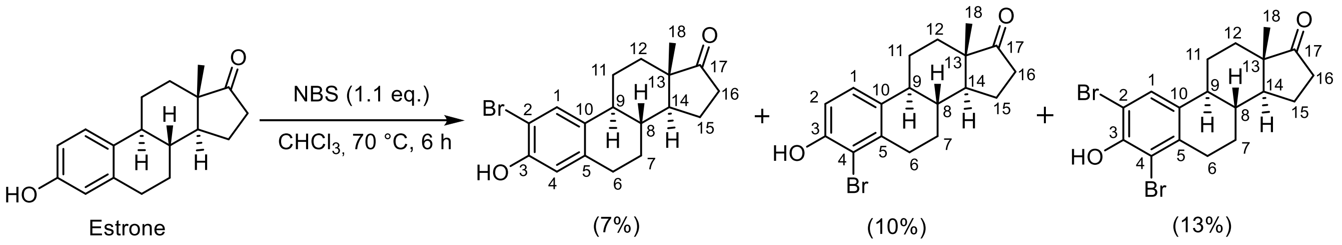

Estrone (811 mg, 3.0 mmol, 1.0 eq.) was dissolved in $CHCl_3$ (20 mL) and *N*-bromosuccinimide (588 mg, 3.3 mmol, 1.1 eq.) was added. The mixture was heated to 70 °C with stirring for 6 h. After cooling to ambient temperature, EtOAc (50 mL) was added and the organic layer was washed with brine (20 mL), dried over $MgSO_4$ and the solvent was evaporated under reduced pressure. The crude residue was purified by column chromatography (SiO_2, 2% to 10% EtOAc/*n*-pentane) to give several regioisomers.

2-Bromoestrone (S6)

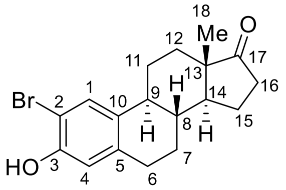

The product was obtained as a colourless solid (68 mg, 7%)

R_f = 0.31 (EtOAc/*n*-pentane 1:9).

^{1}H-NMR (599 MHz, $CDCl_3$): δ (ppm) = 7.34 (s, 1H, H1), 6.76 (s, 1H, H4), 5.29 (s, 1H, OH), 2.85 – 2.81 (m, 2H, H6), 2.51 (ddd, *J* = 19.2, 8.9, 1.1 Hz, 1H, $H16_A$), 2.35 – 2.29 (m, 1H, $H11_A$), 2.22 (td, *J* = 10.7, 3.9 Hz, 1H, H9), 2.14 (dt, *J* = 19.1, 9.0 Hz, 1H, $H16_B$), 2.08 – 2.03 (m, 1H, $H15_A$), 2.02 – 1.92 (m, 2H, $H7_A$, $H12_A$), 1.65 – 1.59 (m, 1H, $H15_B$), 1.56 – 1.36 (m, 5H, $H7_B$, H8, $H11_B$, $H12_B$, H14), 0.91 (s, 3H, H18).

^{13}C-NMR (126 MHz, $CDCl_3$): δ (ppm) = 220.8 (C17), 150.2 (C3), 138.1 (C5), 134.0 (C10), 128.9 (C1), 116.0 (C4), 107.6 (C2), 50.5 (C14), 48.1 (C13), 43.9 (C9), 38.2 (C8), 36.0 (C16), 31.6 (C12), 29.2 (C6), 26.4 (C7), 26.1 (C11), 21.7 (C15), 14.0 (C18).

IR (ATR) $\tilde{\nu}$ = 3316 (m), 2924 (m), 2858 (w), 1723 (s), 1600 (w), 1500 (w), 1471 (m), 1405 (s), 1257 (s), 1206 (s), 1165 (m), 1083 (m), 1054 (m), 970 (w), 949 (w), 880 (s), 827 (m), 795 (m), 735 (m), 668 (s) cm^{-1}.

HR-ESI-MS (Orbitrap) exact mass calculated for $[M-H]^-$ $(C_{18}H_{20}O_2Br)^-$ requires m/z 347.06522, found m/z 347.06497.

M.p.: 208.9 - 211.9 °C.

ORD ($CHCl_3$, c 1.00): $[\alpha]_D^{23}$= 140.850.

4-Bromoestrone (S7)

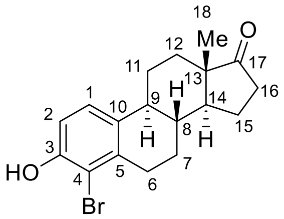

The product was obtained as a colourless solid (102 mg, 10%)

R_f = 0.34 (EtOAc/*n*-pentane 1:9)

^{1}H-NMR (599 MHz, $CDCl_3$): δ (ppm) = 7.19 (d, *J* = 8.5 Hz, 1H, H1), 6.87 (d, *J* = 8.5 Hz, 1H, H2), 5.54 (s, 1H, OH), 2.97 (dd, J = 17.7, 6.2 Hz, 1H, $H6_A$), 2.73 (ddd, *J* = 17.7, 12.2, 7.0 Hz, 1H, $H6_B$), 2.51 (ddd, *J* = 18.9, 8.9, 1.1 Hz, 1H, $H16_A$), 2.39 (qd, J = 4.7, 2.7 Hz, 1H, $H11_A$), 2.27 (dt, *J* = 10.4, 5.4 Hz, 1H, H9), 2.18 – 2.05 (m, 3H, $H7_A$, $H15_A$, $H16_B$), 1.99 – 1.92 (m, 1H, $H12_A$), 1.66 – 1.60 (m, 1H, $H15_B$), 1.56 – 1.47 (m, 4H, $H7_B$, H8, $H11_B$, $H12_B$), 1.45 – 1.39 (m, 1H, H14), 0.90 (s, 3H).

^{13}C-NMR (126 MHz, $CDCl_3$): δ (ppm) = 220.8 (C17), 150.4(C3), 136.4 (C5), 133.9 (C10), 125.7(C1), 113.9 (C2), 113.0 (C4), 50.4 (C14), 48.0 (C13), 44.3 (C9), 37.7 (C8), 36.0 (C16), 31.7 (C12), 31.2 (C6), 26.8 (C7), 26.3 (C11), 21.7 (C15), 13.9 (C18).

IR (ATR) $\tilde{\nu}$ = 3420 (s), 2936 (m), 2919 (m), 1729 (s), 1594 (m), 1476 (s), 1409 (m), 1341 (w), 1310 (w), 1279 (m), 1264 (m), 1212 (w), 1179 (m), 1167 (s), 1082 (w), 1058 (w), 1044 (w), 1013 (w), 835 (w), 819 (m), 796 (w), 656 (w) cm^{-1}.

HR-ESI-MS (Orbitrap) exact mass calculated for $[M-H]^-$ $(C_{18}H_{20}O_2Br)^-$ requires m/z 347.06522, found m/z 347.06570.

M.p.: 269.1 - 273.4 °C.

ORD ($CHCl_3$, c 1.00): $[\alpha]_D^{23}$= 104.195.

2,4-Bromoestrone (S8)

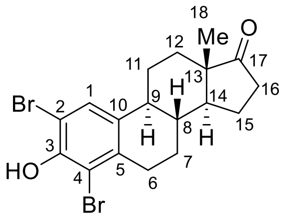

The product was obtained as a colourless solid (160 mg, 13%)

R_f = 0.60 (EtOAc/*n*-pentane 1:9)

^{1}H-NMR (599 MHz, $CDCl_3$): δ (ppm) = 7.41 (s, 1H, H1), 5.84 (s, 1H, OH), 2.98 – 2.90 (m, 1H, $H6_A$), 2.73 – 2.64 (m, 1H, $H6_B$), 2.52 (ddd, *J* = 19.1, 9.0, 1.1 Hz, 1H, $H16_A$), 2.38 – 2.31 (m, 1H, $H11_A$), 2.30 – 2.22 (m, 1H, H9), 2.20 – 2.03 (m, 3H, $H7_A$, $H15_A$, $H16_B$), 2.00 – 1.95 (m, 1H, $H12_A$), 1.63 (tt, *J* = 12.1, 9.0 Hz, 1H, $H15_B$), 1.57 – 1.37 (m, 5H, $H7_B$, H8, $H11_B$, $H12_B$, H14), 0.90 (s, 3H, H18).

^{13}C-NMR (126 MHz, $CDCl_3$): δ (ppm) = 220.5 (C17), 147.4 (C3), 136.6 (C5), 135.2 (C10), 128.7 (C1), 113.4 (C4), 106.6 (C2), 50.4 (C14), 47.9 (C13), 44.1 (C9), 37.5 (C8), 36.0 (C16), 31.5 (C12), 31.1 (C6), 26.6 (C7), 26.3 (C11), 21.7 (C15), 13.9 (C18).

IR (ATR) $\tilde{\nu}$ = 3383 (w), 2934 (m), 2873 (m), 1734 (s), 1541 (w), 1465 (s), 1400 (m), 1330 (w), 1277 (m), 1261 (m), 1173 (m), 1086 (w), 1058 (w), 1013 (w), 900 (w), 830 (w), 761 (s) cm^{-1}.

HR-ESI-MS (Orbitrap) exact mass calculated for $[M-H]^-$ $(C_{18}H_{19}O_2Br_2)^-$ requires m/z 426.97268, found m/z 426.97332.

M.p.: 232.4 – 235.1 °C.

ORD ($CHCl_3$, c 1.00): $[\alpha]_D^{23}$= 110.067.

Synthesis of 2-bromo-3-((tert-butyldimethylsilyl)oxy)-4-methoxybenzaldehyde (S9)

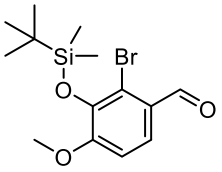

According to a procedure by Schäfer *et al*.[5], 3-hydroxy-4-methoxybenzaldehyde (1.2 g, 8.0 mmol, 1.0 eq.), NaOAc (1.3 g, 16.0 mmol, 2.0 eq.) and Fe-powder (36.0 mg, 0.64 mmol, 0.08 eq.) were suspended in acetic acid (1.0 M, 8 mL) under an argon atmosphere. Subsequently, a solution of Br_2 (1.3 g, 0.42 mL, 8.4 mmol, 1.05 eq.) in acetic acid (2mL) was added dropwise and the mixture was stirred at ambient temperature for 1 h before it was poured into ice-water (20 mL). The formed precipitate was filtered-off, washed with ice-cold water and dried under reduced pressure. The crude product was dissolved in DMF (80 mL), placed under an argon atmosphere and cooled to 0 °C before the addition of *N,N*-Diisopropylethylamine (4.1 mL, 3.1 g, 24.0 mmol, 3.0 eq.). The reaction mixture was stirred for 15 min at 0 °C before the addition of TBDMSCl (1.4 g, 9.6 mmol, 1.2 eq.) in small portions. The mixture was warmed to ambient temperature and was stirred for an additional 2 h. The reaction was quenched by the addition of water (50 mL) and organics were extracted with EtOAc (3 x 120 mL). The combined organic layers were dried over $MgSO_4$ and the solvent was evaporated under reduced pressure. The crude residue was purified by column chromatography (SiO_2, 0% to 10% EtOAc/*n*-pentane) to give the desired product as a colorless oil (1.59 g, 58%).

R_f = 0.20 (EtOAc/*n*-pentane 1:9).

^{1}H-NMR (400 MHz, $CDCl_3$): δ (ppm) = 10.27 (d, *J* = 0.8 Hz, 1H), 7.59 (d, *J* = 8.6 Hz, 1H), 6.88 (dd, *J* = 8.6, 0.8 Hz, 1H), 3.88 (s, 3H), 1.05 (s, 9H), 0.23 (s, 6H).

Analytical data in agreement with literature.[5]

Synthesis of 5-(bromomethyl)-1,2,3-trimethoxybenzene (S10)

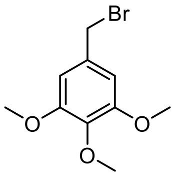

To a solution of 3,4,5-trimetoxybenzylic alcohol (594.7 mg, 3.0 mmol, 1.0 eq.) and CBr_4 (1.04 g, 3.15 mmol, 1.05 eq.) in DCM (0.2 M, 15 mL), PPh_3 (856 mg, 3.3 mmol, 1.1 eq.) was added in small portions. The reaction mixture was stirred at ambient temperature for 1 h before the solvent was evaporated under reduced pressure. The crude residue was purified by column chromatography (SiO_2, 10% EtOAc/*n*-pentane) to give the desired product as a white solid (590 mg, 75%).

R_f = 0.63 (EtOAc/*n*-pentane 1:9)

^{1}H-NMR (400 MHz, $CDCl_3$): δ (ppm) = 6.62 (s, 2H), 4.46 (s, 2H), 3.87 (s, 6H), 3.84 (s, 3H).

Analytical data in agreement with literature.[6]

Synthesis of triphenyl(3,4,5-trimethoxybenzyl)phosphonium bromide (S11)

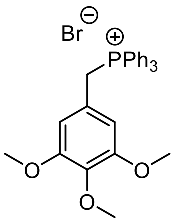

To a solution of PPh_3 (526.0 mg, 2.1 mmol, 1.05 eq.) in toluene (0.3 M, 7 mL), **S10** (522.0 mg, 2.0 mmol, 1.0 eq.) was added. The mixture was stirred at 110 °C for 6 h. After the reaction was complete the mixture was cooled to 0 °C and the formed precipitate was filtered-off and washed with ice-cold toluene. The residue was dried under reduced pressure to give the desired product as a colorless solid (879.0 mg, 84%).

^{1}H-NMR (400 MHz, $CDCl_3$): δ (ppm) = 7.80 – 7.67 (m, 9H), 7.67 – 7.52 (m, 6H), 6.48 (d, *J* = 2.7 Hz, 2H), 5.40 (d, *J* = 14.1 Hz, 2H), 3.75 (s, 3H), 3.49 (s, 6H).

^{31}P-NMR (162 MHz, $CDCl_3$): δ (ppm) = 23.18.

Analytical data in agreement with literature.[6]

Synthesis of (*Z*)-(2-bromo-6-methoxy-3-(3,4,5-trimethoxystyryl)phenoxy)(tert-butyl) dimethyl silane (S12) and (*E*)-(2-bromo-6-methoxy-3-(3,4,5-trimethoxystyryl)phenoxy)(tert-butyl) dimethylsilane (S13)

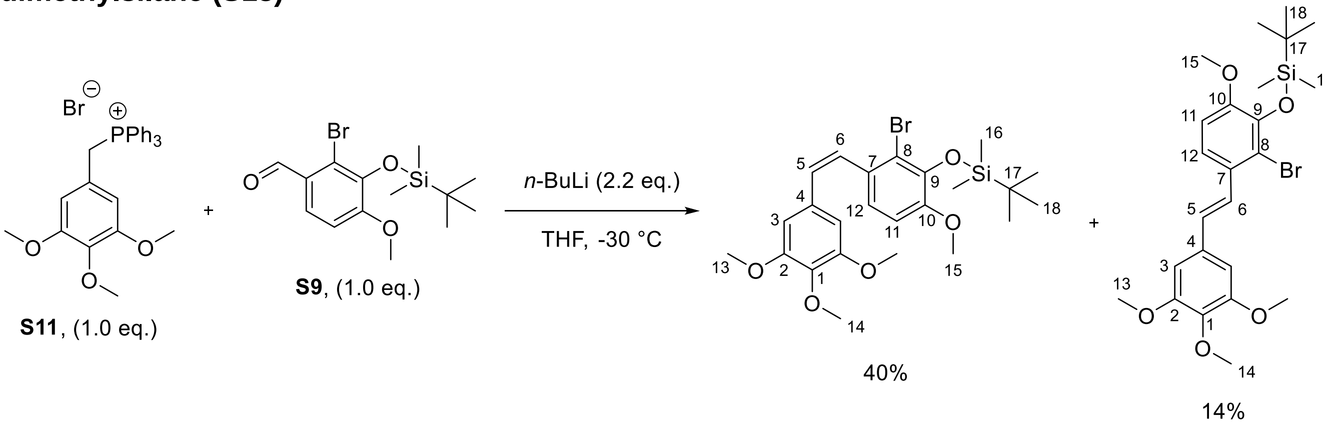

According to a procedure by Pritchard *et al*.[7], **S11** (690.6 mg, 2.0 mmol, 1.2 eq.) was dissolved in dry THF (0.2 M, 5 mL) under an argon atmosphere and the solution was cooled to -30 °C. At that temperature *n*BuLi (1.6 M solution in hexanes, 1.4 mL, 2.2 mmol, 2.2 eq.) was added dropwise and the dark red mixture was stirred for 15 min. Subsequently, **S9** (231.1 mg, 1.0 mmol, 1.0 eq.) was added dropwise as a solution in dry THF (2 mL) and the mixture was stirred at -30 °C for an additional 2 h. The reaction was allowed to warm to ambient temperature and was quenched by the addition of sat. aq. NH_4Cl (5 mL) and organics were extracted with EtOAc (3 x 20 mL). The combined organic layers were dried over $MgSO_4$ and the solvent was evaporated under reduced pressure. The crude residue was purified by column chromatography (SiO_2, 10% EtOAc/*n*-pentane) to afford two alkene isomers.

(*Z*)-(2-bromo-6-methoxy-3-(3,4,5-trimethoxystyryl)phenoxy)(tert-butyl)dimethylsilane (S12)

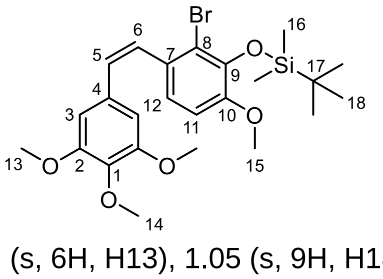

The product was obtained as a colorless solid (405.0 mg, 40%).
R_f = 0.35 (10% EtOAc/*n*-pentane)
^{1}H-NMR (599 MHz, $CDCl_3$): δ (ppm) = 6.82 (d, *J* = 8.5 Hz, 1H, H12), 6.66 (d, *J* = 8.5 Hz, 1H, H11), 6.54 (d, *J* = 11.9 Hz, 1H, H6), 6.49 (d, *J* = 12.0 Hz, 1H, H5), 6.40 (s, 2H, H3), 3.81 (s, 3H, H14), 3.77 (s, 3H, H15), 3.63 (s, 6H, H13), 1.05 (s, 9H, H18), 0.22 (s, 6H, H16).
^{13}C-NMR (126 MHz, $CDCl_3$): δ (ppm) = 152.9 (C2), 150.0 (C10), 143.1 (C8), 137.3 (C1), 132.3 (C4), 131.5 (C9), 130.1 (C5), 129.8 (C6), 122.9 (C12), 117.4 (C7), 110.3 (C11), 106.3 (C3), 61.0 (C14), 55.9 (C13), 55.4 (C15), 26.2 (C17), 19.2 (C17), -3.7 (C16).
Ir (ATR) $\tilde{\nu}$ = 2999 (w), 2957 (m), 2933 (m), 2857 (m), 1687 (w), 1581 (m), 1506 (m), 1485 (s), 1463 (m), 1420 (s), 1407 (m), 1327 (m), 1305 (s), 1256 (s), 1239 (s), 1187 (m), 1130 (s), 1034 (m), 1002 (s), 967 (m), 863 (s), 840 (s), 813 (m), 782 (s)661 (m).

HR-ESI-MS (Orbitrap) exact mass calculated for $[M+Na]^+$ $(C_{24}H_{33}O_5BrSiNa)^+$ requires m/z 531.11728, found m/z 531.11731.

M.p.: 77.3 – 78.8 °C.

(*E*)-(2-bromo-6-methoxy-3-(3,4,5-trimethoxystyryl)phenoxy)(tert-butyl)dimethylsilane (S13)

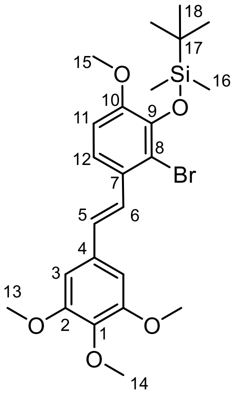

The product was obtained as a colorless solid (141.0 mg, 14%).

R*f* = 0.23 (10% EtOAc/*n*-pentane)

^{1}H-NMR (599 MHz, $CDCl_3$): δ (ppm) = 7.34 (d, *J* = 16.1 Hz, 1H, H6), 7.22 (d, *J* = 8.6, 1H, H12), 6.83 (d, *J* = 16.1 Hz, 1H, H5), 6.82 (d, *J* = 8.6 Hz, 1H, H11), 6.74 (s, 2H, H3), 3.92 (s, 6H, H13), 3.87 (s, 3H, H14), 3.82 (s, 3H, H15), 1.06 (s, 9H, H18), 0.24 (s, 6H, H16).

^{13}C-NMR (126 MHz, $CDCl_3$): δ (ppm) = 153.5 (C2), 150.4 (C10), 142.9 (C8), 138.1 (C1), 133.4 (C4), 131.0 (C9), 129.5 (C5), 127.9 (C6), 118.8 (C12), 117.9 (C7), 110.6 (C11), 103.9 (C3), 61.1 (C14), 56.3 (C13), 55.4 (C15), 26.2 (C18), 19.2 (C17), -3.6 (C16).

IR (ATR) $\tilde{\nu}$ = 2931 (w), 2856 (w), 1580 (m), 1509 (m), 1484 (m), 1462 (m), 1441 (m), 1420 (m), 1362 (w), 1340 (m), 1307 (m), 1245 (s), 1175 (m), 1128 (s), 1031 (s), 1009 (s), 960 (m), 865 (m), 839 (m), 812 (s), 781 (s), 716 (m), 666 (m).

HR-ESI-MS (Orbitrap) exact mass calculated for $[M+Na]^+$ $(C_{24}H_{33}O_5BrSiNa)^+$ requires m/z 531.11728, found m/z 531.11743.

M.p.: 106.0 – 107.5 °C.

Synthesis of (*Z*)-2-bromo-6-methoxy-3-(3,4,5-trimethoxystyryl)phenol (S14)

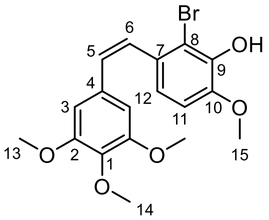

S12 (254.8 mg, 0.5 mmol, 1.0 eq.) was dissolved in dry THF (0.1 M, 5 mL) under an argon atmosphere and the solution was cooled to 0 °C before the addition of TBAF (1.0 M solution in THF, 0.5 mL, 0.5 mmol, 1.0 eq.). The reaction mixture was stirred at ambient temperature for 20 min. The reaction was quenched by the addition of ice-cold water and organics were extracted with Et_2O (1 x 25 mL). The organic layer was separated, washed with water (3 x 10 mL) and dried over $MgSO_4$. The solvent was evaporated under reduced pressure and the crude residue was purified by column chromatography (40% EtOAc/cyclohexane) to obtain the desired product as a colorless solid (128.1 mg, 64%).

R*f* = 0.54 (40% EtOAc/cyclohexane).

^{1}H-NMR (599 MHz, $CDCl_3$): δ (ppm) = 6.81 (d, *J* = 8.4 Hz, 1H, H11), 6.69 (d, *J* = 8.5 Hz, 1H, H12), 6.53 (s, 2H, H5/H6), 6.40 (s, 2H, H3), 5.99 (s, 1H, OH), 3.87 (s, 3H, H15), 3.81 (s, 3H, H14), 3.64 (s, 6H, H13).

^{13}C-NMR (151 MHz, $CDCl_3$): δ (ppm) = 152.8 (C2), 146.0 (C10), 143.2 (C9), 137.3 (C1) 132.0 (C4), 131.2 (C8), 130.7 (C5/C6), 128.7 (C5/C6), 121.5 (C11), 110.2 (C7), 109.5 (C12), 106.2 (C3), 60.9 (C14), 56.4 (C15), 55.8 (C13).

IR (ATR) $\tilde{\nu}$ = 3385 (w), 3304 (w), 2967 (w), 2938 (w), 1582 (m), 1508 (m), 1489 (m), 1421 (s), 1333 (m), 1278 (m), 1238 (s), 1187 (m), 1125 (s), 1031 (s), 996 (s), 860 (m), 821 (m), 803 (m), 777 (m), 705 (m), 658 (m).

HR-ESI-MS (Orbitrap) exact mass calculated for $[M+Na]^+$ $(C_{18}H_{19}O_5BrNa)^+$ requires m/z 417.03081, found m/z 417.03040.

M.p.: 137.4 – 140.2 °C

Synthesis of 5-bromo-6,7-dihydrobenzofuran-4(5*H*)-one (S15)

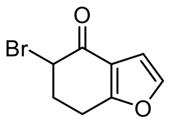

A pressure tube was equipped with 6,7-dihydrobenzofuran-4(5*H*)-one (680.8 mg, 5.0 mmol, 1.0 eq.) and $CuBr_2$ (2.46 g, 11.0 mmol, 2.2 eq.) and purged with argon before the addition of $CHCl_3$ (8 mL) and EtOAc (8 mL). The reaction mixture was stirred at 80 °C for 16 h. After completion, the reaction was cooled to ambient temperature and the mixture was filtered over Celite, eluting with EtOAc (25 mL). The solvent was evaporated under reduced pressure and the crude residue was purified by column chromatography (SiO_2, 0% - 10% EtOAc/*n*-pentane) to give the desired product as a colourless oil (550.0 mg, 51%).

R_f = 0.31 (EtOAc/*n*-pentane 1:9).

^{1}H-NMR (300 MHz, $CDCl_3$): δ (ppm) = 7.37 (d, *J* = 2.1 Hz, 1H), 6.72 (d, *J* = 2.0 Hz, 1H), 4.56 (t, *J* = 3.8 Hz, 1H), 3.20 – 3.05 (m, 1H), 2.88 (dt, *J* = 17.7, 4.4 Hz, 1H), 2.58 – 2.46 (m, 2H).

Analytical data in agreement with literature.[8]

Synthesis of 5,5-dibromo-6,7-dihydrobenzofuran-4(5*H*)-one (S16)

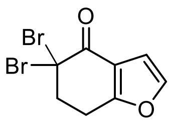

A pressure tube was equipped with **S15** (1.0 g, 5.0 mmol, 1.0 eq.) and $CuBr_2$ (3.35 g, 15.0 mmol, 3.0 eq.) and purged with argon before the addition of $CHCl_3$ (8 mL) and EtOAc (8 mL). The reaction mixture was stirred at 80 °C for 16 h. After completion, the reaction was cooled to ambient temperature and the mixture was filtered over Celite, eluting with EtOAc (25 mL). The solvent was evaporated under reduced pressure and the crude residue was purified by column chromatography (SiO_2, 5% EtOAc/*n*-pentane) to give the desired product as a pink solid (428.9 mg, 29%).

R_f = 0.43 (EtOAc/*n*-pentane 1:9).

^{1}H-NMR (400 MHz, $CDCl_3$): δ (ppm) = 7.39 (d, *J* = 2.1 Hz, 1H), 6.76 (d, *J* = 2.1 Hz, 1H), 3.13 (t, *J* = 5.5 Hz, 2H), 3.01 (t, *J* = 5.7 Hz, 2H).

Analytical data in agreement with literature.[9]

Synthesis of 5-bromobenzofuran-4-ol (S17)

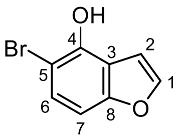

S16 (293.9 mg, 1.0 mmol, 1.0 eq.) was dissolved in MeCN (7 mL) and the solution was cooled to 0°C before the addition of DBU (240 μL, 1.6 mmol, 1.6 eq.). The mixture was allowed to warm up to ambient temperature and was stirred for additional 16 h. After completion, the reaction was quenched by sat. aq. NH_4Cl (5 mL) and organics were extracted with DCM (3 x 25 mL). The combined organic layers were washed with brine (10 mL), dried over $MgSO_4$ and the solvent was evaporated under reduced pressure. The crude residue was purified by column chromatography (15% Et_2O/*n*-pentane) to give the desired product as a white solid (164.9 mg, 77%).

R_f = 0.58 (15% EtO/*n*-pentane).

^{1}H-NMR (500 MHz, $CDCl_3$): δ (ppm) = 7.55 (d, *J* = 2.2 Hz, 1H, H1), 7.34 (d, *J* = 8.7, 1H, H6), 7.03 (dd, *J* = 8.7, 1.0 Hz, 1H, H7), 6.89 (dd, *J* = 2.2, 0.9 Hz, 1H, H2), 5.80 (s, 1H, OH).

^{13}C-NMR (126 MHz, $CDCl_3$): δ (ppm) = 155.9 (C8), 146.0 (C4), 144.8 (C1), 127.1 (C6), 117.3 (C3), 105.7 (C7), 104.3 (C2), 102.2 (C5).

IR (ATR) $\tilde{\nu}$ = 3393 (w), 1848 (w), 1714 (w), 1696 (w), 1595 (m), 1467 (m), 1436 (s), 1344 (m), 1250 (s), 1220 (m), 1164 (s), 1138 (m), 1108 (m), 1041 (s), 1005 (m), 908 (m), 843 (m), 791 (m), 760 (s), 697 (m), 675 (s).

HR-ESI-MS (Orbitrap): exact mass calculated for $[M-H]^-$ $(C_8H_4O_2Br)^-$ requires m/z 210.94002, found m/z 210.94047.

M.p.: 58.0 °C

2.2 Synthesis of *β*-Boryl Acrylates

General Procedure A: Alkyne deprotonation and nucleophilic addition to ethyl chloroformate

According to a procedure by Shindo *et al.*[10] an oven dried flask was purged with argon before the addition of alkyne (1.0 eq.) and dry THF (0.2 M). The solution was cooled to -78 °C and *n*-BuLi (1.1 eq.) was added. The solution was stirred for 1 h before the addition of ethyl chloroformate (1.1 eq.). The reaction mixture was stirred and allowed to warm to ambient temperature overnight. After subsequent quenching of the reaction by addition of sat. aq. NH_4Cl, the organics were extracted with Et_2O (3x). The combined organic layers were dried over $MgSO_4$ and the solvent was evaporated under reduced pressure. The crude residue was purified by column chromatography (SiO_2, specified combination of solvents).

Synthesis of ethyl hex-2-yonate (S18)

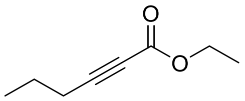

According to General Procedure **A,** pent-1-yne (197 μL, 2.0 mmol) was converted to **S18**. The reaction was run for to 2.5 h at -78 °C and the desired compound was obtained as a colourless oil (255 mg, 91%) after purification by column chromatography (5% EtOAc/*n*-pentane).

$\mathbf{R_f}$ = 0.34 (EtOAc/*n*-pentane 1:9).

^{1}**H-NMR** (300 MHz, $CDCl_3$): δ (ppm) = 4.21 (q, *J* = 7.2 Hz, 2H), 2.31 (t, *J* = 7.1 Hz, 2H), 1.61 (sextet, *J* = 7.3 Hz, 2H), 1.30 (t, *J* = 7.1 Hz, 3H), 1.01 (t, *J* = 7.4 Hz, 3H).

Analytical data in agreement with literature data.[11]

Synthesis of ethyl 3-cyclopropylpropiolate (S19)

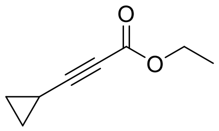

According to General Procedure **A,** ethynylcyclopropane (170 µL, 2.0 mmol) was converted to **S19** yielding a colourless oil (350 mg, 91%) after purification by column chromatography (10 % EtOAc/*n*-pentane).

$\mathbf{R_f}$ = 0.30 (EtOAc/*n*-pentane 1:9).

^{1}**H-NMR** (300 MHz, $CDCl_3$): δ (ppm) = 4.19 (q, *J* = 7.1 Hz, 2H), 1.40 – 1.34 (m, 1H), 1.28 (t, *J* = 7.1 Hz, 3H), 1.01 – 0.83 (m, 4H).

Analytical data in agreement with literature data.[12]

Synthesis of ethyl 4-cyclohexylbut-2-ynoate (S20)

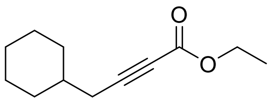

According to General Procedure **A**, prop-2-yn-1-ylcyclohexane (290 μL, 2.0 mmol) was converted to **S20** yielding a colourless oil (381 mg, 98%) after purification by column chromatography (5% EtOAc/*n*-pentane).

$\mathbf{R_f}$ = 0.63 (EtOAc/*n*-pentane 1:9).

^{1}**H-NMR** (300 MHz, $CDCl_3$): δ (ppm) = 4.21 (q, *J* = 7.1 Hz, 2H), 2.22 (d, *J* = 6.7 Hz, 2H), 1.88 – 1.49 (m, 6H), 1.30 (t, *J* = 7.2 Hz, 3H), 1.25 – 0.94 (m, 5H).

Analytical data in agreement with literature data.[13]

General Procedure B: Copper-catalyzed borylation of alkynes

According to a procedure by Santos *et al.*[14] a flask was equipped with the corresponding alkyne (1.0 eq.), $CuSO_4$ (5 mol%), 4-methylpyridine (0.25 eq.) and B_2pin_2 (0.75 eq.). Water (1.0 M) was added and the mixture was stirred at 50 °C for 10 min before the addition of the remaining B_2pin_2 (0.75 eq.). The reaction was then stirred for an additional 4-16 h before it was diluted by the addition of pentane. Organics were extracted with pentane (3x) and the combined organic layers were washed with water

and dried over $MgSO_4$. The solvent was evaporated at reduced pressure and the crude residue was purified by column chromatography (SiO_2, specified combination of solvents).

Synthesis of ethyl (*Z*)-3-(4,4,5,5-tetramethyl-1,3,2-dioxaborolan-2-yl)but-2-enoate (*Z*-2)

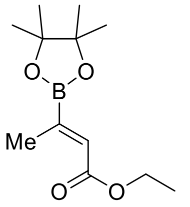

According to General Procedure **B**, ethyl but-2-ynoate (560 mg, 5.0 mmol) was converted to ***Z*-2** in 4 h and the reaction was carried out at ambient temperature. The desired product was obtained as a colourless oil (1.1 g, 92%) after purification by column chromatography (10% EtOAc/*n*-pentane).

R*f* = 0.52 (EtOAc/*n*-pentane 1:9)

^{1}H-NMR (400 MHz, $CDCl_3$): δ (ppm) = 6.44 (d, *J* = 1.8 Hz, 1H), 4.17 (q, *J* = 7.2 Hz, 2H), 2.17 (d, *J* = 1.8 Hz, 3H), 1.32 – 1.14 (m, 15H).

^{11}B NMR (128 MHz, $CDCl_3$): δ (ppm) = 29.62.

Analytical data in agreement with literature data.[14]

Synthesis of ethyl (*Z*)-3-(4,4,5,5-tetramethyl-1,3,2-dioxaborolan-2-yl)hex-2-enoate (S21)

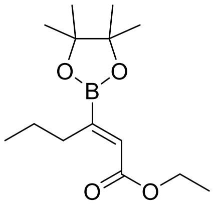

According to General Procedure **B**, **S18** (210 mg, 1.5 mmol) was converted to **S21** after 4 h. The desired product was obtained as a colourless oil (260 mg, 65%) after purification by column chromatography (10% EtOAc/*n*-pentane).

R*f* = 0.50 (EtOAc/*n*-pentane 1:9).

^{1}H-NMR (400 MHz, $CDCl_3$): δ (ppm) = 6.41 (d, *J* = 1.1 Hz, 1H), 4.16 (q, *J* = 7.1 Hz, 2H), 2.64 (ddd, *J* = 8.8, 6.4, 1.0 Hz, 2H), 1.50 – 1.38 (m, 2H), 1.27 (d, *J* = 1.9 Hz, 15H), 0.92 (t, *J* = 7.4 Hz, 3H).

^{11}B NMR (128 MHz, $CDCl_3$): δ (ppm) = 30.88.

Analytical data in agreement with literature data.[14]

Synthesis of ethyl (*Z*)-3-cyclopropyl-3-(4,4,5,5-tetramethyl-1,3,2-dioxaborolan-2-yl)acrylate (S22)

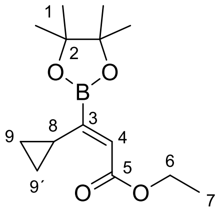

According to General Procedure **B**, **S19** (207 mg, 1.5 mmol) was converted to **S22** after 16 h. The desired product was obtained as a colourless oil (374 mg, 94%) after purification by column chromatography (10% EtOAc/*n*-pentane).

R*f* = 0.52 (EtOAc/*n*-pentane 1:9).

^{1}H-NMR (600 MHz, $CDCl_3$): δ (ppm) = 6.33 (s, 1H, H4), 4.17 (q, *J* = 7.1 Hz, 2H, H6), 3.01 (tt, *J* = 8.5, 5.1 Hz, 1H, H8), 1.27 (t, *J* = 7.1 Hz, 3H, H7), 1.22 (s, 12H, H1), 0.95 (dt, *J* = 5.8, 2.9 Hz, 2H, H9/9´), 0.84 (ddd, *J* = 8.5, 4.9, 1.6 Hz, 2H, H9/9´).

^{13}C-NMR (151 MHz, $CDCl_3$): δ (ppm) = 166.7 (C5), 127.9 (C4), 83.8 (C2), 59.7 (C6), 24.6 (C1), 14.3 (C7), 13.6 (C8), 8.5 (C9, C9´), carbon bearing boron was not observed.

^{11}B NMR (96 MHz, $CDCl_3$): δ (ppm) = 29.79.

IR (ATR) $\tilde{\nu}$ = 2980 (w), 2936 (w), 1714 (m), 1634 (m), 1445 (w), 1361 (m), 1330 (m), 1285 (s), 1265 (s), 1222 (s), 1143 (s), 1116 (m), 1098 (s), 1033 (m), 996 (m), 966 (m), 904 (w), 848 (m), 749 (m), 671 (m) cm^{-1}.

HR-ESI-MS: exact mass calculated for $[M+Na]^+$ $(C_{14}H_{23}BO_4Na)^+$ requires m/z 289.1582, found m/z 289.1595.

Synthesis of ethyl (*Z*)-4-cyclohexyl-3-(4,4,5,5-tetramethyl-1,3,2-dioxaborolan-2-yl)but-2-enoate (S23)

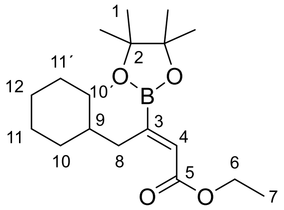

According to General Procedure **B, S20** (388 mg, 2.0 mmol) was converted to **S23** after 16 h. The desired product was obtained as a colourless oil (313 mg, 50 %) after purification by column chromatography (5% EtOAc/*n*-pentane).

R_f = 0.57 (10% EtOAc/*n*-pentane).

^{1}H NMR (500 MHz, $CDCl_3$): δ (ppm) = 6.43 (s, 1H, H4), 4.15 (q, *J* = 7.1 Hz, 2H, H6), 2.58 (dd, *J* = 7.1, 1.2 Hz, 2H, H8), 1.68 – 1.59 (m, 6H, H10/10´, H11/11´, H12), 1.53 – 147 (m, 1H, H9) 1.18 – 1.13 (m, 2H, H11/H11´) 1.27 (s, 12H, H1), 1.27 (t, *J* = 7.1 Hz, 3H, H7), 1.03 – 0.90 (m, 2H, H10/H10').

^{13}C NMR (126 MHz, $CDCl_3$): δ (ppm) = 166.2 (C5), 130.5 (C4), 84.2 (C2), 59.9 (C6), 39.0 (C9), 37.3 (C8), 33.5 (C10/C10´), 26.6 (C12), 26.6 (C11/C11´), 24.8 (C1), 14.4 (C7).

^{11}B NMR (96 MHz, $CDCl_3$): δ = 29.74 ppm.

IR (ATR) $\tilde{\nu}$ = 2979 (w), 2923 (m), 2851 (w), 1719 (m), 1448 (w), 1364 (m), 1320 (s), 1269 (w), 1251 (w), 1182 (s), 1166 (s), 1133 (s), 1029 (m), 966 (m), 893 (s), 858 (m), 696 (w), 671 (w) cm^{-1}.

HR-ESI-MS: *m*/*z*: 345.2203 ($[M+Na]^+$, calcd. for $C_{18}H_{31}BO_4Na^+$: 345.2211).

Synthesis of ethyl (*Z*)-3-phenyl-3-(4,4,5,5-tetramethyl-1,3,2-dioxaborolan-2-yl)acrylate (S24)

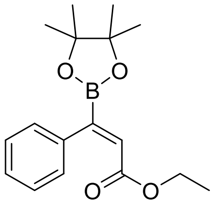

According to General Procedure **B**, ethyl 3-phenylpropiolate (495 μL, 3.0 mmol) was converted to **S24** after 16 h. The desired product was obtained as a colourless oil (743 mg, 82%) after purification by column chromatography (10% EtOAc/*n*-pentane).

R_f = 0.35 (EtOAc/*n*-pentane 1:9).

^{1}H-NMR (300 MHz, $CDCl_3$): δ (ppm) = 7.35 – 7.26 (m, 3H), 7.24 – 7.18 (m, 2H), 6.66 (s, 1H,), 4.03 (q, J = 7.1 Hz, 2H), 1.28 (s, 12H), 1.07 (t, J = 7.1 Hz, 3H).

^{11}B NMR (96 MHz, $CDCl_3$): δ (ppm) = 30.73.

Analytical data in agreement with literature data.[14]

Synthesis of (*E*)-3-Bromo-2-methylbut-2-enoic acid (S25)

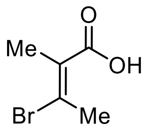

Prepared according to a modified literature procedure[15], Br_2 (6.8 mL, 132 mmol, 1.1 eq.) was added dropwise to a stirred solution of tiglic acid (12 g, 120 mmol, 1.0 eq.) in $CHCl_3$ (120 mL) at 45 ˚C under an argon atmosphere. The solution was stirred at 45 ˚C for 1.5 h. The mixture was cooled and sat. aq. $NaHCO_3$ (200 mL) was added. The organic layer was separated and washed with sat. aq. $NaHCO_3$ (2 x 100 mL). The combined aqueous layers were then acidified with 37% aq. HCl, cooled to 4 °C overnight and the solid filtered to give 2,3-dibromo-2-methylbutanoic acid which was used immediately in the subsequent step. The acid was dissolved in MeOH (24 mL) and 240 mL of a 25% KOH in methanol solution was added slowly at 0 ˚C before the addition of K_2CO_3 (4.7 g, 34.0 mmol, 0.3 eq.). The reaction was heated to 55 °C for 2 h at which point the reaction mixture was poured into a 6 M aq. HCl solution (200 mL) and diluted with H_2O (300 mL). The mixture was then cooled to 0 °C overnight and filtered to yield **S25** as a white solid (11.4 g, 53%).

^{1}H NMR (400 MHz, $CDCl_3$): δ (ppm) = 2.76 (q, *J* = 1.6 Hz, 3H), 2.12 (q, *J* = 1.6 Hz, 3H).

Analytic data in agreement with literature.[15]

Synthesis of ethyl (*E*)-3-bromo-2-methylbut-2-enoate (S26)

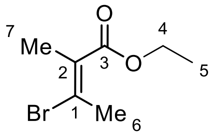

S25 (1.79 g, 10.0 mmol, 1.0 eq.) was dissolved in DMF (20 mL) under an argon atmosphere and after subsequent addition of K_2CO_3 (2.10 g, 15.0 mmol, 1.5 eq.) the mixture was stirred for 10 min. Ethyl iodide (3.12 g, 1.94 mL, 20.0 mmol, 2.0 eq.) was added and the mixture was stirred for additional 24 h at ambient temperature. The reaction was quenched by the addition of sat. aq. NH_4Cl (10 mL) and the organics were extracted with Et_2O (3 x 50 mL). The combined organic layers were dried over $MgSO_4$ and the solvent was evaporated under reduced pressure to give the desired product as a yellow oil (1.60 g, 78%).

R_f = 0.91 (EtOAc/*n*-pentane 1:9).

^{1}H NMR (400 MHz, $CDCl_3$): δ (ppm) = 4.21 (q, *J* = 7.1 Hz, 2H, H4), 2.64 (q, *J* = 1.6 Hz, 3H, H6), 2.07 (q, *J* = 1.6 Hz, 3H, H7), 1.31 (t, *J* = 7.1 Hz, 3H, H5).

^{13}C-NMR (151 MHz, CDCl3): δ (ppm) = 166.9 (C3), 135.9 (C1/C2), 128.6 (C1/C2), 61.2 (C4), 28.0 (C6), 21.1 (C7), 14.3 (C5).

IR (ATR) $\tilde{\nu}$ = 2982 (w), 2930 (w), 1713 (s), 1624 (m), 1445 (w), 1375 (w), 1365 (w), 1247 (s), 1150 (m), 1100 (s), 1057 (s), 1024 (m), 955 (w), 868 (w), 766 (m).

HR-ESI-MS (Orbitrap): exact mass calculated for $[M+Na]^+$ $(C_7H_{11}O_2BrNa)^+$ requires m/z 228.98346, found m/z 228.98356.

Synthesis of ethyl (*Z*)-2-methyl-3-(4,4,5,5-tetramethyl-1,3,2-dioxaborolan-2-yl)but-2-enoate (S27)

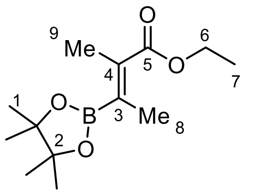

According to a procedure by Gilmour *et al*.[16], an oven dried flask was equipped with ethyl (*E*)-3-bromo-2-methylbut-2-enoate (**S26**) (623.1 mg, 3.0 mmol, 1.0 eq.), Pd(dppf)Cl_2•DCM (122.5 mg, 0.15 mmol, 5 mol%), B_2Pin_2 (835 mg, 3.3 mmol, 1.1 eq.) and KOAc (884 mg, 9.0 mmol, 3.0 eq.). The flask was sealed and purged with argon before the addition of degassed, dry 1,4-dioxane (15 mL). The reaction mixture was heated to 90 °C with stirring for 16 h. After completion, the reaction mixture was diluted with Et_2O (50 mL) and filtered off through a layer of Celite. The filtrate was washed with water (15 mL) and brine (15 mL) and the organic layer was dried over $MgSO_4$. The solvent was evaporated under reduced pressure and the crude residue was purified by column chromatography (SiO_2, 5% EtOAc/*n*-pentane) to give the desired product as a colorless oil (267 mg, 35%).

R_f = 0.69 (EtOAc/*n*-pentane 1:9).

^{1}H NMR (400 MHz, $CDCl_3$): δ (ppm) = 4.21 (q, *J* = 7.1 Hz, 2H, H6), 2.09 (q, *J* = 1.6 Hz, 3H, H9), 1.87 (q, *J* = 1.6 Hz, 3H, H8), 1.30 (t, *J* = 7.1 Hz, 3H, H7), 1.28 (s, 12H, H1).

^{13}C-NMR (151 MHz, $CDCl_3$): δ (ppm) = 170.3 (C5), 140.2 (C4), 83.7 (C2), 60.4 (C6), 24.9 (C1), 19.2 (C9), 18.4 (C8), 14.4 (C7), carbon bearing boron was not observed.

^{11}B NMR (128 MHz, $CDCl_3$): δ (ppm) = 30.04.

IR (ATR) $\tilde{\nu}$ = 2980 (w), 2933 (w), 1717 (m), 1633 (w), 1447 (w), 1356 (m), 1304 (s), 1250 (s), 1144 (s), 1083 (s), 1073 (s), 1023 (w), 972 (w), 853 (s), 773 (w), 687 (m).

HR-ESI-MS (Orbitrap): exact mass calculated for $[M+Na]^+$ $(C_{13}H_{23}BO_4Na)^+$ requires m/z 277.15841, found m/z 227.15784.

Synthesis of ethyl (*E*)-3-(4,4,5,5-tetramethyl-1,3,2-dioxaborolan-2-yl)acrylate (*E*-20)

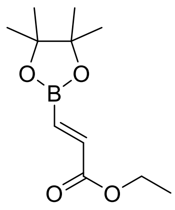

According to a procedure by Li *et al*.[17] an oven-dried flask was equipped with CuCl (24 mg, 0.24 mmol, 3 mol%), Xantphos (139 mg, 0.24 mmol, 3 mol%) and Na^{t}OBu (46 mg, 0.48 mmol, 6 mol%). The flask was purged with argon before the addition of dry THF (8 mL). The mixture was stirred for 30 min at ambient temperature. B_2Pin_2 (2.2 g, 8.8 mmol, 1.1 eq.) was added and the mixture was stirred for an additional 10 min. Ethyl propiolate (809 *μ*L, 8.0 mmol, 1.0 eq.) and MeOH (650 *μ*L, 16.0 mmol, 2.0 eq.) were added sequentially and the mixture was stirred for another 16 h at ambient temperature. After completion, the mixture was filtered over Celite and eluted with EtOAc (30 mL). The solvent was evaporated under reduced pressure and the crude residue was purified by column chromatography (SiO_2 ,10% to 20% EtOAc/*n*-pentane) to give the desired product as a colourless oil (1.35 g, 73%).

R_f = 0.59 (EtOAc/*n*-pentane 1:9).

^{1}H-NMR (400 MHz, $CDCl_3$): δ (ppm) = 6.77 (d, *J* = 18.2 Hz, 1H), 6.62 (d, *J* = 18.2 Hz, 1H), 4.21 (q, *J* = 7.1 Hz, 2H), 1.26 – 1.22 (m, 15H).

^{11}B NMR (128 MHz, $CDCl_3$): δ (ppm) = 29.90.

Analytical data in agreement with literature data.[17]

Synthesis of ethyl (*E*)-3-bromo-2-methylacrylate (S28)

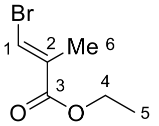

Ethyl methacrylate (630 µL, 5.0 mmol, 1.0 eq.) was dissolved in $CHCl_3$ (5 mL) and the solution was heated to 45 °C before the dropwise addition of Br_2 (257 µL, 5.0 mmol, 1.0 eq.). The reaction mixture was stirred for 1.5 h. After completion, the reaction was quenched by the addition of sat. aq. NaS_2O_3 and the organic layer was separated, washed with brine and dried over $MgSO_4$. The solvent was evaporated under reduced pressure and the solid residue was dissolved in $CHCl_3$ (5 mL) before the dropwise addition of DBU (895 µL, 6.0 mmol, 1.2 eq.). The reaction mixture was stirred for 16 h at ambient temperature before it was quenched by the addition of water (20 mL). The organics were extracted with Et_2O (3 x 15 mL) and the combined organic layers were washed with brine (20 mL) and dried over $MgSO_4$. After evaporation of the solvent the crude residue was purified by column chromatography (SiO_2, 20% to 30% DCM/*n*-pentane) to give the desired product as a yellow oil (404 mg, 42%).

R_f = 0.53 (DCM/*n*-pentane 3:7)

^{1}H-NMR (400 MHz, $CDCl_3$): δ (ppm) = 7.52 (q, *J* = 1.4 Hz, 1H, H1), 4.22 (q, *J* = 7.1 Hz, 2H, H4), 2.00 (d, *J* = 1.4 Hz, 3H, H6), 1.30 (t, *J* = 7.1 Hz, 3H, H5).

^{13}C-NMR (101 MHz, $CDCl_3$): δ (ppm) = 165.1 (C3), 134.2 (C2), 122.6 (C1), 61.2 (C4), 15.7 (C6), 14.2 (C5).

IR (ATR) $\tilde{\nu}$ = 2983 (w), 2931 (w), 1714 (s), 1676 (s), 1446 (w), 1385 (m), 1367 (m), 1304 (s), 1105 (s), 1030 (w), 989 (s), 865 (w), 726 (s), 660 (m).

HR-ESI-MS: exact mass calculated for $[M+Na]^+$ $(C_6H_9O_2BrNa)^+$ requires m/z 214.9678, found m/z 214.9668.

Synthesis of ethyl (*E*)-2-methyl-3-(4,4,5,5-tetramethyl-1,3,2-dioxaborolan-2-yl)acrylate (*E*-21)

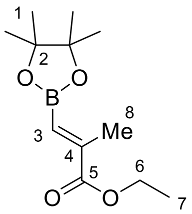

According to a procedure by Gilmour *et al*.[16], an oven dried flask was equipped with (*E*)-3-bromo-2-methylacrylate (**S28**) (388 mg, 2.0 mmol, 1.0 eq.), Pd(dppf)Cl_2•DCM (82 mg, 0.1 mmol, 5 mol%), B_2Pin_2 (557 mg, 2.2 mmol, 1.1 eq.) and KOAc (589 mg, 6.0 mmol, 3.0 eq.). The flask was sealed and purged with argon before the addition of degassed, dry 1,4-dioxane (10 mL). The reaction mixture was heated to 90 °C with stirring for 16 h. The reaction mixture was diluted with Et_2O (50 mL) and filtered off through a layer of Celite. The filtrated was washed with water (10 mL) and brine (10 mL) and the organic layer was dried over $MgSO_4$. The solvent was evaporated under reduced pressure and the crude residue was purified

by column chromatography (SiO_2, 5% EtOAc/*n*-pentane) to give the desired product as a colorless oil (150 mg, 39%).

R*f* = 0.67 (EtOAc/*n*-pentane 1:9).

^{1}H-NMR (600 MHz, $CDCl_3$): δ (ppm) = 6.52 (q, *J* = 1.2 Hz, 1H, H3), 4.19 (q, *J* = 7.1 Hz, 2H, H6), 2.14 (d, *J* = 1.2 Hz, 3H, H8), 1.28 (s, 12H, H1), 1.28 (t, *J* = 7.1 Hz, 3H, H7).

^{13}C-NMR (151 MHz, $CDCl_3$): δ (ppm) = 167.8 (C5), 147.4 (C4), 83.5 (C2), 60.8 (C6), 24.8 (C1), 17.0 (C8), 14.1 (C7), carbon bearing boron was not observed.

^{11}B NMR (128 MHz, $CDCl_3$): δ (ppm) = 30.27.

IR (ATR) $\tilde{\nu}$ = 2980 (w), 2935 (w), 1714 (m), 1634 (m), 1445 (w), 1361 (m), 1330 (m), 1285 (s), 1265 (s), 1222 (s), 1143 (s), 1116 (m), 1098 (s), 966 (m), 905 (w), 848 (m), 749 (m), 671 (m) cm^{-1}.

HR-ESI-MS: exact mass calculated for $[M+Na]^+$ $(C_{12}H_{21}BO_4Na)^+$ requires m/z 263.1425, found m/z 263.1446.

2.3 Photocatalyzed Isomerization of *β*-Boryl Acrylates

General Procedure C: Photocatalytic Isomeristion of *β*-Boryl Acrylates

A flask was equipped with the corresponding acrylate (0.3 mmol, 1.0 eq.) and thioxanthone (3 mg, 5 mol%). The flask was sealed and purged with argon before the addition of dry, degassed MeCN (0.03 M, 9 mL). The reaction mixture was stirred for 1 h at ambient temperature under the irradiation of violet light (*λ* = 402 nm). After completion, solvent was evaporated under reduced pressure and the crude residue was purified by column chromatography (SiO_2, specified combination of solvents).

Synthesis of ethyl (*Z*)-3-(4,4,5,5-tetramethyl-1,3,2-dioxaborolan-2-yl)acrylate (*Z*-20)

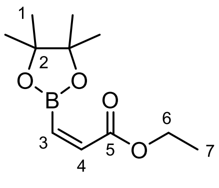

According to General Procedure **C**, ethyl (*E*)-3-(4,4,5,5-tetramethyl-1,3,2-dioxaborolan-2-yl)acrylate (***E*-20**, 68 mg, 0.3 mmol) was converted to ***Z*-20** yielding a colorless oil (46 mg, 66%, *E*/*Z* 6:94) after purification by column chromatography (0% to 15% EtOAc/DCM).

R*f* = 0.83 (EtOAc/DCM 1:9)

^{1}H-NMR (500 MHz, $CDCl_3$): δ (ppm) = 6.33 (d, *J* = 13.4 Hz, 1H, H3), 6.26 (d, *J* = 13.3 Hz, 1H, H4), 4.21 (q, *J* = 7.1 Hz, 2H, H6), 1.35 (s, 12H, H1), 1.28 (t, *J* = 7.1 Hz, 3H, H7).

^{13}C-NMR (126 MHz, $CDCl_3$): δ (ppm) = 167.1 (C5), 133.3 (C4), 84.0 (C2), 60.6 (C6), 24.8 (C1), 14.2 (C7), carbon bearing boron was not observed.

^{11}B NMR (96 MHz, $CDCl_3$): δ (ppm) = 29.81.

IR (ATR) $\tilde{\nu}$ = 2979 (w), 2934 (w), 1719 (m), 1627 (m), 1466 (w), 1412 (m), 1371 (m), 1312 (s), 1267 (m), 1204 (s), 1140 (s), 1030 (m), 968 (s), 846 (m), 757 (s), 670 (m) cm^{-1}.

HR-ESI-MS exact mass calculated for $[M+Na]^+$ $(C_{11}H_{19}BO_4Na)^+$ requires m/z 249.1269, found m/z 249.1294.

Synthesis of ethyl (*Z*)-2-methyl-3-(4,4,5,5-tetramethyl-1,3,2-dioxaborolan-2-yl)acrylate (*Z*-21)

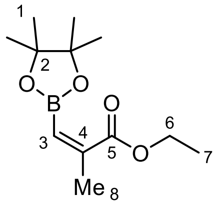

According to General Procedure **C**, (*E*)-2-methyl-3-(4,4,5,5-tetramethyl-1,3,2-dioxaborolan-2-yl)acrylate (***E*-21** 72 mg, 0.3 mmol) was converted to ***Z*-21** yielding a colourless oil (35 mg, 49%, *E*/*Z* 8:92) after purification by column chromatography (DCM).

R*f* = 0.54 (DCM)

^{1}H-NMR (600 MHz, $CDCl_3$): δ (ppm) = 5.90 (q, *J* = 1.5 Hz, 1H, H3), 4.21 (q, *J* = 7.1 Hz, 2H, H6), 1.97 (d, *J* = 1.5 Hz, 3H, H8), 1.32 (s, 12H, H1), 1.28 (t, *J* =7.1 Hz, 3H, H7).

^{13}C NMR (151 MHz, $CDCl_3$): δ (ppm) = 168.4 (C5), 142.2 (C4),83.6 (C2), 61.0 (C6), 24.8 (C1), 20.4 (C8), 14.2 (C7), carbon bearing boron was not observed.

^{11}B-NMR (192 MHz, $CDCl_3$): δ (ppm) = 29.90.

IR (ATR) $\tilde{\nu}$ = 2979 (w), 2931 (w), 1713 (m), 1641 (w), 1449 (w), 1370 (m), 1356 (m), 1302 (s), 1199 (s), 1141 (s), 1113 (s), 1017 (m), 968 (m), 849 (m), 760 (w), 720 (m) cm^{-1}.

HR-ESI-MS: exact mass calculated for $[M+Na]^+$ $(C_{12}H_{21}BO_4Na)^+$ requires m/z 263.1425, found m/z 263.1446.

2.4 Synthesis of Coumarin Derivatives

General Procedure D: Synthesis of Coumarin Derivatives

A Schlenk-tube, equipped with the respective 2-bromophenol (0.25 mmol, 1.0 eq.), *β*-boryl acrylate (0.5 mmol, 2.0 eq.), $Pd(OAc)_2$ (2.9 mg, 0.013 mmol, 5 mol%), SPhos (10.3 mg, 0.025 mmol, 10 mol%) and K_3PO_4 (159.2 mg, 0.75 mmol, 3.0 eq.) was sealed and purged with argon before the addition of dry DMF (1 mL, 0.25 M). The reaction mixture was stirred at 80 °C for 16 h. After cooling to ambient temperature the mixture was filtered through a plug of Celite and eluted with EtOAc. The solvent was evaporated under reduced pressure and the crude mixture was purified by column chromatography (SiO_2, specified combination of solvents).

Synthesis of 4-methyl-2*H*-chromen-2-one (3)

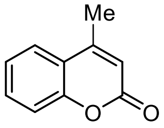

According to General Procedure **D**, ***Z*-2** (120.1 mg) and 2-bromophenol (29 *µ*L, 43.4 mg) were converted to 4-methyl-2*H*-chromen-2-one (**3**) yielding a colourless solid (30.5 mg, 76%) after purification by column chromatography (10% EtOAc/*n*-pentane).

R*f* = 0.19 (EtOAc/*n*-pentane 1:9)

^{1}H-NMR (300 MHz, $CDCl_3$): δ (ppm) = 7.61 (dd, *J* = 7.9, 1.6 Hz, 1H), 7.54 (ddd, *J* = 8.6, 7.2, 1.6 Hz, 1H), 7.35 – 7.28 (m, 2H), 6.30 (d, *J* = 1.3 Hz, 1H), 2.45 (d, *J* = 1.3 Hz, 3H).

Analytical data in agreement with literature data.[18]

Synthesis of 4-propyl-2*H*-chromen-2-one (4)

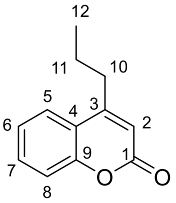

According to General Procedure **D**, **S21** (134.1 mg) and 2-bromophenol (29 *µ*L, 43.4 mg) were converted to 4-propyl-2*H*-chromen-2-one (**4**) yielding a colourless solid (32.4 mg, 69%) after purification by column chromatography (10% EtOAc/ *n*-pentane).

R*f* = 0.28 (EtOAc/*n*-pentane 1:9).

^{1}H-NMR (600 MHz, $CDCl_3$): δ (ppm) = 7.64 (dd, *J* = 8.0, 1.5 Hz, 1H, H5), 7.52 (ddd, *J* = 8.5, 7.2, 1.6 Hz, 1H, H7), 7.37 – 7.23 (m, 2H, H6, H8), 6.29 (d, *J* = 1.2 Hz, 1H, H2), 2.75 (t, *J* = 7.6 Hz, 2H, H10), 1.75 (h, *J* = 7.4 Hz, 2H, H11), 1.06 (t, *J* = 7.3 Hz, 3H, H12).

^{13}C-NMR (151 MHz, $CDCl_3$): δ (ppm) = 161.0 (C1), 156.0 (C3), 153.7 (C9), 131.5 (C7), 124.3 (C5), 124.1 (C6), 119.3 (C4) 117.3 (C8), 114.0 (C2), 33.7 (C10), 21.3 (C11), 13.9 (C12).

IR (ATR) $\tilde{\nu}$ = 3059 (w), 2920 (m), 2835 (m), 1702 (s), 1574 (m), 1492 (m), 1424 (m), 1385 (m), 1367 (w), 1279 (m), 1262 (m), 1168 (m), 1032 (m), 913 (m), 872 (m), 838 (s), 745 (m), 710 (m) cm^{-1}.

HR-ESI-MS exact mass calculated for $[M+Na]^+$ $(C_{12}H_{12}O_2Na)^+$ requires m/z 211.0730, found m/z 211.0721.

M.p.: 75.1– 77.0 °C.

Synthesis of 4-cyclopropyl-2*H*-chromen-2-one (5)

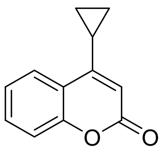

According to General Procedure **D**, **S22** (133.1 mg) and 2-bromophenol (29 *µ*L, 43.4 mg) were converted to 4-cyclopropyl-2*H*-chromen-2-one (**5**) yielding a colourless solid (31.0 mg, 67%) after purification by column chromatography (10% EtOAc/cyclohexane).

R*f* = 0.17 (EtOAc/*n*-pentane 1:9)

^{1}H-NMR (300 MHz, $CDCl_3$): δ (ppm) = 7.92 (dd, *J* = 8.4, 1.6 Hz, 1H), 7.53 (ddd, *J* = 8.4, 7.3, 1.6 Hz, 1H), 7.39 – 7.22 (m, 2H), 6.05 (d, *J* = 1.2 Hz, 1H), 2.11 (dtt, *J* = 8.5, 6.5, 4.7 Hz, 1H), 1.21 – 1.06 (m, 2H), 0.88 – 0.78 (m, 2H).

Analytical data in agreement with literature data.[19]

Synthesis of 4-(cyclohexylmethyl)-2*H*-chromen-2-one (6)

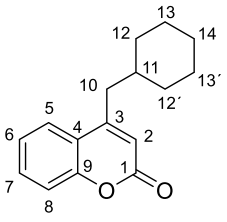

According to General Procedure **D**, **S23** (161.5 mg) and 2-bromophenol (29 *μ*L, 43.4 mg) were converted to 4-(cyclohexylmethyl)-2*H*-chromen-2-one (**6**) yielding a colourless solid (45.4 mg, 75%) after purification by column chromatography (5% EtOAc/*n*-pentane).

R*f* = 0.41 (EtOAc/*n*-pentane 1:9)

^{1}H-NMR (500 MHz, $CDCl_3$): δ (ppm) = 7.62 (dd, *J* = 8.0, 1.6 Hz, 1H, H5), 7.52 (ddd, *J* = 8.6, 7.3, 1.5 Hz, 1H, H7), 7.34 (dd, *J* = 8.4, 1.3 Hz, 1H, H8), 7.29 (ddd, *J* = 8.1, 7.3, 1.2 Hz, 1H, H6), 6.23 (d, *J* = 0.8 Hz, 1H, H2), 2.63 (dd, *J* = 7.0, 0.8 Hz, 2H, H10), 1.83 – 1.56 (m, 7H, H11, H12/H12´, H13/H13, H14).1.28 – 1.13 (m, 2H, H13/H13´), 1.04 (qd, *J* = 12.0, 11.6, 3.1 Hz, 2H, H12/H12´).

^{13}C-NMR (126 MHz, $CDCl_3$): δ (ppm) = 161.0 (C1), 155.0 (C3), 154.0 (C9), 131.7 (C7), 124.8 (C5), 124.2 (C6), 119.7 (C4), 117.5 (C8), 115.2 (C2), 40.0 (C10), 37.3 (C11), 33.6 (C12), 26.3 (C14), 26.2 (C13).

IR (ATR) $\tilde{\nu}$ = 2919 (m), 2850 (m), 1724 (s), 1620 (w), 1603 (m), 1564 (w), 1448 (m), 1386 (w), 1255 (m), 1222 (w), 1180 (s), 1125 (w), 1038 (w), 925 (s), 881 (s), 855 (m), 767 (w), 715 (s) cm^{-1}.

HR-ESI-MS exact mass calculated for $[M+Na]^+$ $(C_{16}H_{18}O_2Na)^+$ requires m/z 265.1199, found m/z 265.1225.

M.p.: 100.3 – 102.3 °C.

Synthesis of 4-(cyclohexylmethyl)-2*H*-chromen-2-one (7)

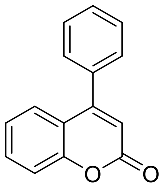

According to General Procedure **D**, **S24** (151.0 mg) and 2-bromophenol (29 *μ*L, 43.4 mg) were converted to 4-phenyl-2*H*-chromen-2-one (**7**) yielding a colourless solid (40.5 mg, 73%) after purification by column chromatography (10% EtOAc/cyclohexane).

R*f* = 0.28 (EtOAc/*n*-pentane 1:9)

^{1}H-NMR (300 MHz, $CDCl_3$): δ (ppm) = 7.57 – 7.50 (m, 5H), 7.49 – 7.44 (m, 2H), 7.43 (d, *J* = 1.2 Hz, 1H), 7.25 – 7.20 (m, 1H), 6.39 (s, 1H).

Analytical data in agreement with literature data.[20]

Synthesis of 3,4-dimethyl-2*H*-chromen-2-one (8)

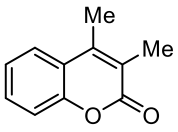

According to General Procedure **D**, **S27** (127.0 mg) and 2-bromophenol (29 *μ*L, 43.4 mg) were converted to 3,4-dimethyl-2*H*-chromen-2-one (**8**) yielding a colourless solid (36.0 mg, 83%) after purification by column chromatography (0% to 10% EtOAc/*n*-pentane).

R*f* = 0.47 (EtOAc/*n*-pentane 1:9)

^{1}H-NMR (300 MHz, $CDCl_3$): δ (ppm) = 7.61 (dd, *J* = 7.9, 1.6 Hz, 1H), 7.47 (ddd, *J* = 8.5, 7.1, 1.5 Hz, 1H), 7.36 – 7.22 (m, 2H), 2.42 (d, *J* = 0.9 Hz, 3H), 2.23 (d, *J* = 0.9 Hz, 3H)

Analytical data in agreement with literature data.[21]

Synthesis of 7-fluoro-4-methyl -2*H*-chromen-2-one (9)

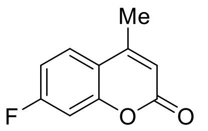

According to General Procedure **D**, ***Z*-2** (120.1 mg) and 2-bromo-5-fluorophenol (28 *µ*L, 48 mg) were converted to 7-fluoro-4-methyl-2*H*-chromen-2-one (**9**) yielding a colourless solid (34 mg, 76%) after purification by column chromatography (15% EtOAc/*n*-pentane).

R*f* = 0.19 (EtOAc/*n*-pentane 1:9)

^{1}H-NMR (400 MHz, $CDCl_3$): δ (ppm) = 7.58 (dd, *J* = 9.5, 6.0 Hz, 1H), 7.09 – 6.97 (m, 2H), 6.23 (s, 1H), 2.42 (d, *J* = 1.3 Hz, 3H).

^{19}F-NMR (376 MHz, $CDCl_3$): δ (ppm) = -105.87.

Analytical data in agreement with literature data.[22]

Synthesis of 7-amino-4-methyl-2*H*-chromen-2-one (10)

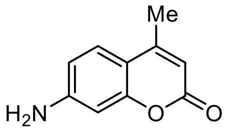

According to General Procedure **D**, ***Z*-2** (120.1 mg) and 2-bromo-5-aminophenol (**1**, 46.5 mg) were converted to 7-amino-4-methyl-2*H*-chromen-2-one (**10**) yielding a yellow solid (31.0 mg, 71%) after purification by column chromatography (50% EtOAc/*n*-pentane).

R*f* = 0.5 (EtOAc/*n*-pentane 1:1)

^{1}H-NMR (300 MHz, DMSO-d_6): δ (ppm) = 7.40 (d, *J* = 8.6 Hz, 1H), 6.55 (dd, *J* = 8.6, 2.2 Hz, 1H), 6.40 (d, *J* = 2.2 Hz, 1H), 6.11 (br s, 2H), 5.90 (d, *J* = 1.1 Hz, 1H), 2.29 (d, *J* = 1.1 Hz, 3H).

Analytical data in agreement with literature data.[23]

Synthesis of 6-methoxy-4-methyl-2*H*-chromen-2-one (11)

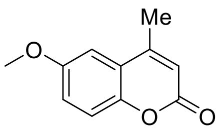

According to General Procedure **D**, ***Z*-2** (120.1 mg) and 2-bromo-4-metoxyphenol (**2**, 51 mg) were converted to 7-methoxy-4-methyl-2*H*-chromen-2-one (**11**) yielding a colourless solid (30.0 mg, 63%) after purification by column chromatography (5% EtOAc/*n*-pentane).

R*f* = 0.12 (EtOAc/*n*-pentane 1:9)

^{1}H-NMR (600 MHz, $CDCl_3$): δ (ppm) = 7.27 (d, *J* = 10.1 Hz, 1H, H5), 7.10 (ddd, *J* = 9.0, 3.0, 1.2 Hz, 1H, H6), 7.02 (dd, *J* = 3.0, 1.2 Hz, 1H, H8), 6.30 (s, 1H, H2), 3.86 (d, *J* = 0.9 Hz, 3H, H10), 2.42 (s, 3H, H11).

Analytical data in agreement with literature.[24]

Synthesis of 6-chloro-4-methyl-2*H*-chromen-2-one (12)

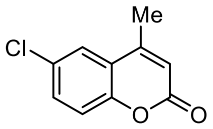

According to General Procedure **D**, ***Z*-2** (120.1 mg) and 2-bromo-4-chlorophenol (51.8 mg) were converted to 6-chloro-4-methyl-2*H*-chromen-2-one (**12**) yielding a colourless solid (31.0 mg, 63%) after purification by column chromatography (10% to 20% EtOAc/*n*-pentane).

R_f = 0.45 (EtOAc/*n*-pentane 1:9)

^{1}H-NMR (300 MHz, $CDCl_3$): δ (ppm) = 7.57 (d, *J* = 2.4 Hz, 1H), 7.48 (dd, *J* = 8.8, 2.4 Hz, 1H), 7.28 (d, *J* = 8.8 Hz, 1H), 6.33 (d, *J* = 1.3 Hz, 1H), 2.43 (d, *J* = 1.3 Hz, 3H).

Analytical data in agreement with literature data.[22]

Synthesis of 4,8-dimethyl-2*H*-chromen-2-one (13)

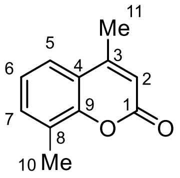

According to General Procedure **D**, ***Z*-2** (120.1 mg) and 2-bromo-6-methylphenol (31 *µ*L, 46.8 mg) were converted to 4,8-dimethyl-2*H*-chromen-2-one (37 mg, 84%). Yielding a colourless solid after purification by column chromatography (10% EtOAc/*n*-pentane).

R_f = 0.30 (EtOAc/*n*-pentane 1:9)

^{1}H-NMR (599 MHz, $CDCl_3$): δ (ppm) = 7.44 (dd, *J* = 7.9, 1.6 Hz, 1H, H5), 7.38 (dd, *J* = 7.4, 0.8 Hz, 1H, H7), 7.19 (t, *J* = 7.7 Hz, 1H, H6), 6.28 (d, *J* = 1.3 Hz, 1H, H2), 2.45 (s, 3H, H10), 2.43 (d, *J* = 1.3 Hz, 3H, H11).

^{13}C-NMR (151 MHz, $CDCl_3$): δ (ppm) = 161.1 (C1), 152.8 (C3), 152.0 (C9), 133.2 (C7), 126.6 (C8), 123.8 (C6), 122.3 (C5), 119.8 (C4), 115.0 (C2), 19.0 (C11), 15.8 (C10).

IR (ATR) ṽ = 3053 (w), 2985 (w), 1702 (s), 1598 (s), 1580 (m), 1457 (m), 1431 (m), 1388 (m), 1365 (m), 1249 (m), 1172 (m), 1132 (m), 1096 (w), 1081 (w), 1042 (w), 998 (w), 938 (m), 880 (s), 832 (m), 789 (m), 748 (s), 733 (m) cm^{-1}.

HR-EI-MS (Orbitrap): exact mass calculated for [M] ($C_{11}H_{10}O_2$) requires m/z 174.06753, found m/z 174.06746.

M.p.: 114.1 – 115.6 °C

Synthesis of 1-methyl-3*H*-benzo[*f*]chromen-3-one (14)

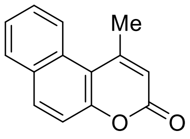

According to General Procedure **D**, ***Z*-2** (120.1 mg) and 1-bromonaphthalen-2-ol (56 mg) were converted to 1-methyl-3*H*-benzo[*f*]chromen-3-one (**14**) yielding a colourless solid (42.0 mg, 80%) after purification by column chromatography (5% EtOAc/*n*-pentane).

R_f = 0.15 (EtOAc/*n*-pentane 1:9).

^{1}H-NMR (300 MHz, $CDCl_3$): δ (ppm) = 8.61 (d, *J* = 8.7 Hz, 1H), 7.98 (d, *J* = 9.0 Hz, 1H), 7.93 (d, *J* = 8.0 Hz, 1H), 7.65 (ddd, *J* = 8.7, 6.8, 1.6 Hz, 1H), 7.56 (t, *J* = 7.2 Hz, 1H), 7.48 (d, *J* = 8.9 Hz, 1H), 6.40 (d, *J* = 1.4 Hz, 1H), 2.95 (s, 3H).

Analytical data in agreement with literature data.[25]

Synthesis of 4-methyl-6-(naphthalen-1-yl)-2*H*-chromen-2-one (15)

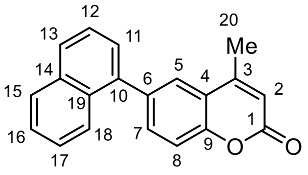

According to General Procedure **D**, ***Z*-2** (120.1 mg) and **S4** (74.5 mg) were converted to 4-methyl-6-(naphthalen-1-yl)-2*H*-chromen-2-one (**15**) yielding a colourless solid (48.5 mg, 68%) after purification by column chromatography (2% EtOAc/*n*-pentane).

R_f = 0.42 (EtOAc/*n*-pentane 1:9)

^{1}H-NMR (500 MHz, $CDCl_3$): δ (ppm) = 7.94 (d, J = 8.2 Hz, 1H, H15), 7.91 (d, J = 8.3 Hz, 1H, H13), 7.81 (dd, J = 8.4, 1.1 Hz, 1H, H18), 7.71 (d, J = 2.0 Hz, 1H, H5), 7.66 (dd, J = 8.4, 2.1 Hz, 1H, H7), 7.59 – 7.50 (m, 2H, H12, H16), 7.49 – 7.42 (m, 3H, H8, H11, H17), 6.36 (d, J = 1.3 Hz, 1H, H2), 2.44 (d, J = 1.3 Hz, 3H, H20).

^{13}C-NMR (126 MHz, $CDCl_3$): δ (ppm) = 160.9 (C1), 153.0 (C9), 152.5 (C3), 138.7 (C10), 137.1 (C6), 133.9 (C14), 133.6 (C7), 131.7 (C19), 128.6 (C15), 128.4 (C13), 127.3 (C11), 126.6 (C17), 126.2 (C16), 125.9(C5), 125.5 (C12), 125.5 (C18), 120.0 (C4), 117.0 (C8), 115.6 (C2), 18.8 (C20).

IR (ATR) ṽ = 3057 (w), 2922 (w), 1725 (s), 1713 (s), 1626 (m), 1607 (m), 1568 (m), 1506 (w), 1486 (w), 1419 (w), 1390 (m), 1374 (m), 1333 (w), 1271 (m), 1223 (m), 1186 (m), 1175 (m), 1066 (m), 1019 (w), 918 (s), 891 (m), 874 (m), 836 (m), 802 (s), 792 (s), 776 (s), 658 (m) cm^{-1}.

HR-ESI-MS: exact mass calculated for $[M+Na]^+$ $(C_{20}H_{14}O_2Na)^+$ requires m/z 309.0886, found m/z 309.0885.

M.p.: 129.2 – 130.5 °C

Synthesis of methyl (*S*)-2-((*tert*-butoxycarbonyl)amino)-3-(4-methyl-2-oxo-2*H*-chromen-6-yl)propanoate (16)

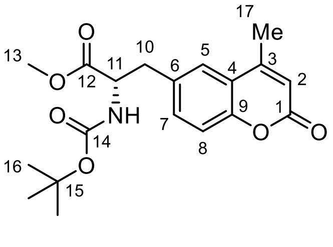

According to General Procedure **D**, ***Z*-2** (120.1 mg) and **S5** (94 mg) were converted to methyl (*S*)-2-((*tert*-butoxycarbonyl)amino)-3-(4-methyl-2-oxo-2*H*-chromen-6-yl)propanoate (**16**) yielding a colourless solid (73.1 mg, 81%) after purification by column chromatography (10% to 30% EtOAc/*n*-pentane).

R_f = 0.25 (EtOAc/*n*-pentane 3:7)

^{1}H-NMR (599 MHz, $CDCl_3$): δ (ppm) = 7.35 (d, J = 2.0 Hz, 1H, H5), 7.29 (dd, J = 8.4, 2.0 Hz, 1H, H7), 7.25 (d, J = 8.4 Hz, 1H, H8), 6.29 (d, J = 1.3 Hz, 1H, H2), 5.08 – 5.01 (m, 1H, NH), 4.61 (q, J = 6.8 Hz, 1H, H11), 3.73 (s, 3H, H13), 3.22 (dd, J = 14.1, 5.8 Hz, 1H, $H10_A$), 3.08 (dd, J = 14.2, 6.4 Hz, 1H, $H10_B$), 2.42 (d, J = 1.2 Hz, 3H, H17), 1.40 (s, 9H, H16).

^{13}C-NMR (151 MHz, $CDCl_3$): δ (ppm) = 172.1 (C12), 160.8 (C1), 155.1 (C14), 152.7 (C9), 152.1 (C3), 132.9 (C6), 132.4 (C7), 125.3 (C5), 120.0 (C4), 117.3 (C8), 115.5 (C2), 80.3 (C15), 54.5 (C11), 52.5 (C13), 38.0 (C10), 28.4 (C16), 18.7 (C17).

IR (ATR) $\tilde{\nu}$ = 3363 (w), 2971 (w), 2922 (w), 1727 (s), 1696 (s), 1628 (w), 1572 (w), 1502 (w), 1430 (m), 1365 (m), 1256 (m), 1223 (m), 1172 (s), 1071 (m), 1023 (m), 929 (m), 920 (m), 851 (m), 830 (m), 787 (m), 763 (w) cm^{-1}.

HR-ESI-MS: exact mass calculated for $[M+Na]^+$ $(C_{19}H_{23}NO_6Na)^+$ requires m/z 384.1418, found m/z 384.1416.

ORD ($CHCl_3$, c 1.00): $[\alpha]_D^{23}$= 3.022.

M.p.: 152.3 – 155.7 °C

Synthesis of (6b*S*,8a*S*,11a*S*,11b*R*)-1,8a-dimethyl-6b,7,8,8a,10,11,11a,11b,12,13-decahydrocyclo-penta[5,6]naphtho[2,1-*f*]chromene-3,9-dione (17)

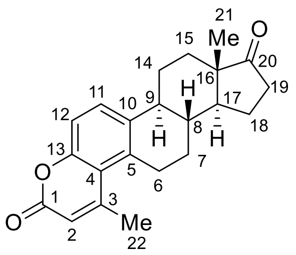

According to General Procedure **D**, ***Z*-2** (120.1 mg) and **S7** (87 mg) were converted to **17** yielding a colourless solid (42.0 mg, 50%) after purification by column chromatography (10% to 30% EtOAc/*n*-pentane).

R_f = 0.18 (EtOAc/*n*-pentane 3:7).

^{1}H-NMR (599 MHz, $CDCl_3$): δ (ppm) = 7.48 (d, *J* = 8.7 Hz, 1H, H11), 7.15 (d, *J* = 8.7 Hz, 1H, H12), 6.20 (d, *J* = 1.3 Hz, 1H, H2), 3.33 – 3.21 (m, 2H, H6), 2.64 (d, *J* = 1.2 Hz, 3H, H22), 2.51 (dd, *J* = 19.1, 8.8 Hz, 1H, $H19_A$), 2.43 – 2.32 (m, 2H, H9, $H14_A$), 2.18 – 2.04 (m, 3H, $H7_A$, $H18_A$, $H19_B$), 2.00 – 1.93 (m, 1H, $H15_A$), 1.68 – 1.55 (m, 2H, H8, $H18_B$), 1.56 – 1.48 (m, 3H, $H14_B$ $H15_B$, H17), 1.44 – 1.34 (m, 1H, $H7_B$), 0.92 (s, 3H, H21).

^{13}C-NMR (151 MHz, $CDCl_3$): δ (ppm) = 220.2 (C20), 160.4 (C1), 154.2 (C3), 153.3 (C13), 136.8 (C4), 135.9 (C5), 129.4 (C11), 119.0 (C10), 117.3 (C2), 115.6 (C12), 50.2 (C17), 47.8 (C16), 45.1 (C9), 37.0 (C8), 35.8 (C19), 31.7 (C15), 30.4 (C6), 26.7 (C22), 26.6 (C14), 26.4 (C7), 21.4 (C18), 13.8 (C21).

IR (ATR) $\tilde{\nu}$ = 3080 (w), 2924 (w), 2860 (w), 2363 (w), 1733 (s), 1716 (s), 1607 (w), 1587 (m), 1565 (m), 1466 (m), 1383 (m), 1325 (w), 1240 (m), 1206 (m), 1171 (m), 1066 (m), 1038 (m), 1014 (m), 964 (m), 841 (s), 826 (m), 696 (m) 663 (w) cm^{-1}.

HR-ESI-MS (Orbitrap): exact mass calculated for $[M+Na]^+$ $(C_{22}H_{24}O_3Na)^+$ requires m/z 359.16177, found m/z 359.16163.

ORD ($CHCl_3$, c 1.00): $[\alpha]_D^{23}$= 193.211.

M.p.: 237.5 – 240.1 °C

Synthesis of (3a*S*,3b*R*,11b*S*,13a*S*)-10,13a-dimethyl-2,3,3a,3b,4,5,11b,12,13,13a-decahydrocyclo-penta[5,6]naphtho[1,2-*g*]chromene-1,8-dione (18)

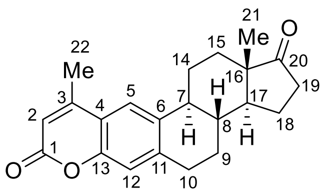

According to General Procedure **D**, ***Z*-2** (91.2 mg) and **S6** (67.6 mg, 0.19 mmol) were converted to **18** yielding a yellow solid (35.0 mg, 55%) after purification by column chromatography (10% to 30% EtOAc/*n*-pentane).

$\mathbf{R_f}$ = 0.35 (EtOAc/*n*-pentane 3:7).

^{1}H-NMR (599 MHz, $CDCl_3$): δ (ppm) = 7.46 (s, 1H, H5), 7.04 (s, 1H, H12), 6.20 (d, *J* = 1.3 Hz, 1H, H2), 3.04 – 2.93 (m, 2H, H10), 2.52 (dd, *J* = 19.1, 8.9 Hz, 1H, $H19_A$), 2.50 – 2.44 (m, 1H, $H9_A$), 2.41 (d, *J* = 1.3 Hz, 3H, H22), 2.33 (td, *J* = 11.1, 4.4 Hz, 1H, H7), 2.20 – 2.11 (m, 1H, $H19_B$), 2.11 – 2.02 (m, 2H, $H14_A$, $H18_A$), 2.01 (dt, *J* = 12.9, 2.9 Hz, 1H, $H15_A$), 1.71 – 1.43 (m, 7H, H8, $H9_B$, $H14_B$, $H15_B$, H17, $H18_B$), 0.92 (s, 3H, H21).

^{13}C-NMR (151 MHz, $CDCl_3$): δ (ppm) = 220.5 (C20), 161.3 (C1), 152.6 (C3), 151.8 (C13), 141.8 (C6), 136.4 (C11), 121.1 (C5), 117.9 (C4), 116.7 (C12), 114.3 (C2), 50.6 (C17), 48.0 (C16), 44.1 (C7), 38.1 (C8), 35.9 (C19), 31.6 (C15), 29.6 (C10), 26.2 (C14), 26.1 (C9), 21.7 (C18), 18.7 (C22), 13.9 (C21).

IR (ATR) $\tilde{\nu}$ = 2909 (m), 2851 (m), 1716 (s), 1671 (m), 1558 (m), 1456 (w), 1417 (m), 1380 (m), 1282 (w), 1259 (m), 1197 (m), 1153 (m), 1086 (m), 1058 (m), 1037 (s), 1014 (m), 961 (m), 932 (m), 883 (m), 844 (s), 744 (w), 704 (w), 690 (w) cm^{-1}.

HR-ESI-MS: exact mass calculated for $[M+Na]^+$ $(C_{22}H_{24}O_3Na)^+$ requires m/z 359.1618, found m/z 359.1616.

ORD ($CHCl_3$, c 1.00): $[\alpha]_D^{23}$= 125.603.

M.p.: 220.5 – 221.5 °C

Synthesis of (*Z*)-8-methoxy-4-methyl-5-(3,4,5-trimethoxystyryl)-*2H*-chromen-2-one (19)

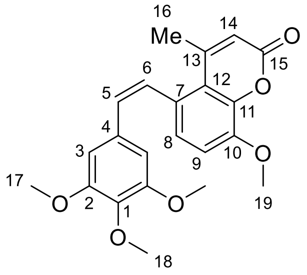

According to General Procedure **D**, ***Z*-2** (120.1 mg) and **S14** (98.8 mg) were converted to **19** yielding a yellow solid (56.1 mg, 59%) after purification by column chromatography (10% to 30% EtOAc/*n*-pentane).

$\mathbf{R_f}$ = 0.16 (EtOAc/*n*-pentane 3:7).

^{1}H-NMR (500 MHz, $CDCl_3$): δ (ppm) = 7.02 (d, *J* = 8.6 Hz, 1H, H8), 6.99 (d, *J* = 8.6 Hz, 1H. H9), 6.89 (d, *J* = 12.0 Hz, 1H, H6), 6.54 (d, *J* = 12.0 Hz, 1H, H5), 6.28 (d, *J* = 1.3 Hz, 1H, H14), 6.25 (s, 2H, H3), 3.93 (s, 3H, H19), 3.78 (s, 3H, H18), 3.55 (s, 6H, H17), 2.61 (d, *J* = 1.3 Hz, 3H, H16).

^{13}C-NMR (126 MHz, $CDCl_3$): δ (ppm) = 159.7 (C15), 154.2 (C12) 153.0 (C2), 147.0 (C10), 144.7 (C11), 137.7 (C1), 131.5 (C4), 131.0 (C5), 130.3 (C6), 128.8 (C7), 126.8 (C8), 119.3 (C13), 116.9 (C14), 113.5 (C9), 106.6 (C3), 61.0 (C18), 56.5 (C19), 55.9 (C17), 24.64 (C16).

IR (ATR) $\tilde{\nu}$ = 3385 (w), 3004 (w), 2967 (w), 2839 (w), 1582 (m), 1508 (m), 1489 (m), 1421 (m), 1333 (m), 1278 (m), 1238 (s), 1187 (m), 1125 (s), 1031 (s), 996 (s), 860 (m), 821 (m), 803 (m), 706 (m), 658 (m) cm^{-1}.

HR-ESI-MS (Orbitrap): exact mass calculated for $[M+Na]^+$ $(C_{22}H_{22}O_6Na)^+$ requires m/z 405.13086, found m/z 405.13076.

M.p.: 134.4 – 137.0 °C.

Synthesis of ethyl (*E*)-3-(2-hydroxyphenyl)acrylate (22)

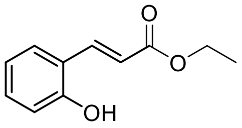

According to General Procedure **D**, ***E*-20** (113.5 mg) and 2-bromophenol (29 μL, 43.3 mg) were converted to **22** yielding a yellow solid (41.0 mg, 85%) after purification by column chromatography (10% EtOAc/*n*-pentane).

$\mathbf{R_f}$ = 0.15 (EtOAc/*n*-pentane 1:9).

^{1}H-NMR (400 MHz, $CDCl_3$): δ (ppm) = 8.04 (d, *J* = 16.1 Hz, 1H), 7.47 (dd, *J* = 7.7, 1.7 Hz, 1H), 7.23 (dd, *J* = 8.1, 1.7 Hz, 1H), 6.92 (td, *J* = 7.7, 1.2 Hz, 1H), 6.86 (dd, *J* = 8.1, 1.2 Hz, 1H), 6.69 (s, 1H), 6.64 (d, *J* = 16.1 Hz, 1H), 4.29 (q, *J* = 7.2 Hz, 2H), 1.35 (t, *J* = 7.2 Hz, 3H).

Analytical data in agreement with literature.[26]

Synthesis of ethyl (E)-3-(2-hydroxyphenyl)-2-methylacrylate (23)

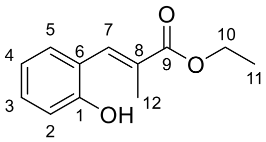

According to General Procedure **D**, ***E*-21** (120.1 mg) and 2-bromophenol (29 μL, 43.3 mg) were converted to **23** yielding a yellow gum (30.0 mg, 58%) after purification by column chromatography (10% EtOAc/*n*-pentane).

$\mathbf{R_f}$ = 0.19 (EtOAc/*n*-pentane 1:9).

^{1}H-NMR (500 MHz, $CDCl_3$): δ (ppm) = 7.74 (s, 1H; H7), 7.25 – 7.20 (m, 2H, H5; H3), 6.94 (td, *J* = 7.5, 1.1 Hz, 1H, H4), 6.89 (d, *J* = 8.6 Hz, 1H, H2), 5.35 (br s, 1H, O*H*), 4.28 (q, *J* = 7.1 Hz, 2H, H10), 2.02 (d, *J* = 1.5 Hz, 3H, H12), 1.35 (t, *J* = 7.1 Hz, 3H, H11).

^{13}C-NMR (126 MHz, $CDCl_3$): δ (ppm) = 168.5 (C9), 153.8 (C1), 133.9 (C7), 130.7 (C8), 130.0 (C5/C3), 129.9 (C5/C3), 122.9 (C6), 120.4 (C4), 115.9 (C2), 61.1 (C10), 14.4 (C11), 14.3 (C12).

Ir (ATR) $\tilde{\nu}$ = 3313 (broad, w), 2981 (w), 2931 (w), 1702 (m), 1662 (m), 1602 (m), 1453 (m), 1367 (m), 1256 (s), 1114 (s), 1096 (s), 1029 (m), 945 (w), 808 (m), 753 (s), 666 (m) cm^{-1}.

HR-ESI-MS: exact mass calculated for $[M-H]^-$ $(C_{12}H_{13}O_3)^-$ requires m/z 205.0870, found m/z 205.0887; exact mass calculated for $[M+HCOO]^-$ $(C_{13}H_{15}O_5)^-$ requires m/z 251.0925, found m/z 251.0949.

Synthesis of 2*H*-chromen-2-one (24)

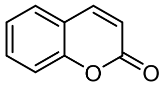

According to General Procedure **D**, ***Z*-20** (169.6 mg, 3.0 eq.) and 2-bromophenol (29 μL, 43.3 mg) were converted to 2*H*-chromen-2-one (**24**) yielding a colourless solid (20.1 mg, 55%) after purification by column chromatography (10% EtOAc/*n*-pentane).

$\mathbf{R_f}$ = 0.16 (EtOAc/*n*-pentane 1:9).

^{1}**H-NMR** (400 MHz, $CDCl_3$): δ (ppm) = 7.71 (d, *J* = 9.5 Hz, 1H), 7.58 – 7.46 (m, 2H), 7.34 (d, *J* = 7.9 Hz, 1H), 7.28 (t, *J* = 7.5 Hz, 1H), 6.43 (d, *J* = 9.5 Hz, 1H).

Analytical data in agreement with literature.[27]

Synthesis of 3-methyl-2*H*-chromen-2-one (25)

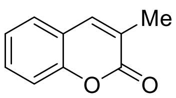

According to General Procedure **D**, ***Z*-21** (120.1 mg) and 2-bromophenol (29 μL, 43.3 mg) were converted to 3-methyl-2*H*-chromen-2-one (**25**) yielding a colourless solid (36.0 mg, 90%) after purification by column chromatography (10% EtOAc/*n*-pentane).

$\mathbf{R_f}$ = 0.31 (EtOAc/*n*-pentane 1:9).

^{1}**H-NMR** (300 MHz, $CDCl_3$): δ (ppm) = 7.52 (s, 1H), 7.46 (t, *J* = 7.8 Hz, 1H), 7.42 (dd, *J* = 7.7, 1.6 Hz, 1H), 7.31 (d, *J* = 8.2 Hz, 1H), 7.25 (t, *J* = 7.5 Hz, 1H), 2.22 (d, *J* = 1.4 Hz, 3H).

Analytical data in agreement with literature.[28]

Synthesis of 2H-furo[2,3-h]chromen-2-one (26)

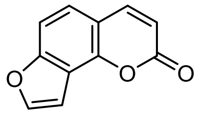

According to General Procedure **D**, ***Z*-20** (169.6 mg, 3.0 eq.) and **S17** (53.3 mg) were converted to 2*H*-furo[2,3-*h*]chromen-2-one (**26**) yielding a yellow solid (22.0 mg, 47%) after purification by column chromatography (30% EtOAc/*n*-pentane).

$\mathbf{R_f}$ = 0.65 (EtOAc/*n*-pentane 3:7).

^{1}**H-NMR** (400 MHz, $CDCl_3$): δ (ppm) = 7.81 (d, *J* = 9.6 Hz, 1H), 7.70 (d, *J* = 2.2 Hz, 1H), 7.44 (dd, *J* = 8.5, 0.9 Hz, 1H), 7.38 (d, *J* = 8.5 Hz, 1H), 7.13 (dd, *J* = 2.2, 0.9 Hz, 1H), 6.39 (d, *J* = 9.5 Hz, 1H).

Analytical data in agreement with literature.[29]

Synthesis of ethyl (*E*)-3-(4-hydroxybenzofuran-5-yl)acrylate (27)

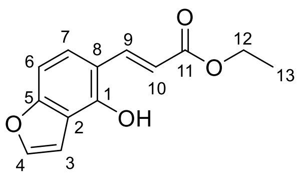

According to General Procedure **D**, ***E*-20** (45.2 mg) and **S17** (21.3 mg, 0.1 mmol) were converted to ethyl (*E*)-3-(4-hydroxybenzofuran-5-yl)acrylate (**27**) yielding a yellow solid (8.1 mg, 35%) after purification by column chromatography (20% EtOAc/*n*-pentane).

$\mathbf{R_f}$ = 0.22 (EtOAc/*n*-pentane 1:9).

^{1}H-NMR (599 MHz, Methanol-d_4): δ (ppm) = 8.12 (d, J = 16.1 Hz, 1H, H9), 7.63 (d, J = 2.2 Hz, 1H, H4), 7.46 (d, J = 8.7 Hz, 1H, H7), 7.05 – 7.00 (m, 2H, H3, H6), 6.49 (d, J = 16.1 Hz, 1H, H10), 4.21 (q, J = 7.1 Hz, 2H, H12), 1.31 (t, J = 7.1 Hz, 3H, H13).

^{13}C-NMR (126 MHz, Methanol-d_4): δ (ppm) = 169.8 (C11), 159.1 (C5), 152.2 (C1), 145.4 (C4), 142.2 (C9), 126.0 (C7), 118.5 (C2), 116.5 (C8), 116.4 (C10), 105.3 (C6), 105.0 (C3), 61.4 (C12), 14.7 (C13).

IR (ATR) $\tilde{\nu}$ = 3195 (w), 2977 (w), 2928 (w), 1732 (w), 1674 (m), 1606 (m), 1475 (w), 1442 (w), 1355 (m), 1320 (s), 1291 (m), 1227 (m), 1179 (s), 1141 (s), 1054 (m), 1030 (m), 1004 (m), 854 (m), 782 (m), 747 (s) cm^{-1}

HR-ESI-MS (Orbitrap): exact mass calculated for $[M+Na]^+$ $(C_{13}H_{12}O_4Na)^+$ requires m/z 255.06278, found m/z 255.06258.

M.p.: 123.5 – 125.4 °C.

3. X-ray Crystallographic Analysis

X-Ray diffraction: Data sets for compound **18** were collected with a Bruker D8 Venture CMOS diffractometer. Programs used: data collection: APEX3 V2016.1-0 (Bruker AXS Inc., **2016**); cell refinement: SAINT V8.37A (Bruker AXS Inc., **2015**); data reduction: SAINT V8.37A (Bruker AXS Inc., **2015**); absorption correction, SADABS V2014/7 (Bruker AXS Inc., **2014**); structure solution *SHELXT-2015* (Sheldrick, G. M. *Acta Cryst.,* **2015**, *A71*, 3-8); structure refinement *SHELXL-2015* (Sheldrick, G. M. *Acta Cryst.,* **2015**, *C71* (1), 3-8) and graphics, *XP* (Version 5.1, Bruker AXS Inc., Madison, Wisconsin, USA, **1998**). *R*-values are given for observed reflections, and wR^2 values are given for all reflections.

X-ray crystal structure analysis of 18 : A colorless prism-like specimen of $C_{22}H_{24}O_3$, approximate dimensions 0.139 mm x 0.167 mm x 0.175 mm, was used for the X-ray crystallographic analysis. The X-ray intensity data were measured on a Bruker D8 Venture PHOTON III Diffractometer system equipped with a micro focus tube Cu Ims (CuKα, λ = 1.54178 Å) and a MX mirror monochromator. A total of 563 frames were collected. The total exposure time was 3.79 hours. The frames were integrated with the Bruker SAINT software package using a wide-frame algorithm. The integration of the data using an orthorhombic unit cell yielded a total of 15783 reflections to a maximum θ angle of 68.27° (0.83 Å resolution), of which 3156 were independent (average redundancy 5.001, completeness = 98.9%, R_{int} = 3.29%, R_{sig} = 2.53%) and 3040 (96.32%) were greater than 2σ(F^2). The final cell constants of a = 6.5555(2) Å, b = 14.0958(4) Å, c = 18.8222(5) Å, volume = 1739.27(9) $Å^3$, are based upon the refinement of the XYZ-centroids of 9887 reflections above 20 σ(I) with 11.30° < 2θ < 136.5°. Data were corrected for absorption effects using the Multi-Scan method (SADABS). The ratio of minimum to maximum apparent transmission was 0.898. The calculated minimum and maximum transmission coefficients (based on crystal size) are 0.8920 and 0.9130. The structure was solved and refined using the Bruker SHELXTL Software Package, using the space group $P2_12_12_1$, with Z = 4 for the formula unit, $C_{22}H_{24}O_3$. The final anisotropic full-matrix least-squares refinement on F^2 with 228 variables converged at R1 = 2.80%, for the observed data and wR2 = 7.54% for all data. The goodness-of-fit was 1.038. The largest peak in the final difference electron density synthesis was 0.210 $e^-/Å^3$ and the largest hole was -0.151 $e^-/Å^3$ with an RMS deviation of 0.032 $e^-/Å^3$. On the basis of the final model, the calculated density was 1.285 g/cm^3 and F(000), 720 e^-. Flack parameter was refined to 0.00(6). CCDC number: 2022960.

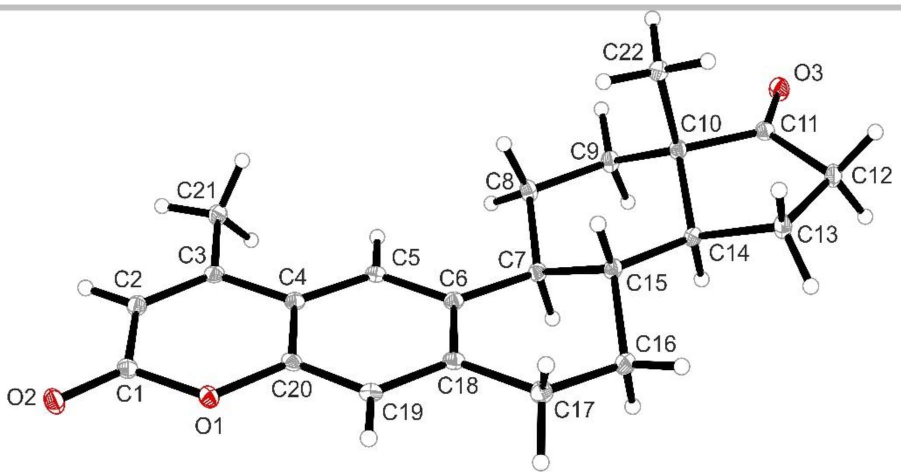

Figure 2a: Crystal structure of compound **18**.
Thermal ellipsoids are shown at 30% probability.

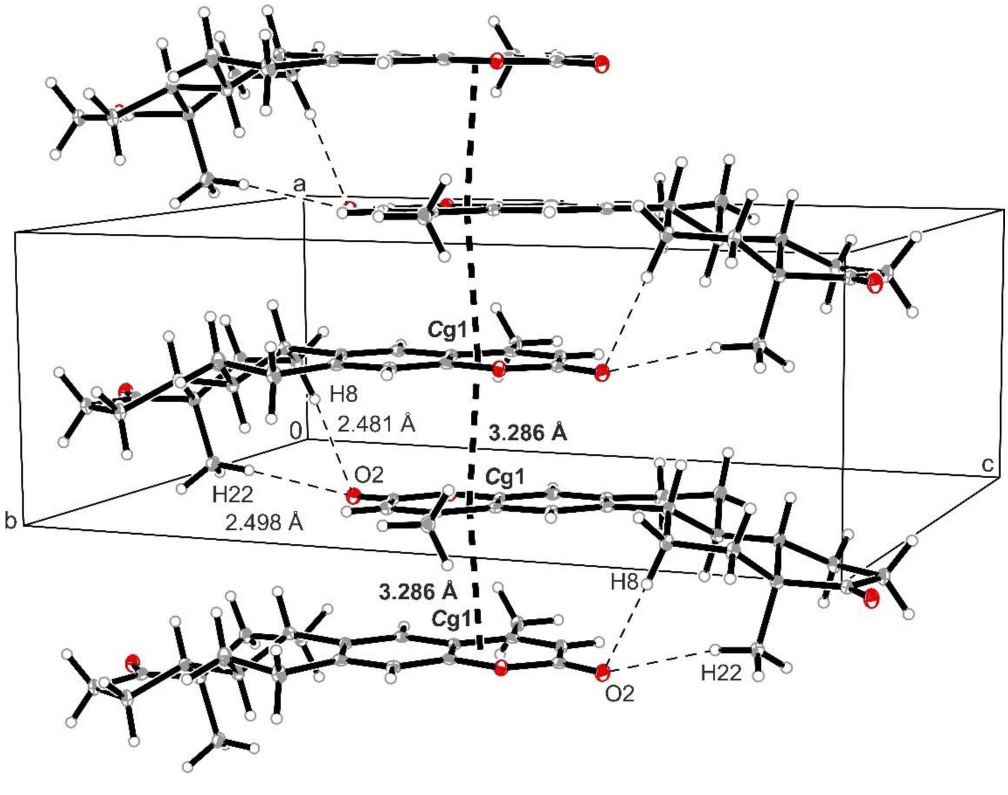

Figure 2b: Excerpt of the packing diagram of compound **18** presenting the formation of a linear chain along "*a*"-axis involving $\pi\cdots\pi$ and CH···O interactions.

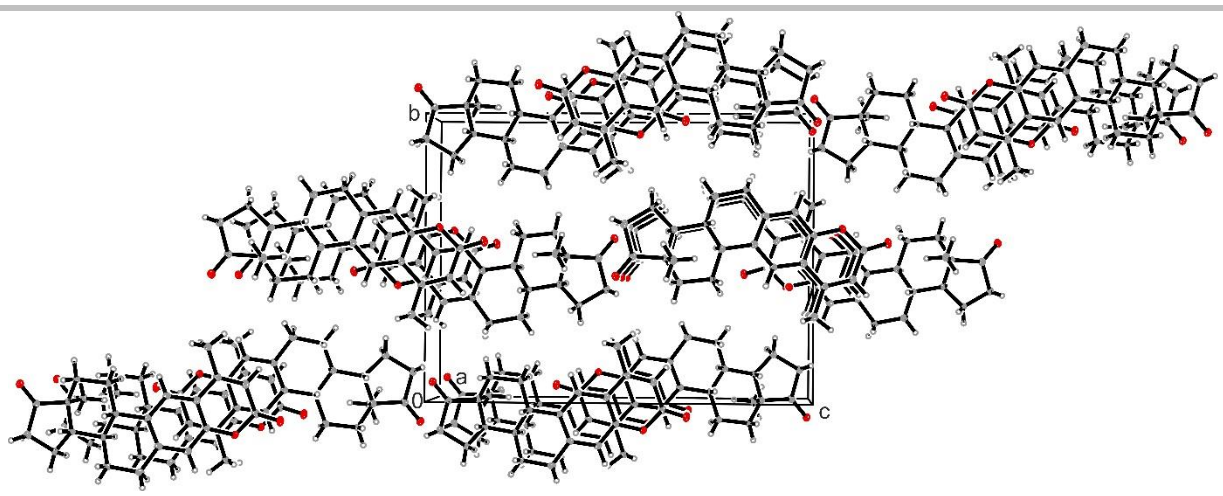

Figure 2c: Overview of the 3D network of compound **18.**

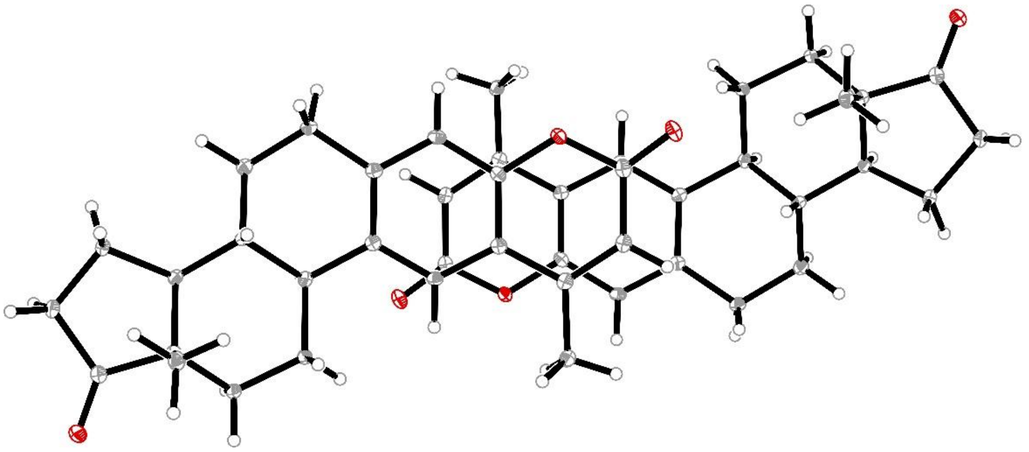

Figure 2d: Overlapping mode involving π···π interactions between the aromatic units in compound **18**.

Table S2: Non-covalent intermolecular interactions in compound **18** (Å and deg)

D-H···*A*	*d*(*D*-H)	*d*(H···*A*)	*d*(*D*···*A*)	∠(*D*H*A*)
C13-H13A···O1[#1]	0.99	2.64	3.474(2)	142.5
C8-H8B···O2[#2]	0.99	2.48	3.418(2)	157.9
C22-H22B···O2[#2]	0.98	2.50	3.434(2)	159.6
C12-H12A···O3[#3]	0.99	2.57	3.368(2)	137.3
C12-H12B···O3[#4]	0.99	2.65	3.401(2)	133.3
*C*g1···*C*g1[#5]			3.286	

Symmetry transformations used to generate equivalent atoms: [#1] -x+1.5, -y+1, z-0.5; [#2] x+0.5, -y+1.5, -z+1; [#3] x-0.5, -y+1.5, -z+2; [#4] x+0.5, -y+1.5, z-2; [#5] x+0.5, -y+1.5, -z+1.

1. *APEX3* (**2016**), *SAINT* (**2015**) and *SADABS* (**2015**), Bruker AXS Inc., Madison, Wisconsin, USA.
2. Sheldrick, G. M., *SHELXT – Integrated space-group and crystal-structure determination*, *Acta Cryst.,* **2015**, *A71*, 3-8.
3. Sheldrick, G.M., *Crystal structure refinement with SHELXL*, *Acta Cryst.*, **2015**, *C71 (1)*, 3-8.
4. *XP – Interactive molecular graphics, Version 5.1,* Bruker AXS Inc., Madison, Wisconsin, USA, **1998**.

4. NMR-Spectra of Key Compounds

^{1}H-NMR of S1 (400 MHz, $CDCl_3$)

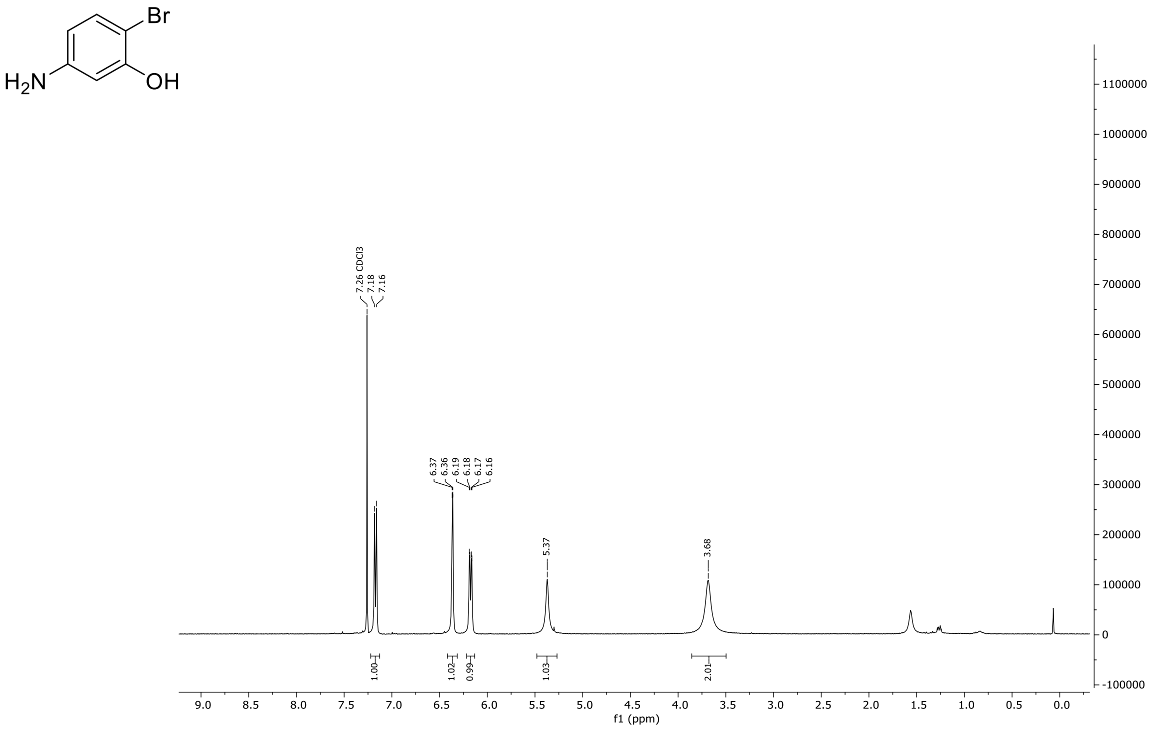

^{1}H-NMR of S2 (300 MHz, $CDCl_3$)

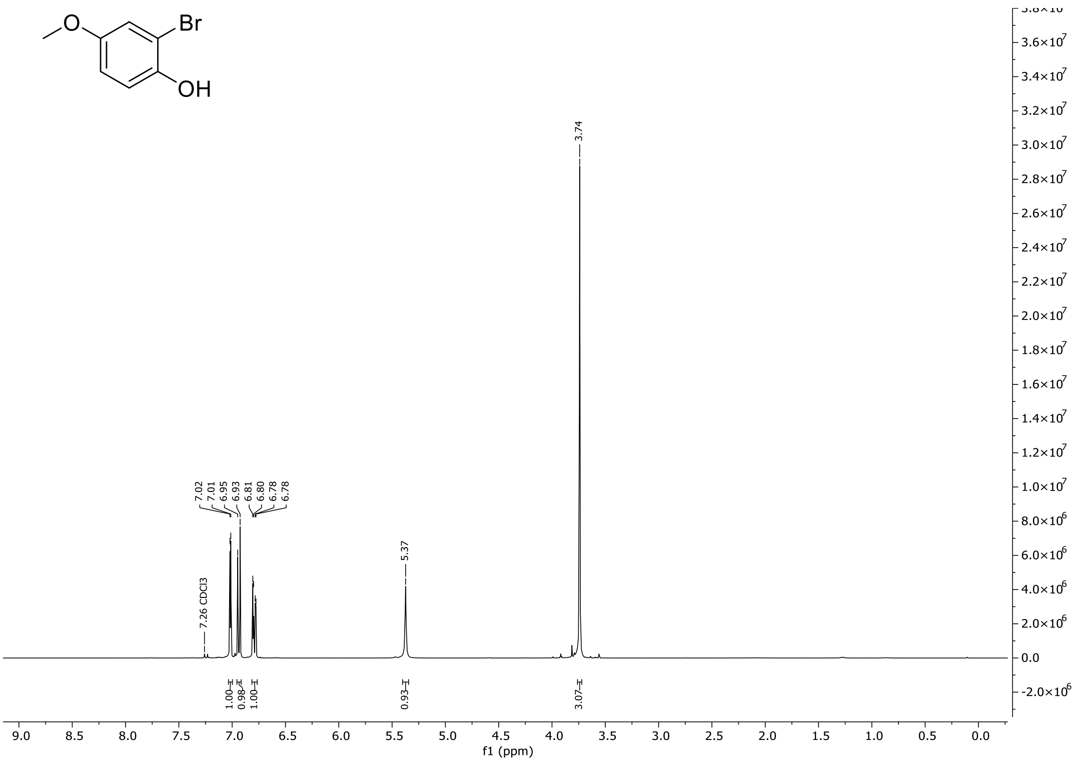

^{1}H-NMR of S3 (300 MHz, $CDCl_3$)

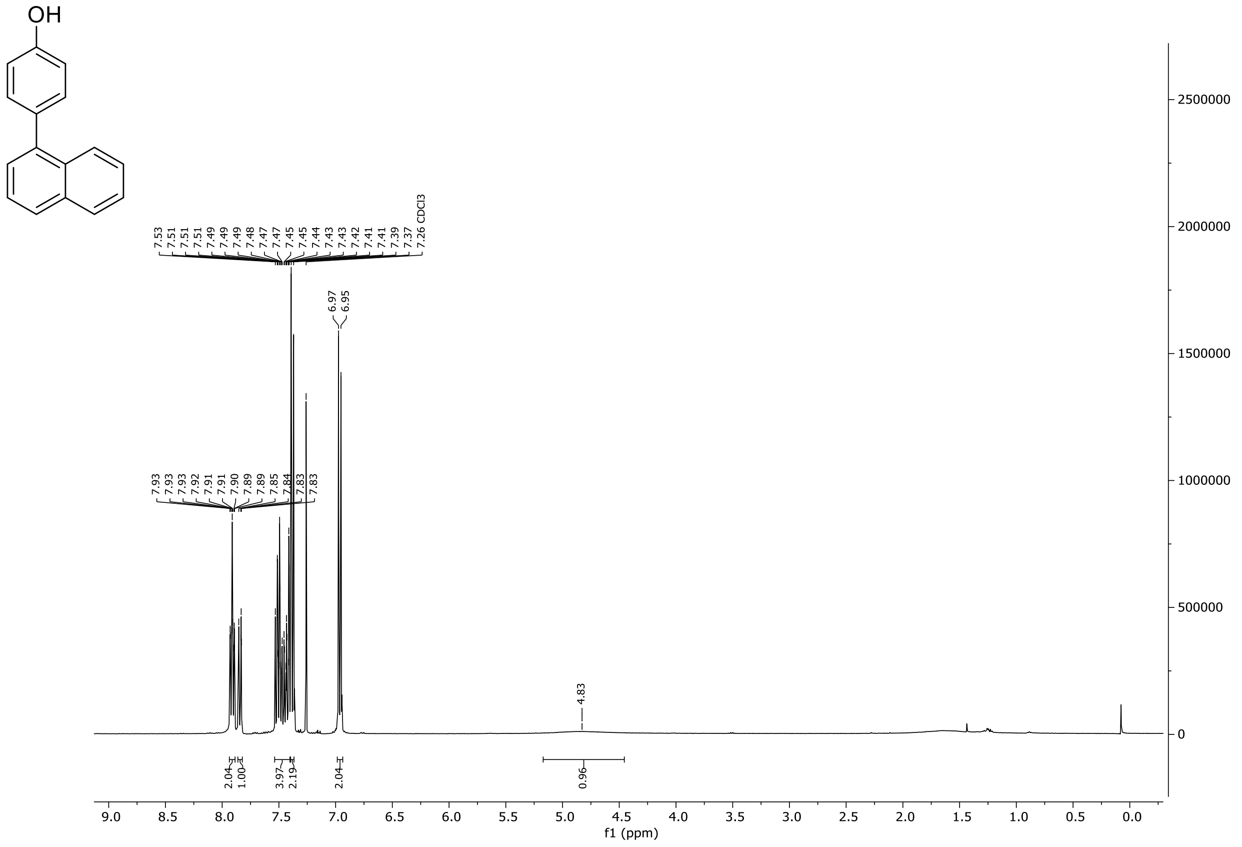

^{1}H-NMR of S4 (599 MHz, $CDCl_3$)

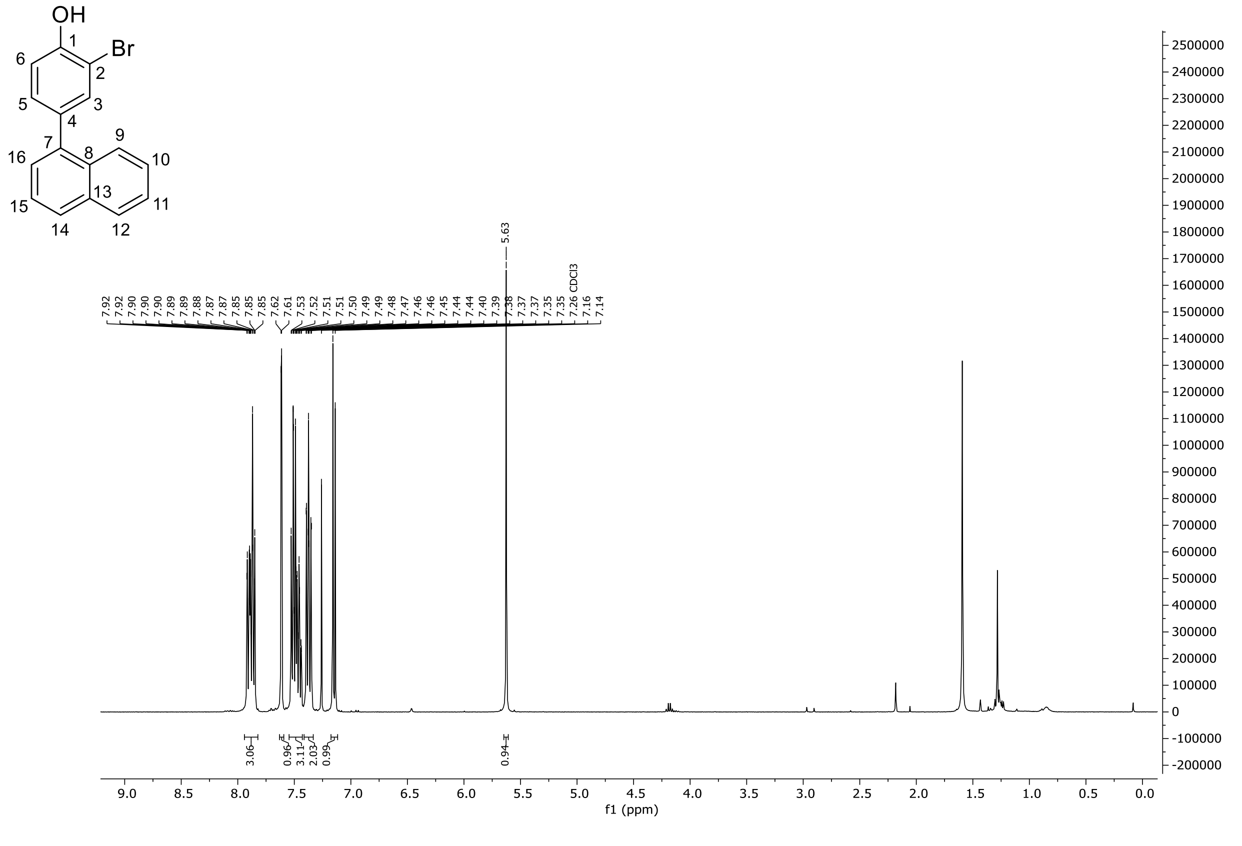

^{13}C-NMR of S4 (151 MHz, $CDCl_3$)

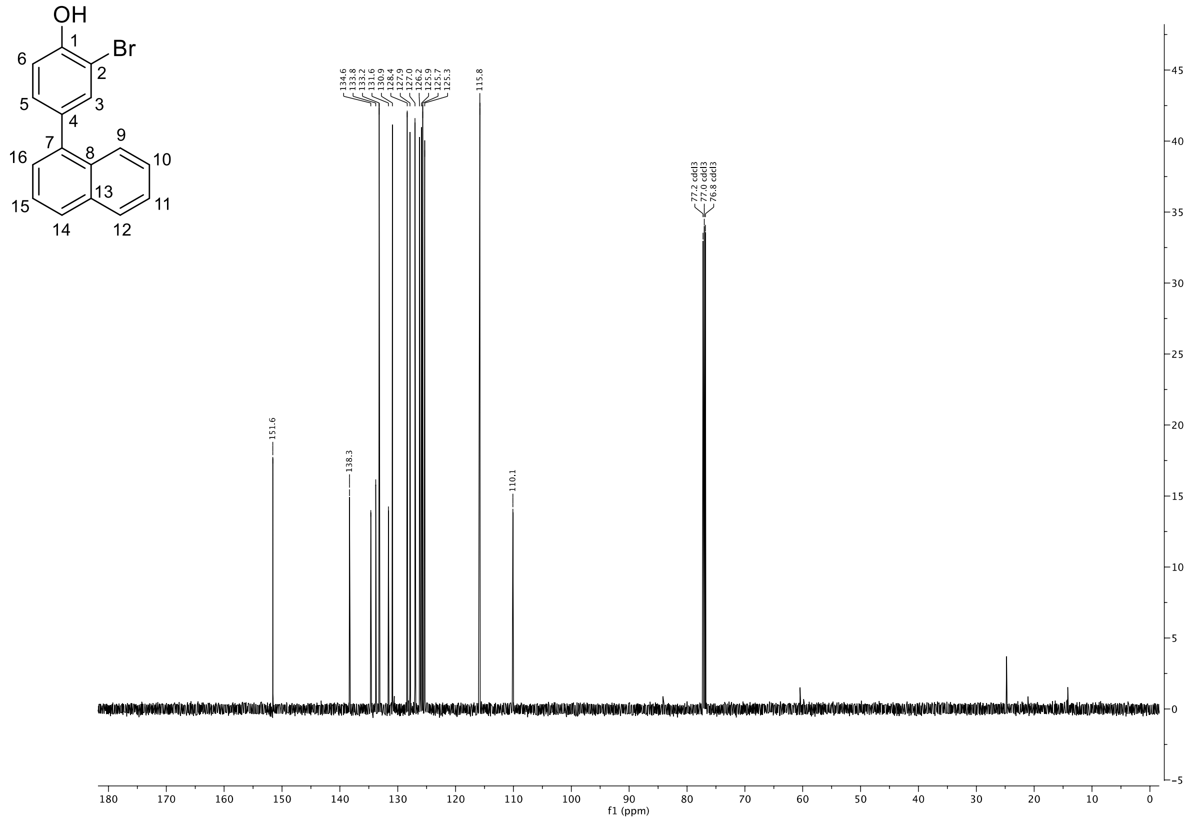

^{1}H-NMR of S5 (400 MHz, $CDCl_3$)

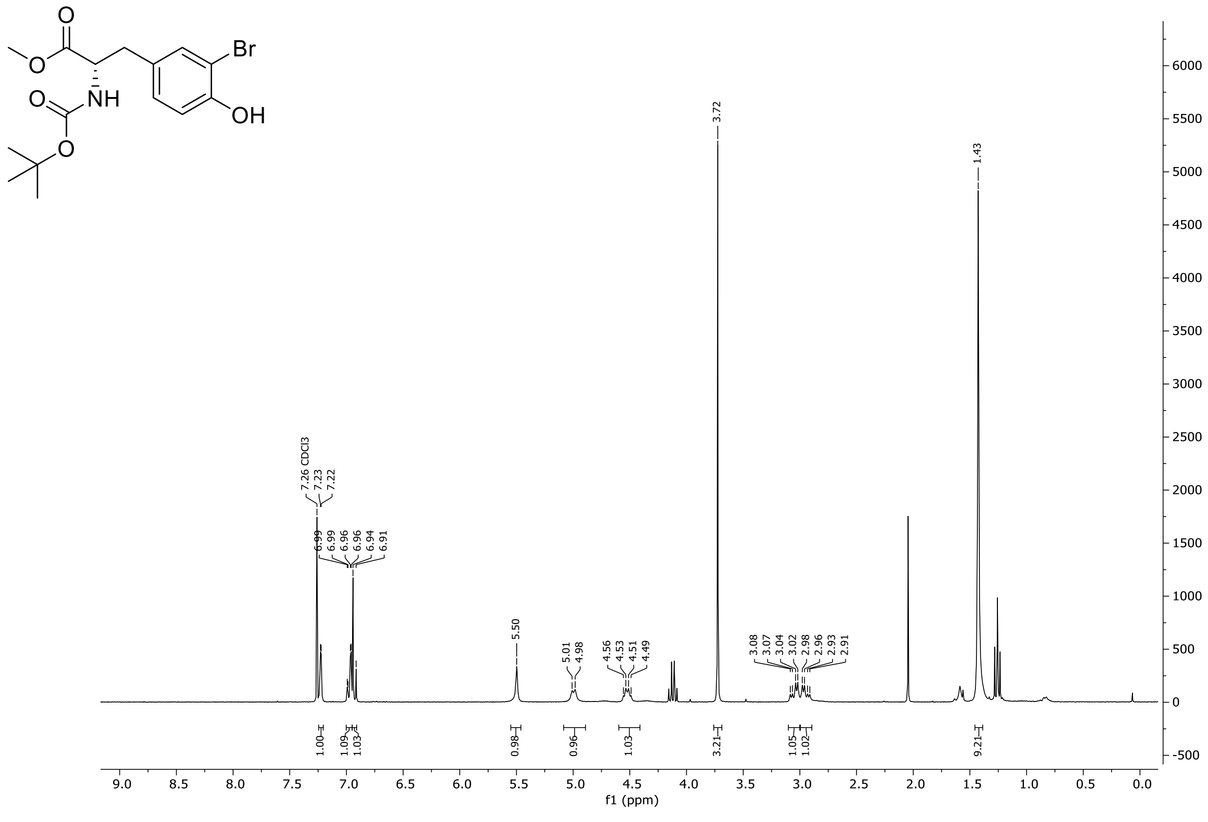

^{1}H-NMR of S6 (599 MHz, $CDCl_3$)

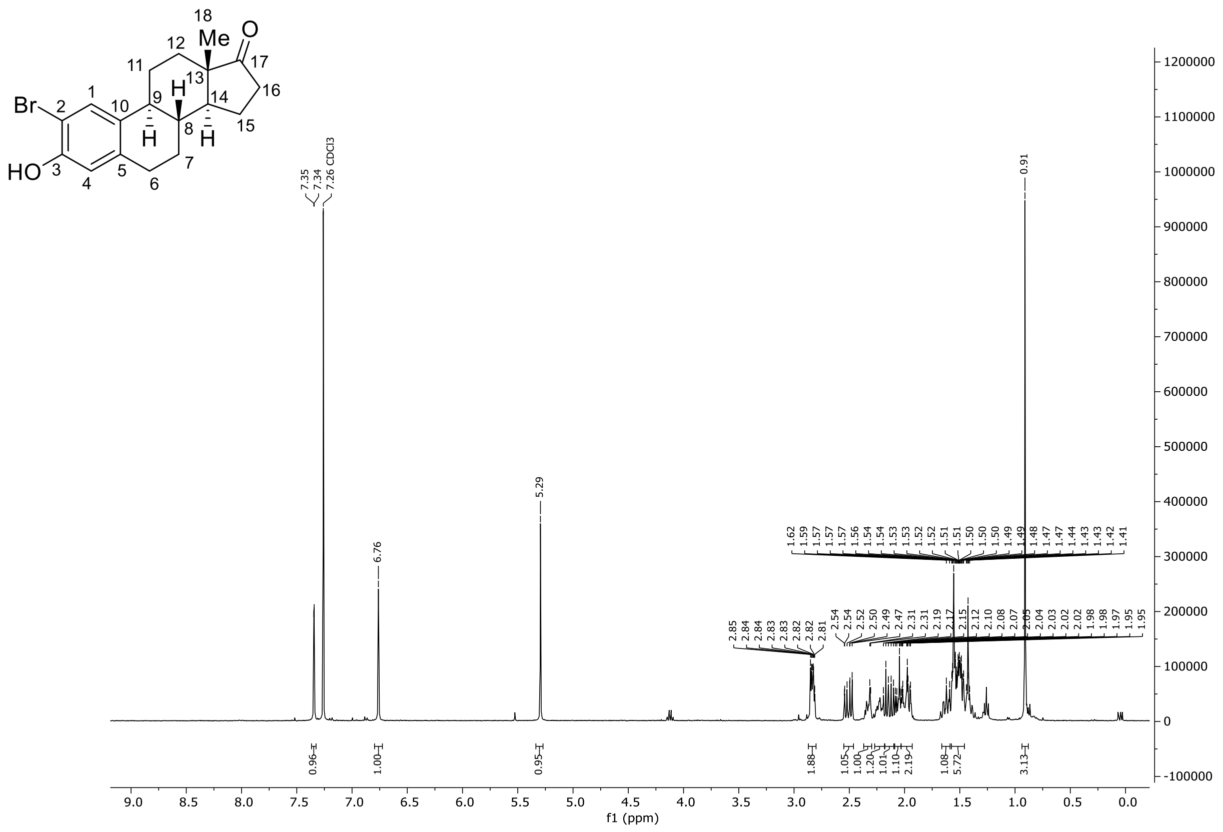

^{13}C-NMR of S6 (151 MHz, $CDCl_3$)

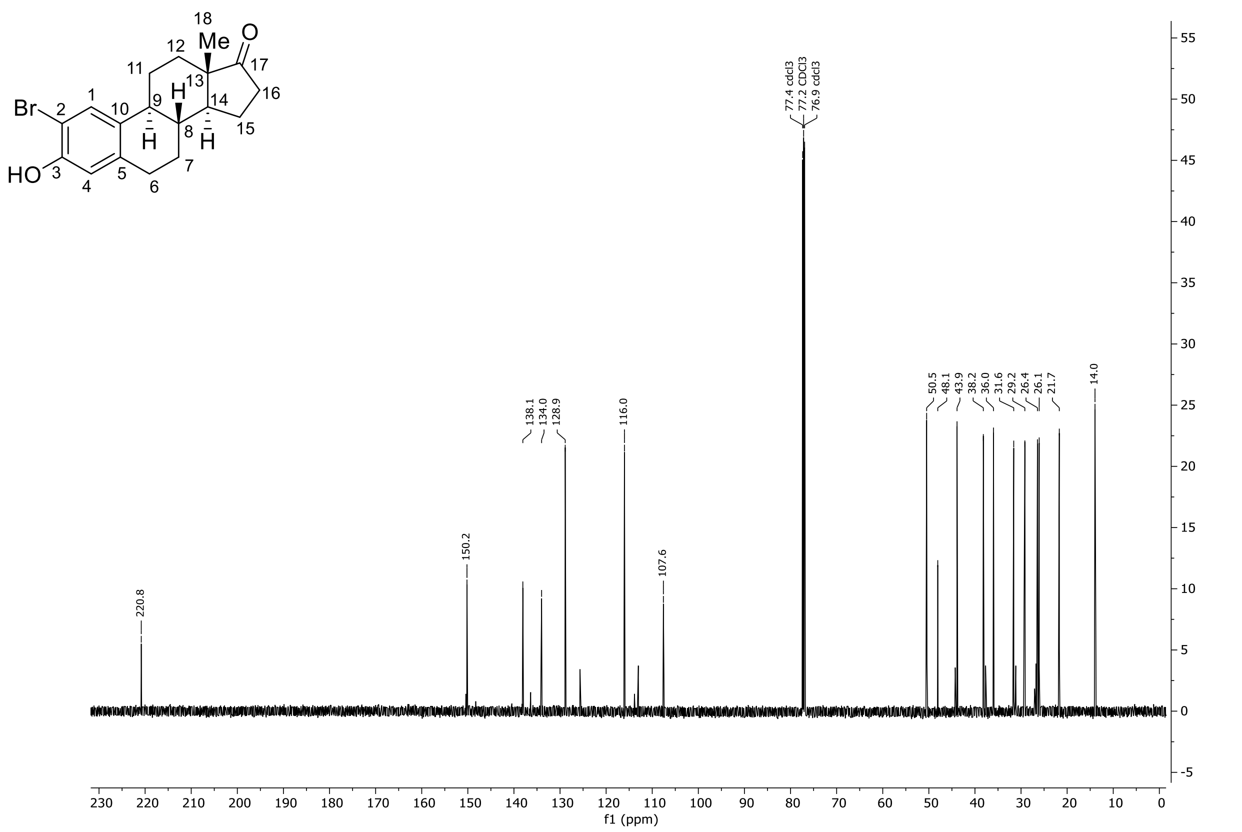

^{1}H-NMR of S7 (599 MHz, $CDCl_3$)

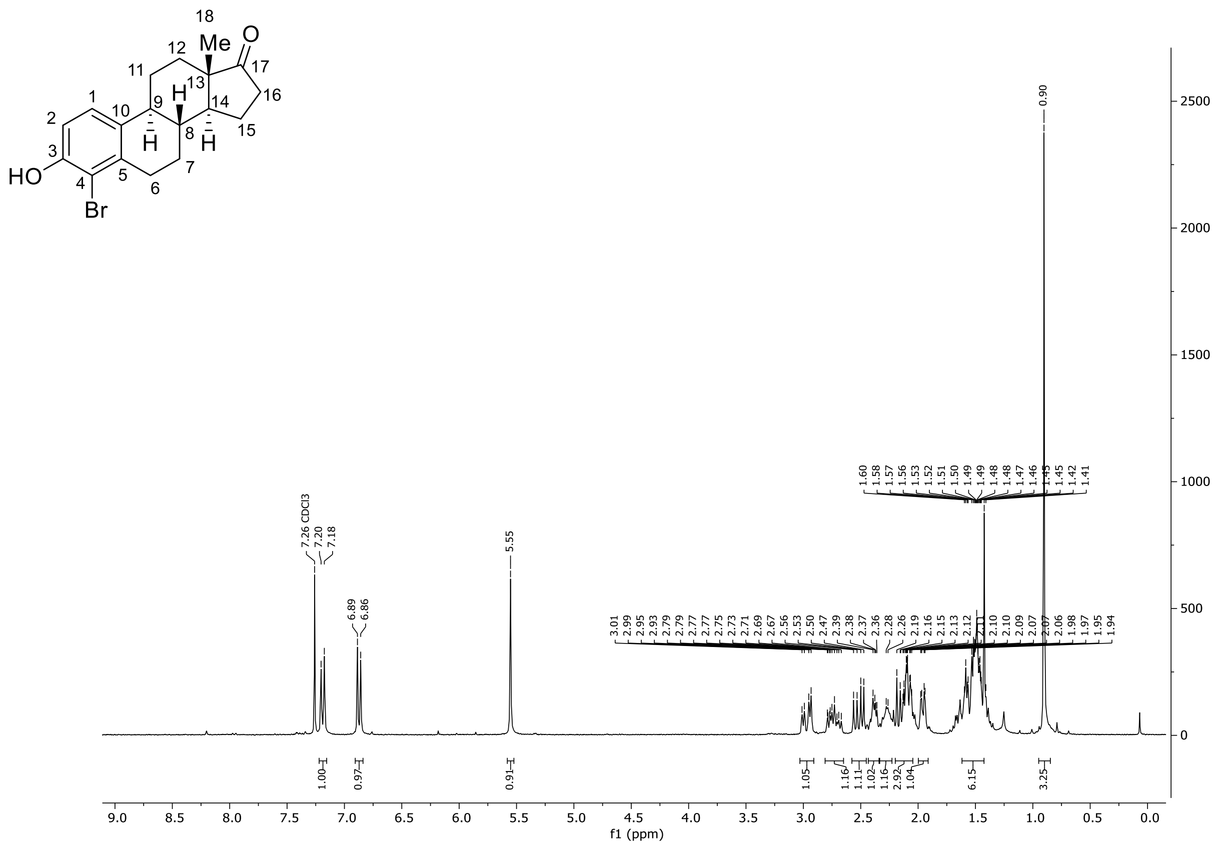

^{13}C-NMR of S7 (151 MHz, $CDCl_3$)

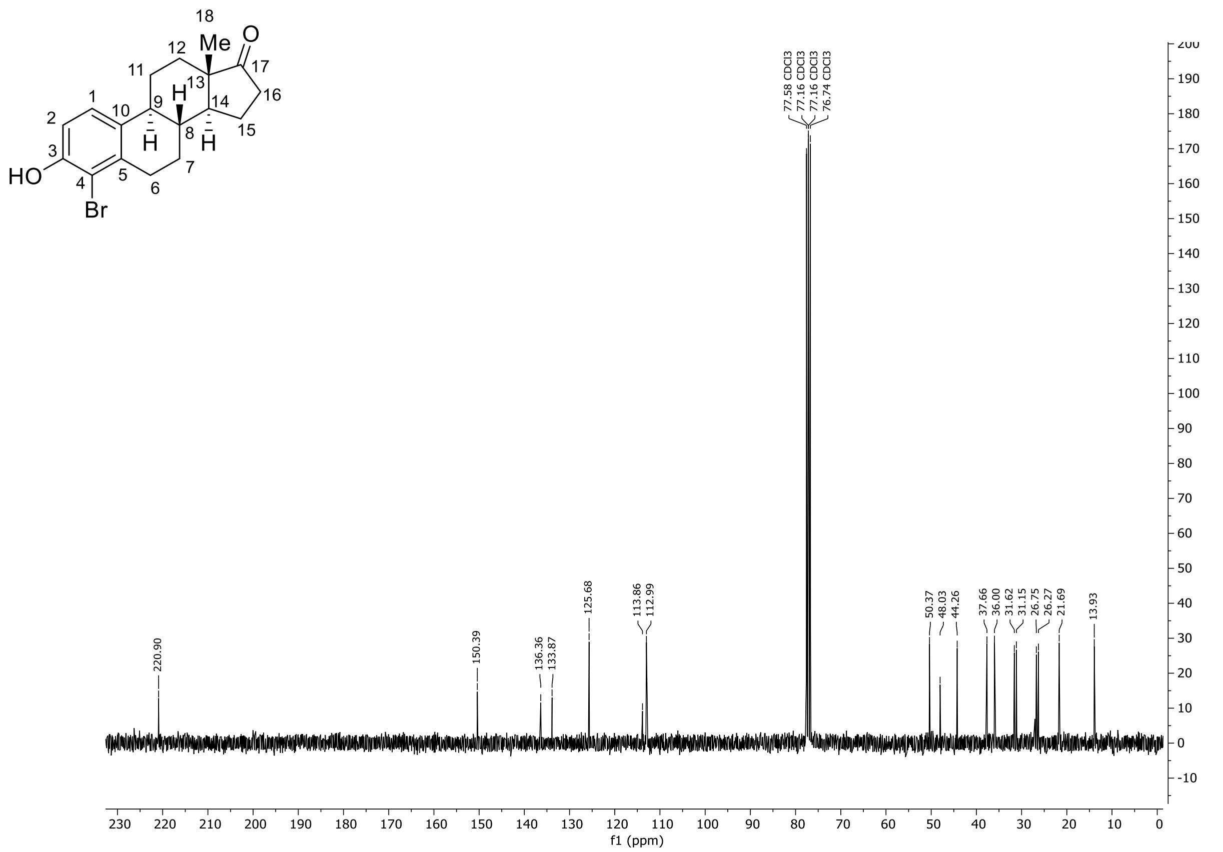

^{1}H-NMR of S8 (599 MHz, $CDCl_3$)

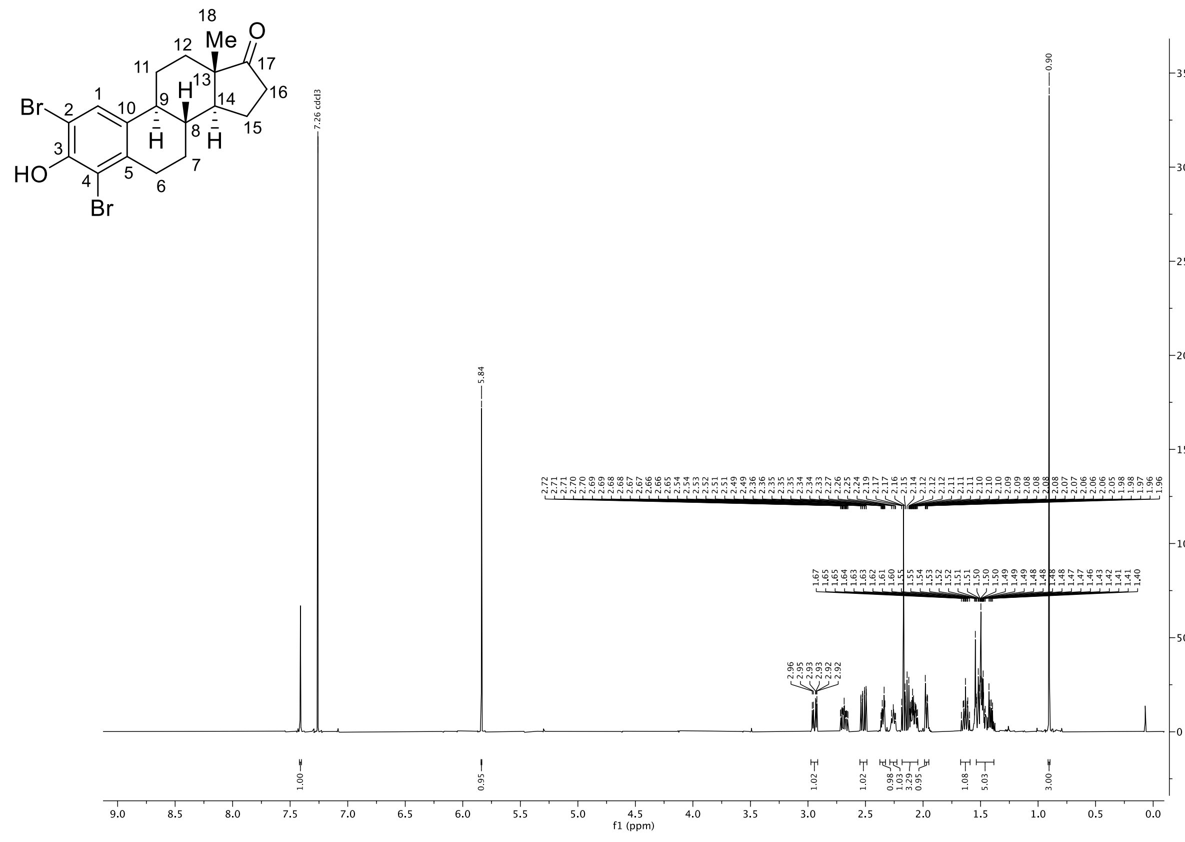

^{13}C-NMR of S8 (151 MHz, $CDCl_3$)

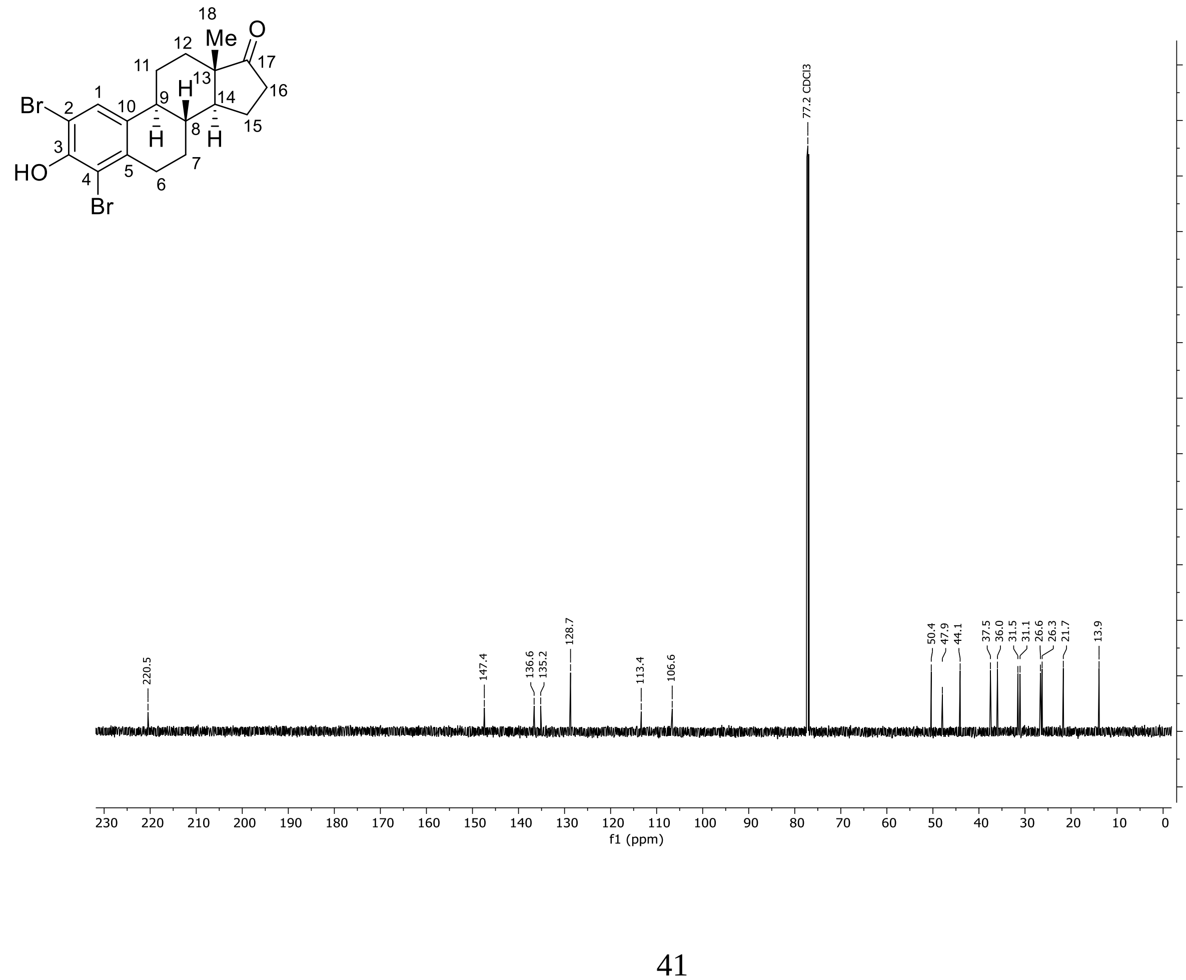

^{1}H-NMR of S9 (300 MHz, $CDCl_3$)

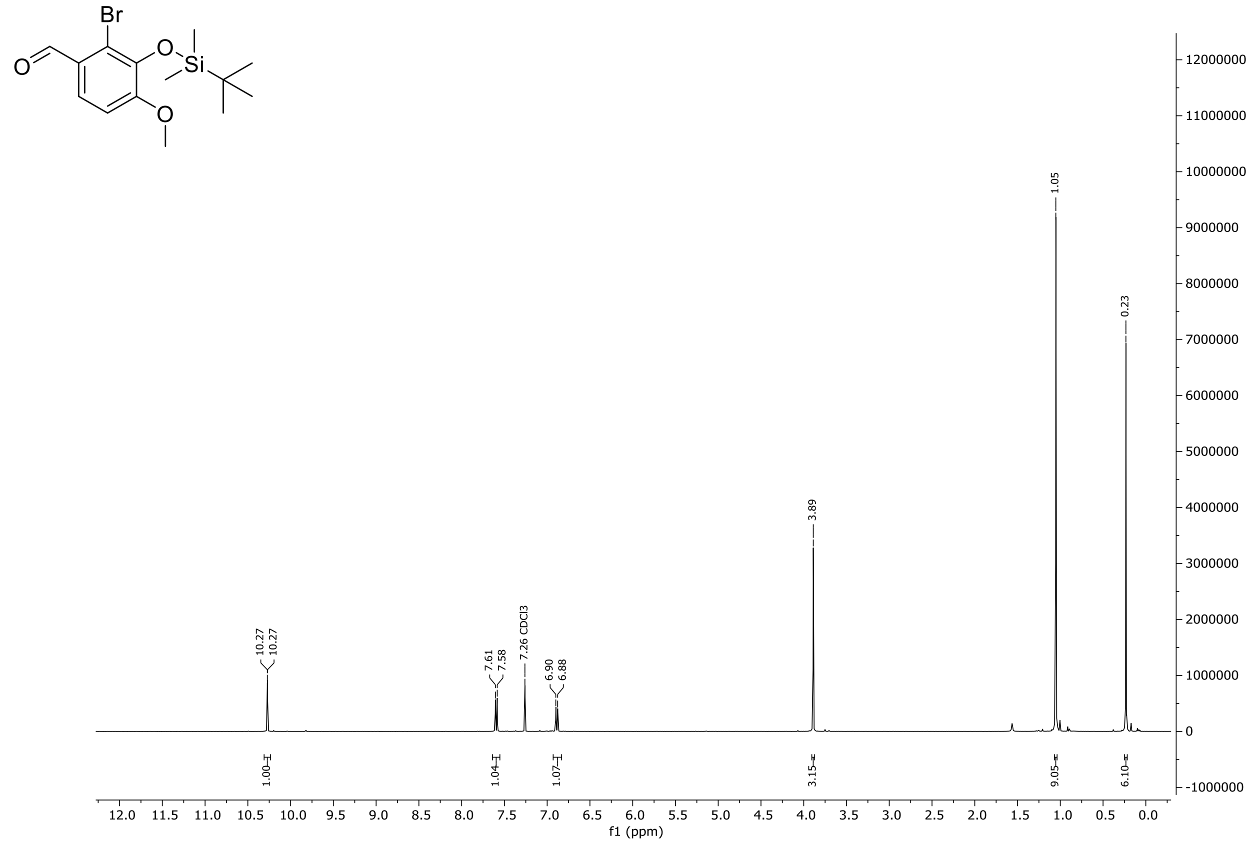

^{1}H-NMR of S10 (300 MHz, $CDCl_3$)

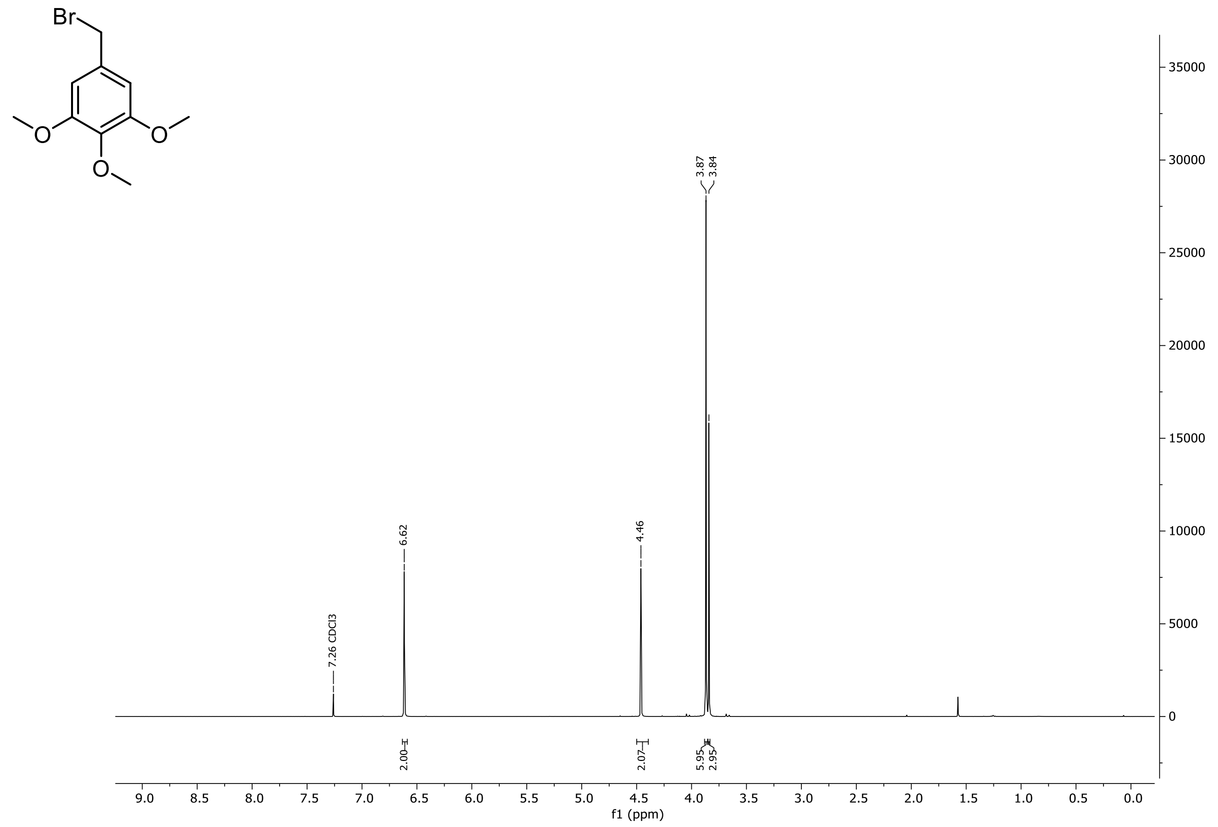

^{1}H-NMR of S11 (300 MHz, $CDCl_3$)

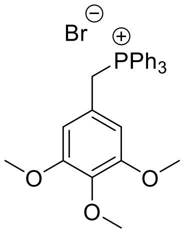

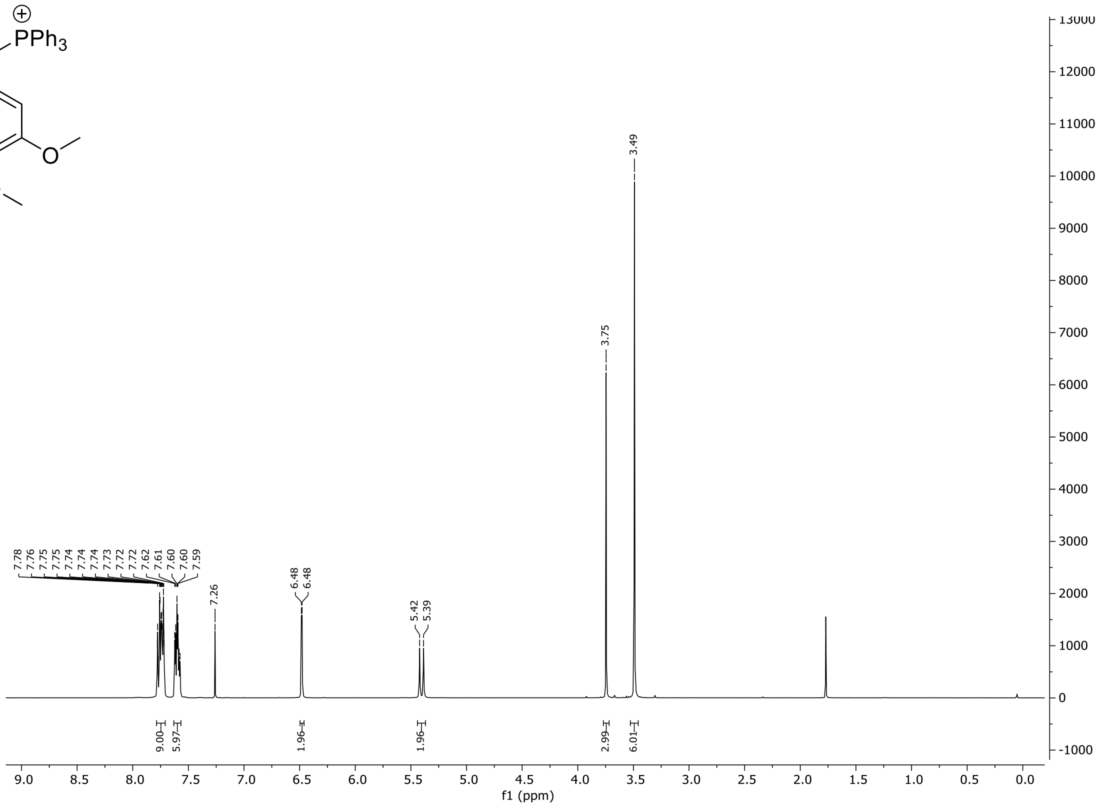

^{31}P-NMR of S11 (300 MHz, $CDCl_3$)

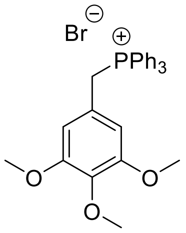

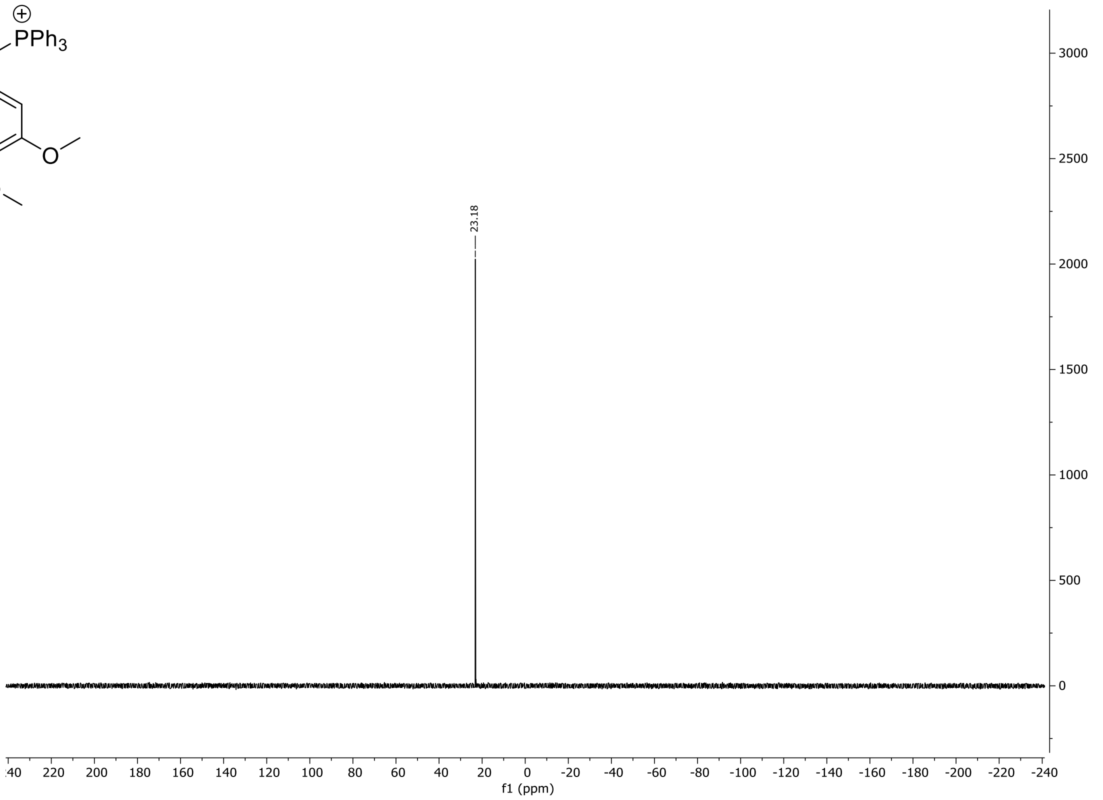

^{1}H-NMR of S12 (400 MHz, $CDCl_3$)

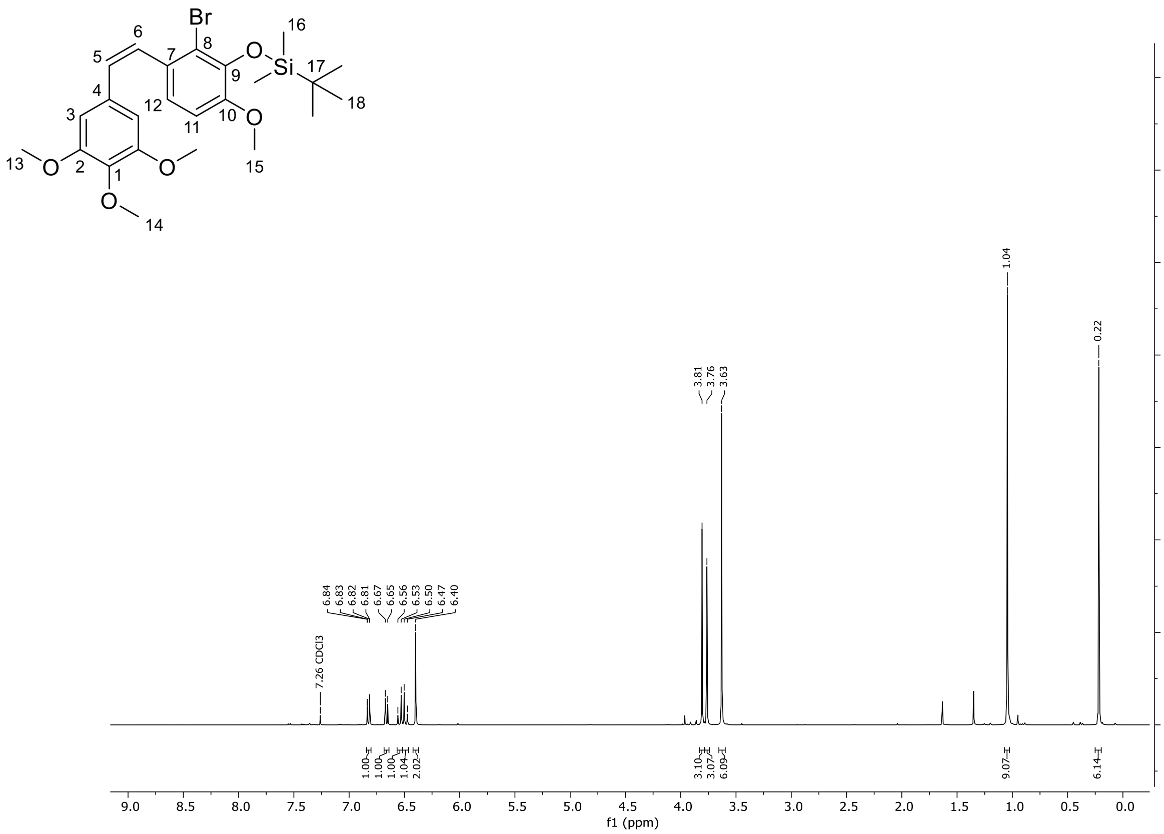

^{13}C-NMR of S12 (126 MHz, $CDCl_3$)

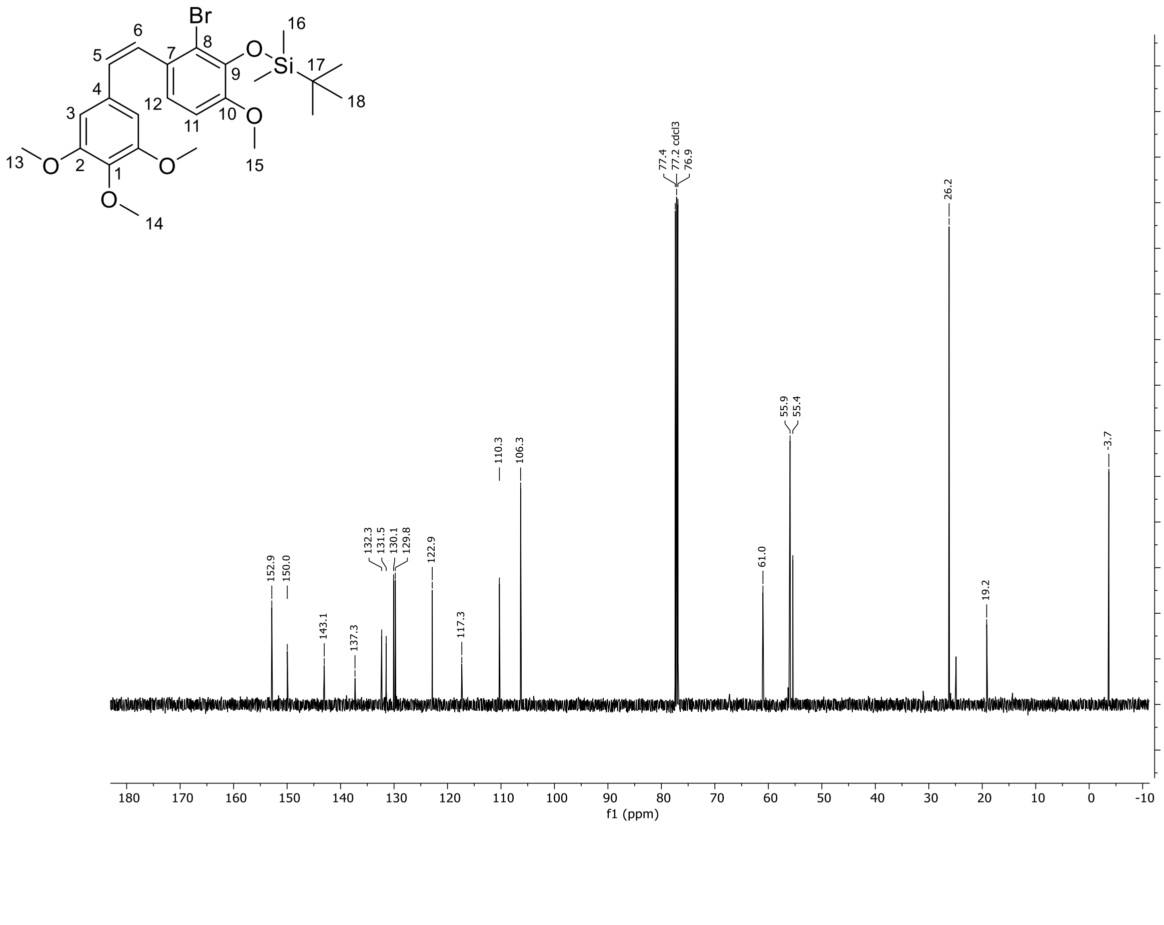

^{1}H-NMR of S13 (400 MHz, $CDCl_3$)

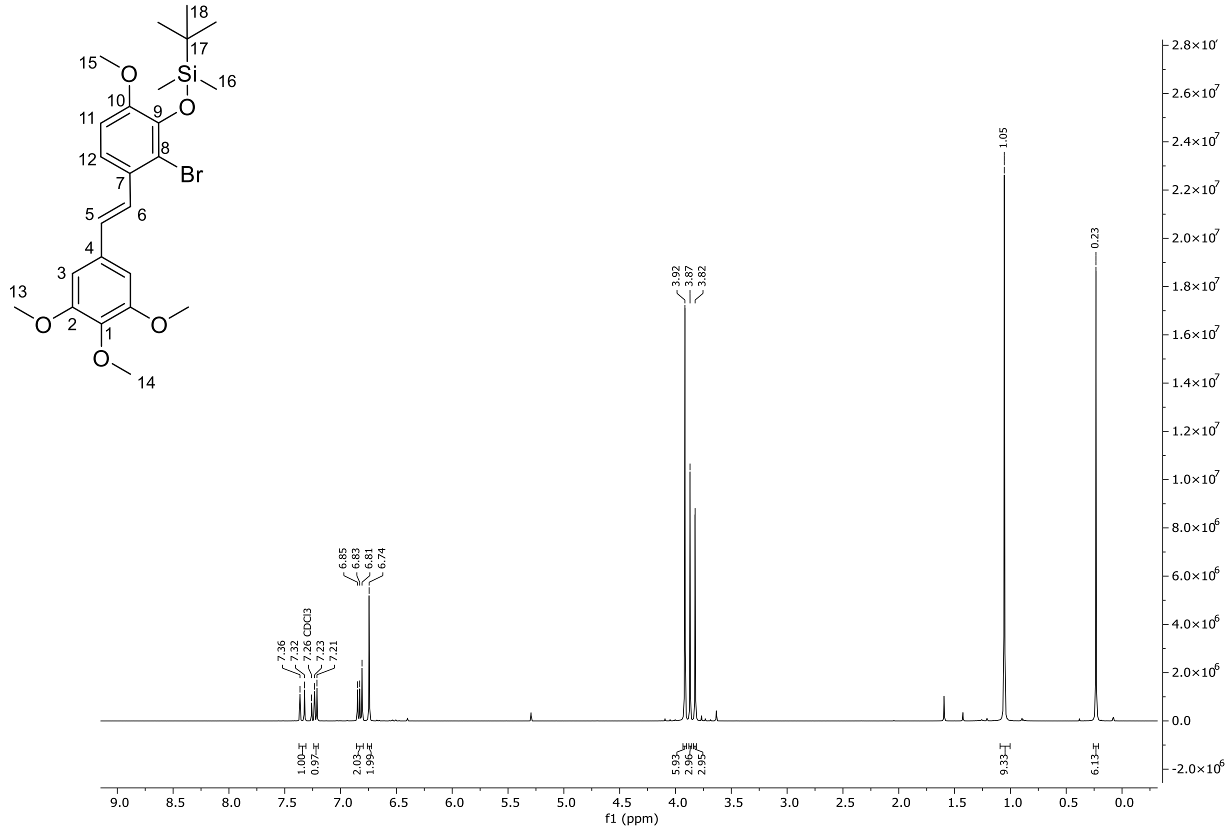

^{13}C-NMR of S13 (151 MHz, $CDCl_3$)

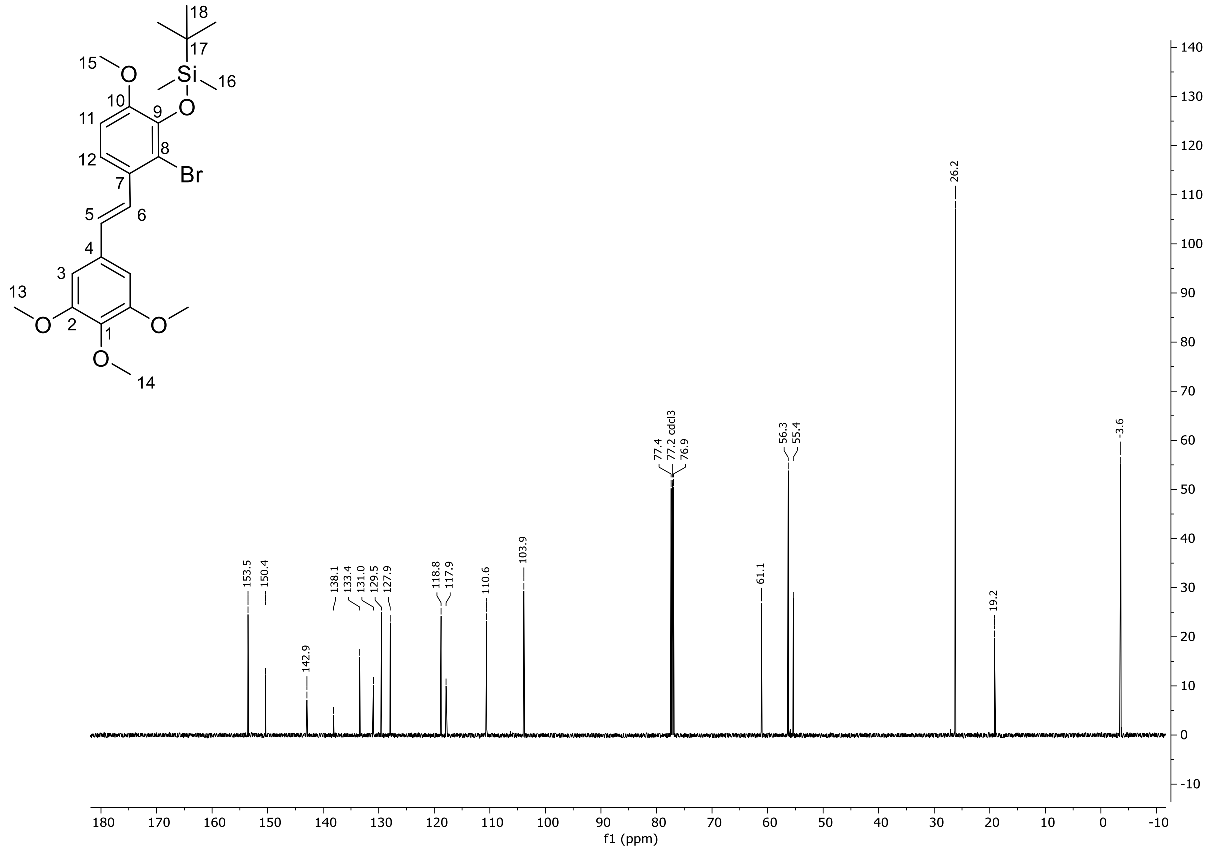

^{1}H-NMR of S14 (400 MHz, $CDCl_3$)

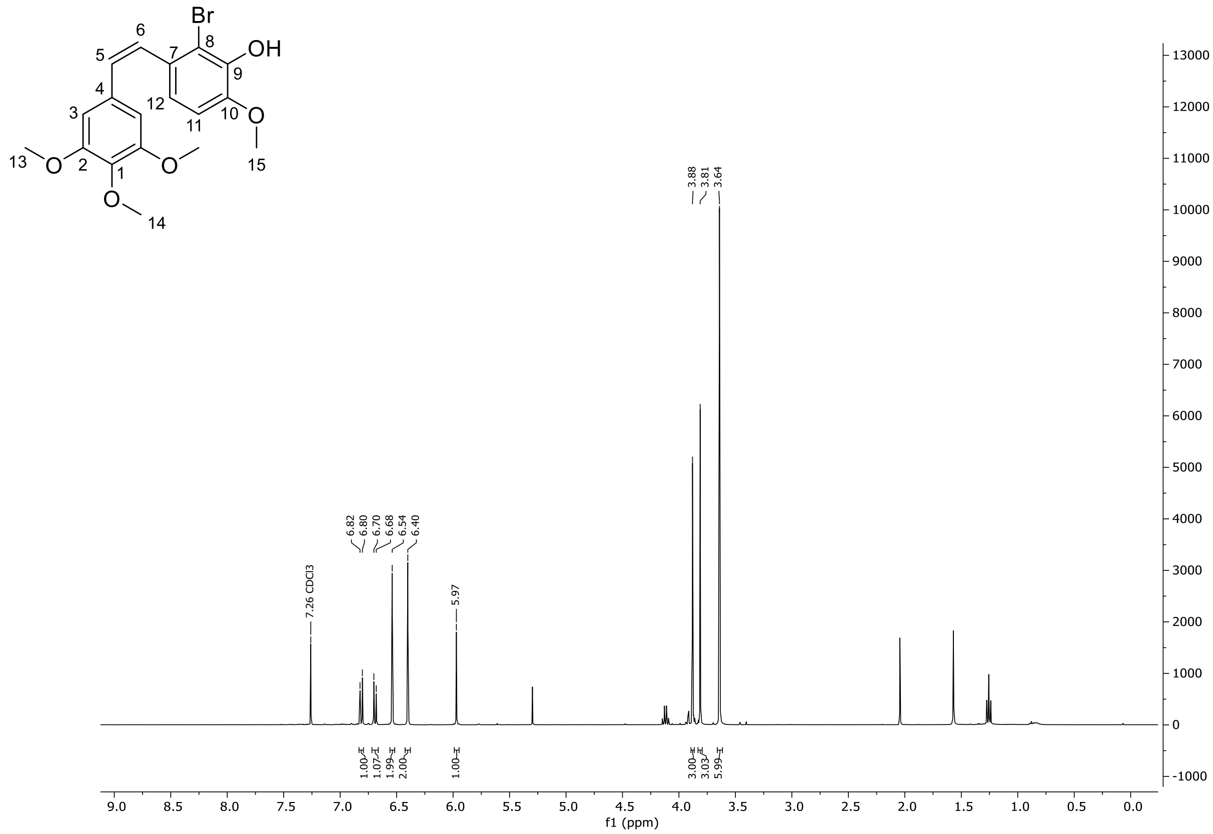

^{13}C-NMR of S14 (151 MHz, $CDCl_3$)

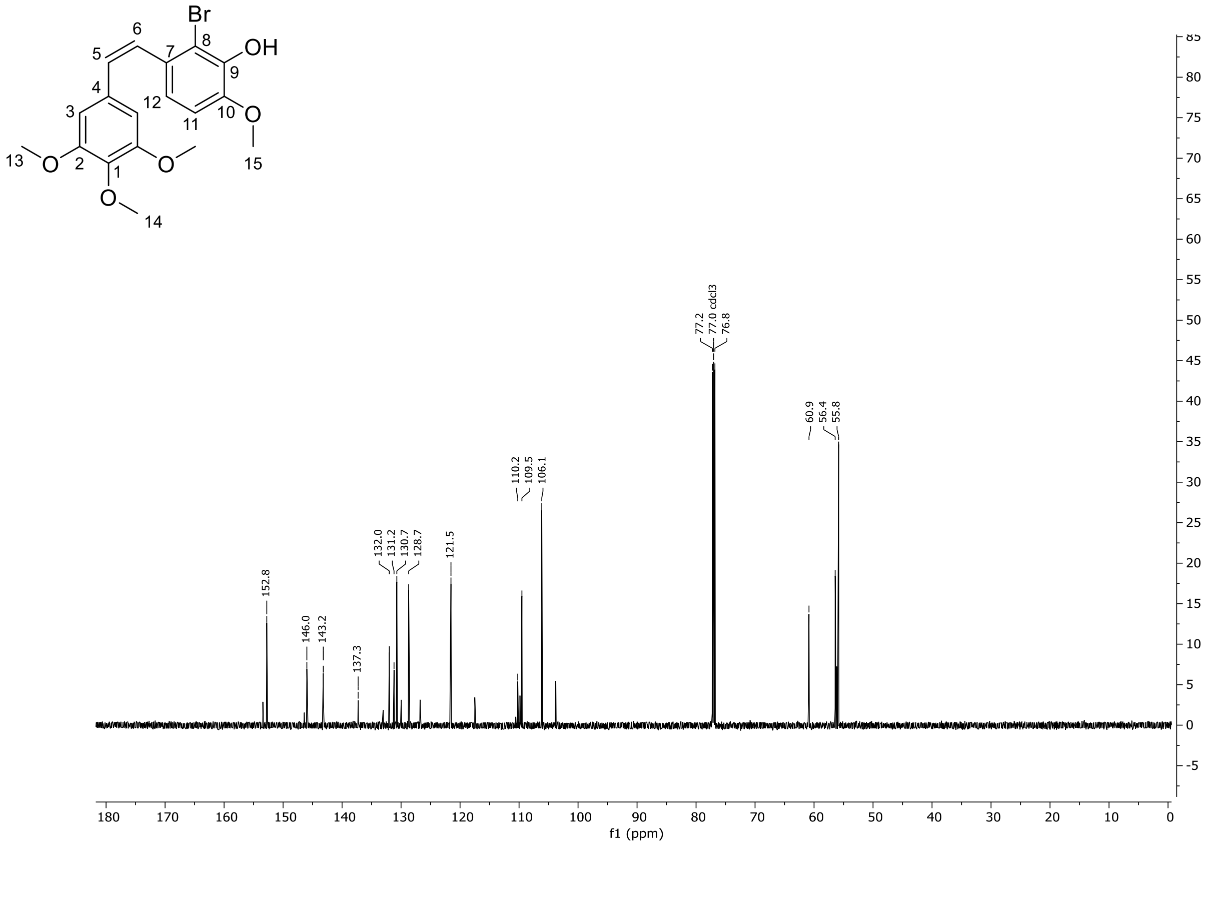

SUPPORTING INFORMATION

¹H-NMR of S15 (400 MHz, $CDCl_3$)

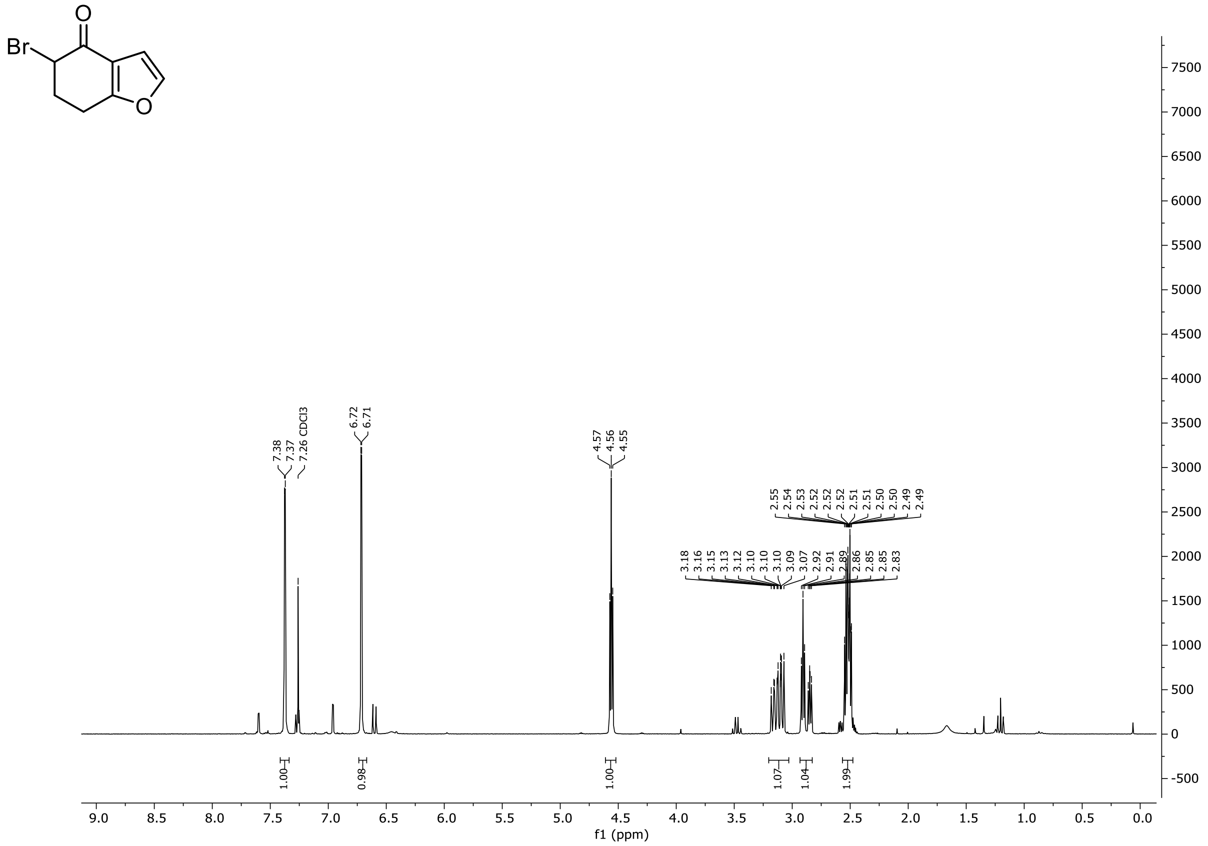

¹H-NMR of S16 (400 MHz, $CDCl_3$)

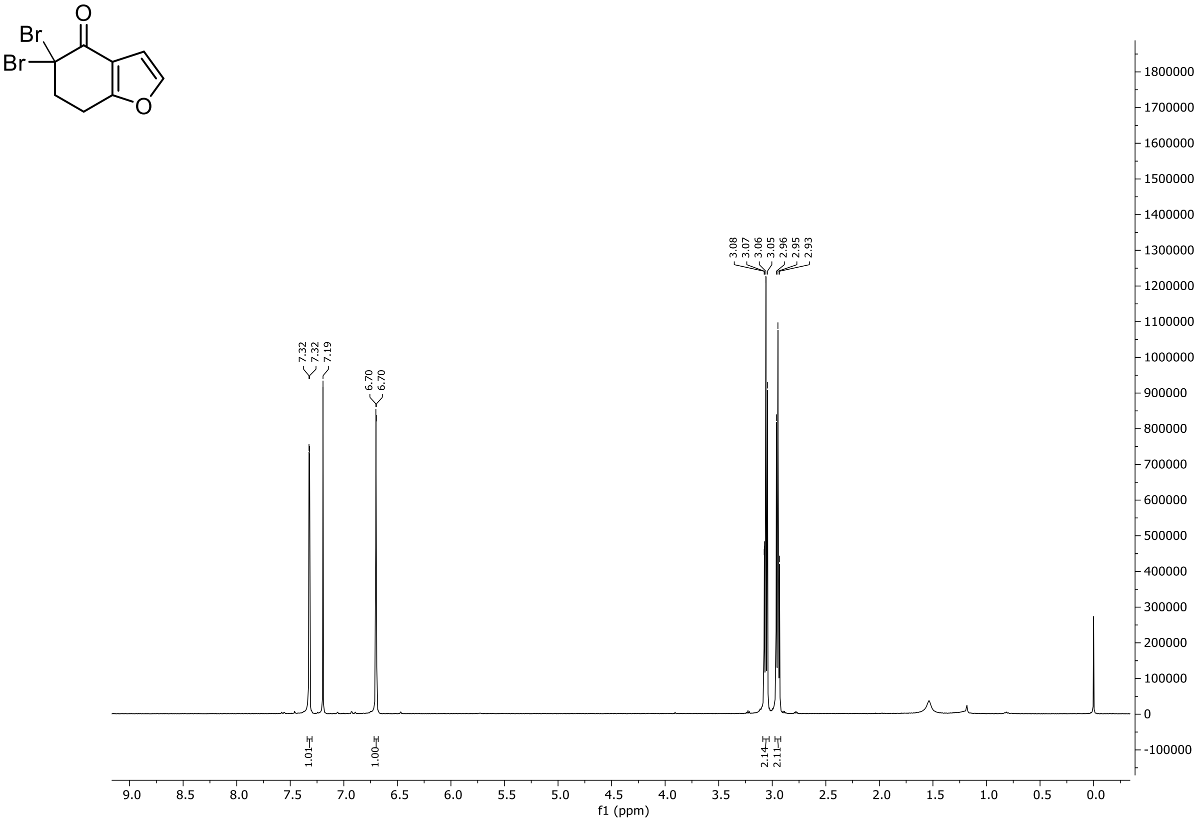

¹H-NMR of S17 (500 MHz, $CDCl_3$)

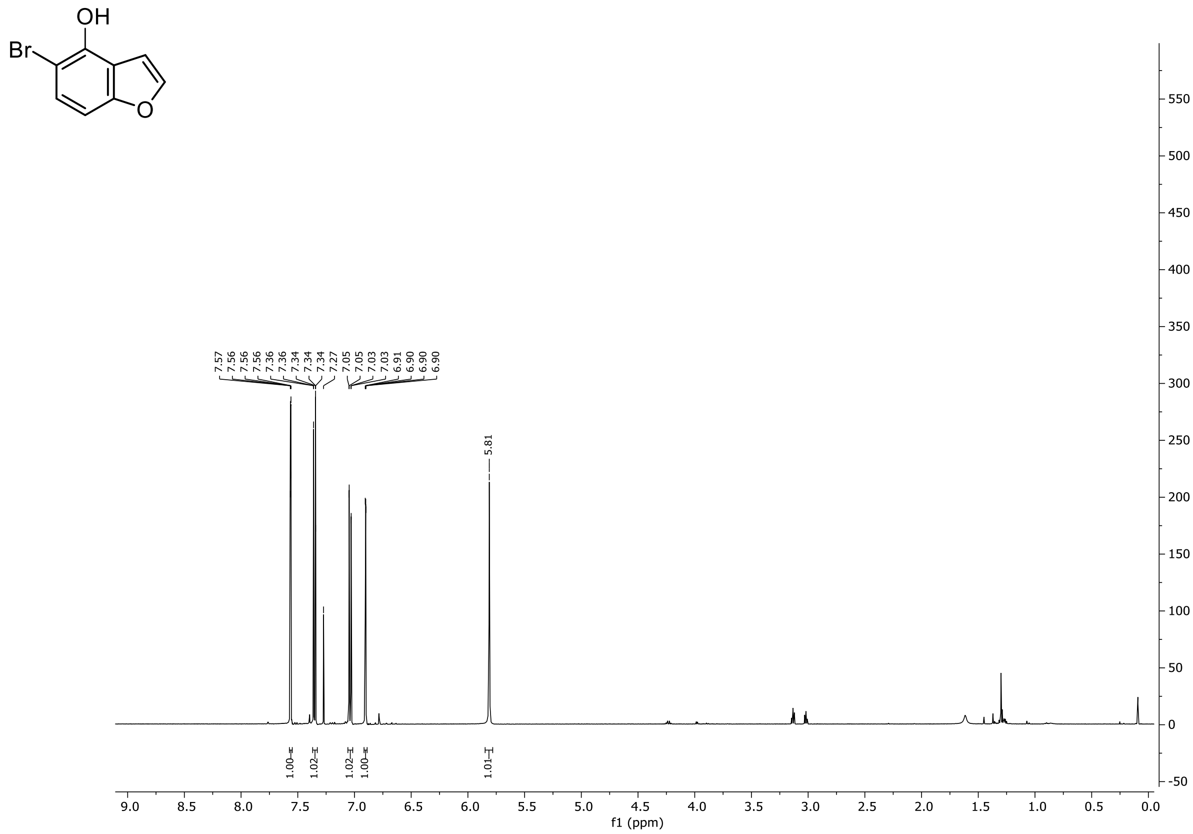

¹³C-NMR of S17 (126 MHz, $CDCl_3$)

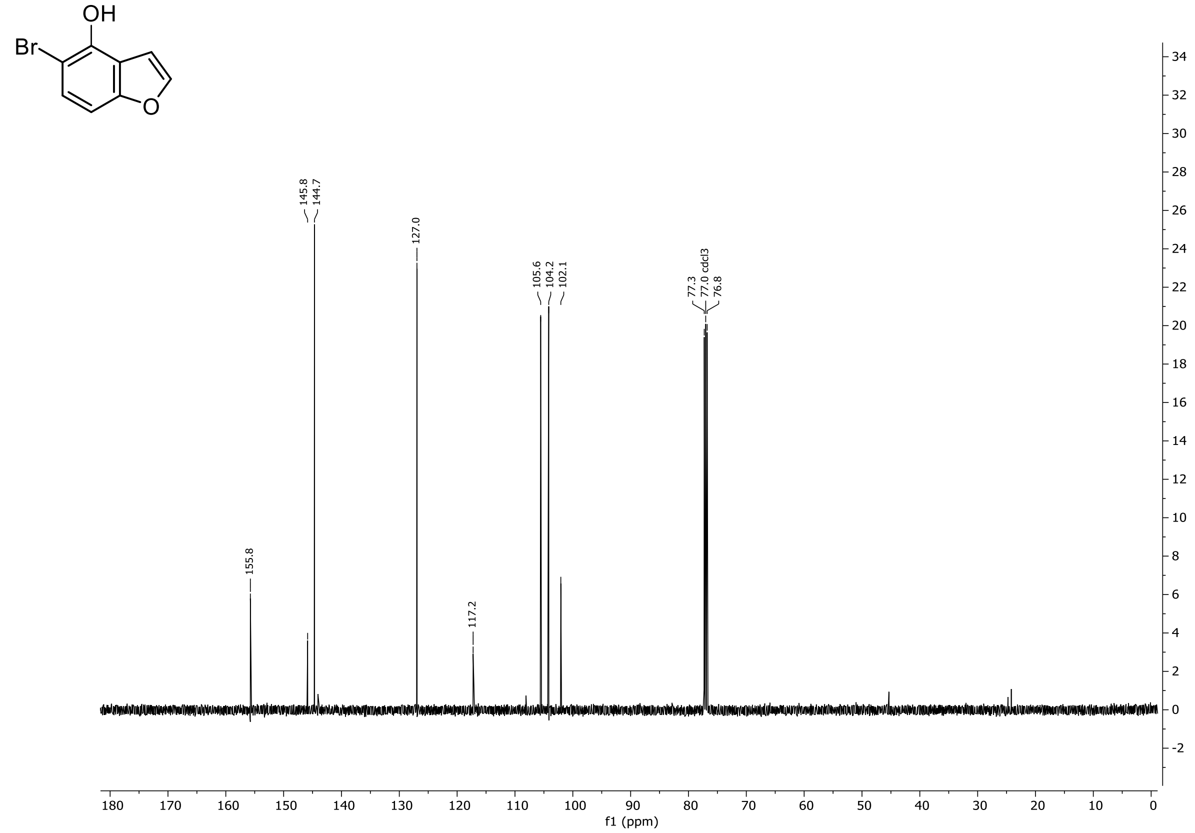

^{1}H-NMR of S18 (300MHz, $CDCl_3$)

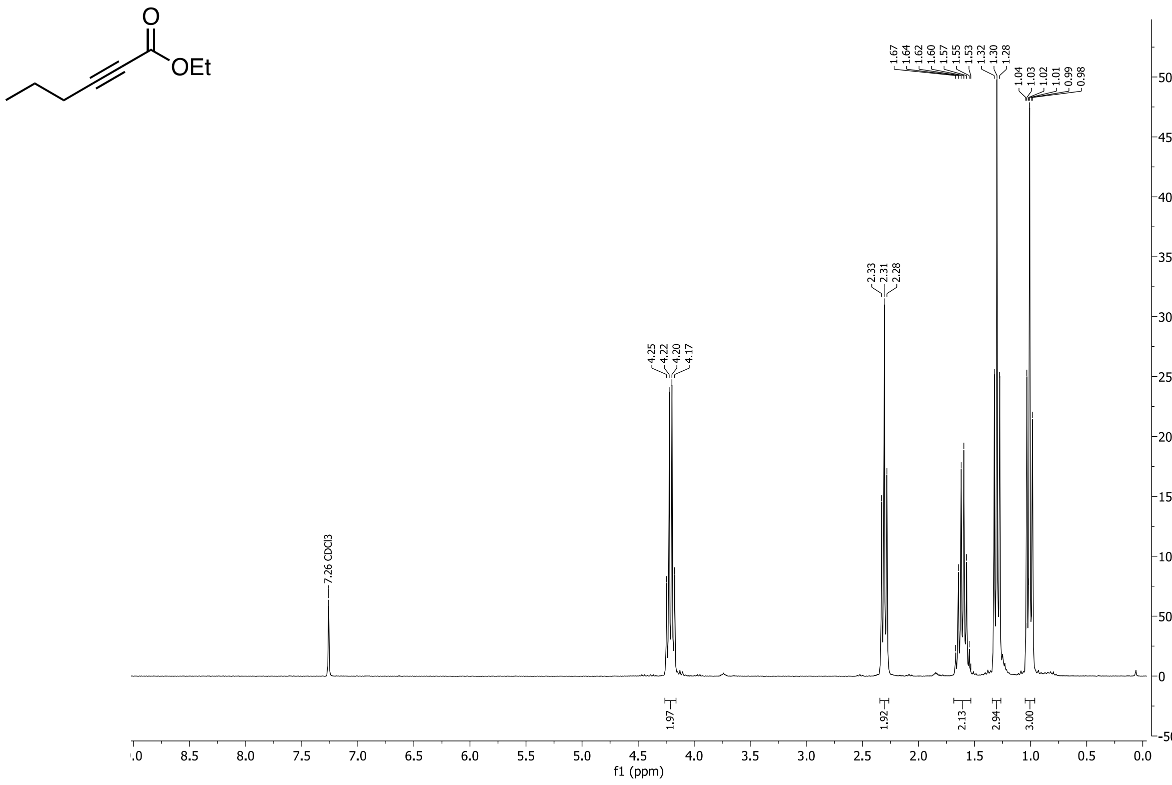

^{1}H-NMR of S19 (300MHz, $CDCl_3$)

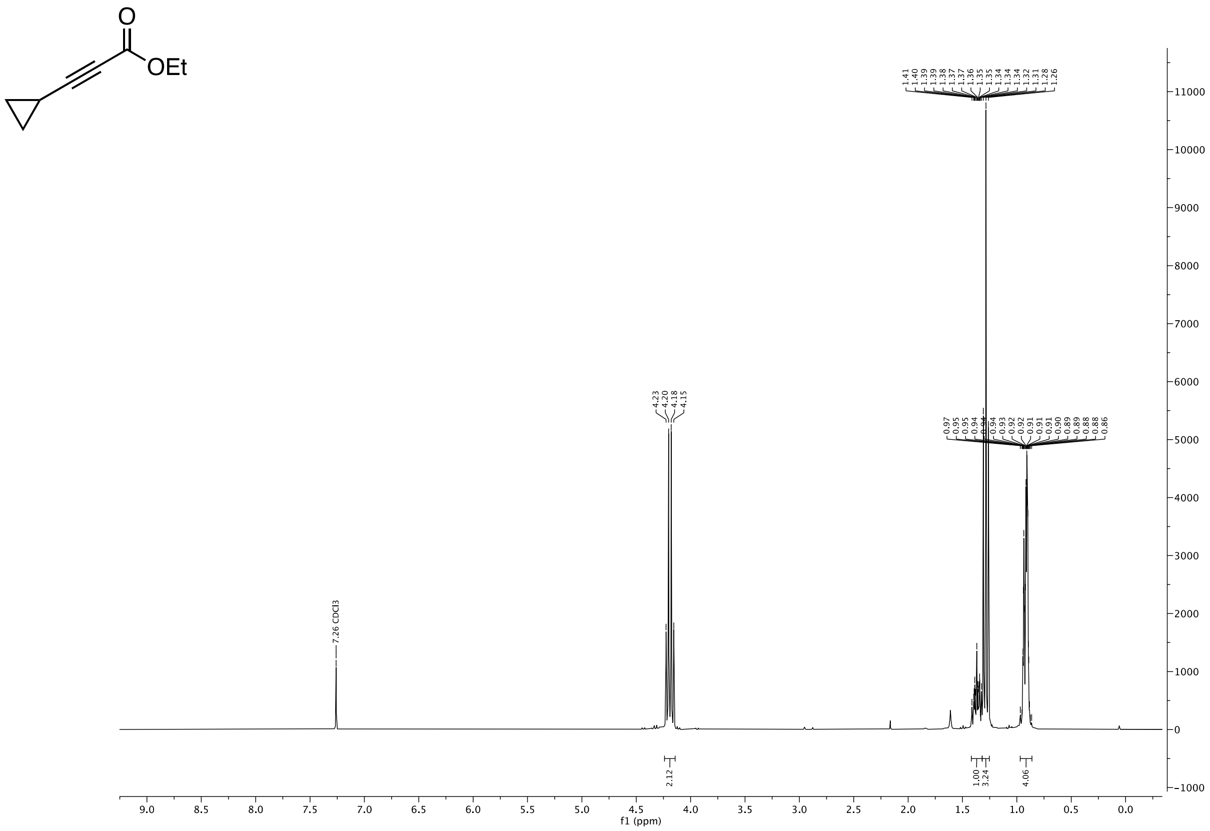

¹H-NMR of S20 (300MHz, $CDCl_3$)

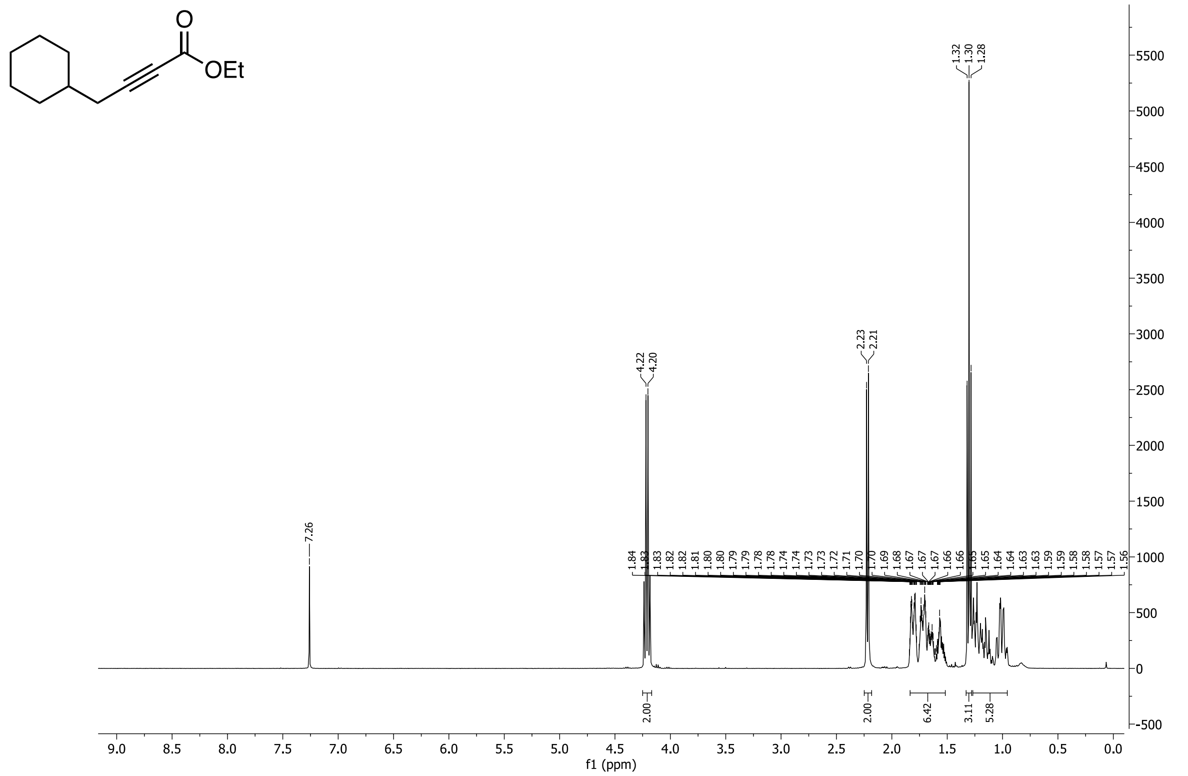

¹H-NMR of *Z*-2 (300MHz, $CDCl_3$)

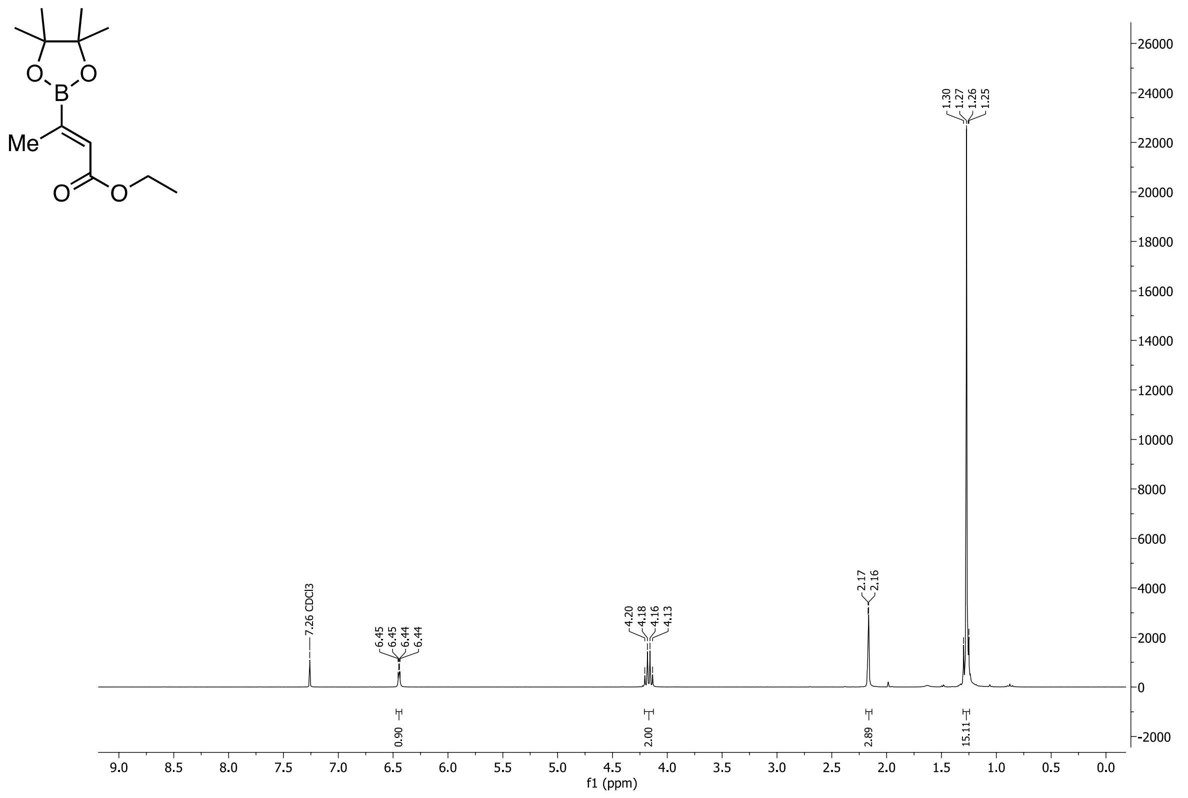

^{1}H-NMR of S21 (300MHz, $CDCl_3$)

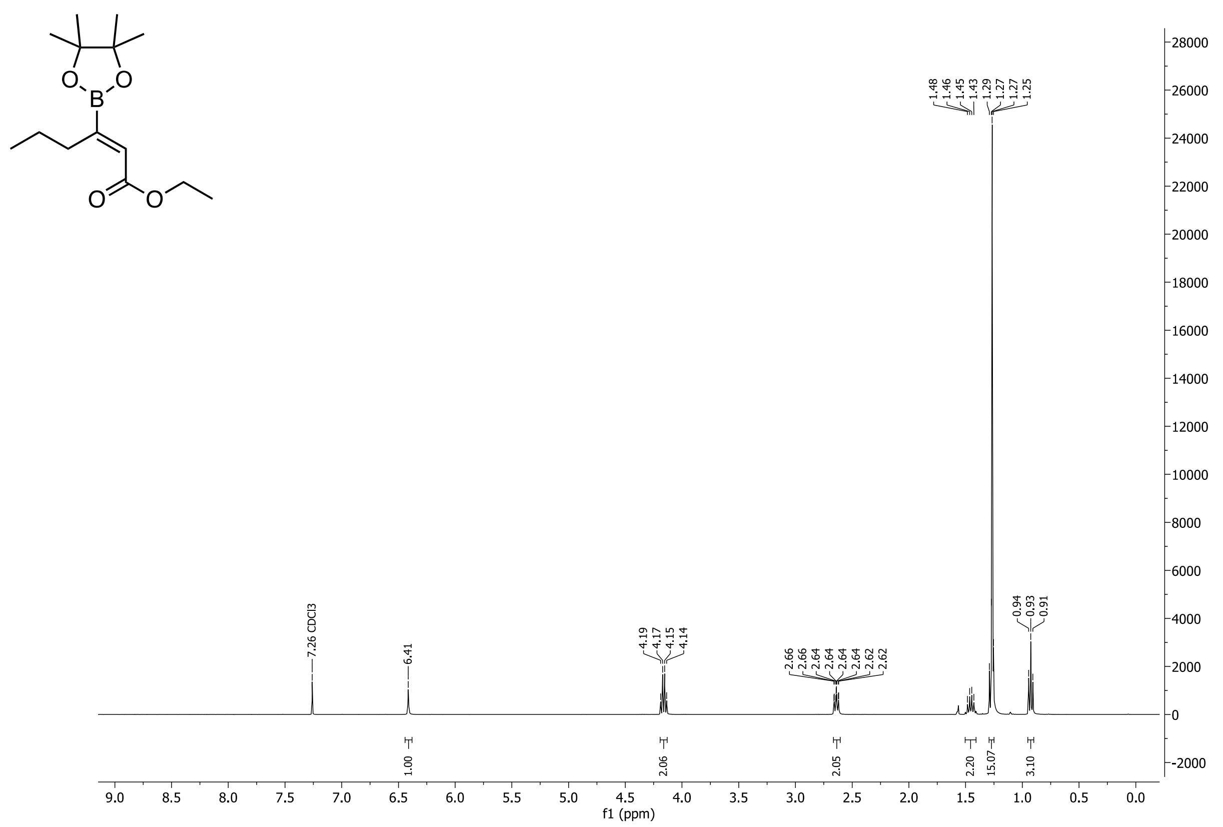

^{1}H-NMR of S22 (599 MHz, $CDCl_3$)

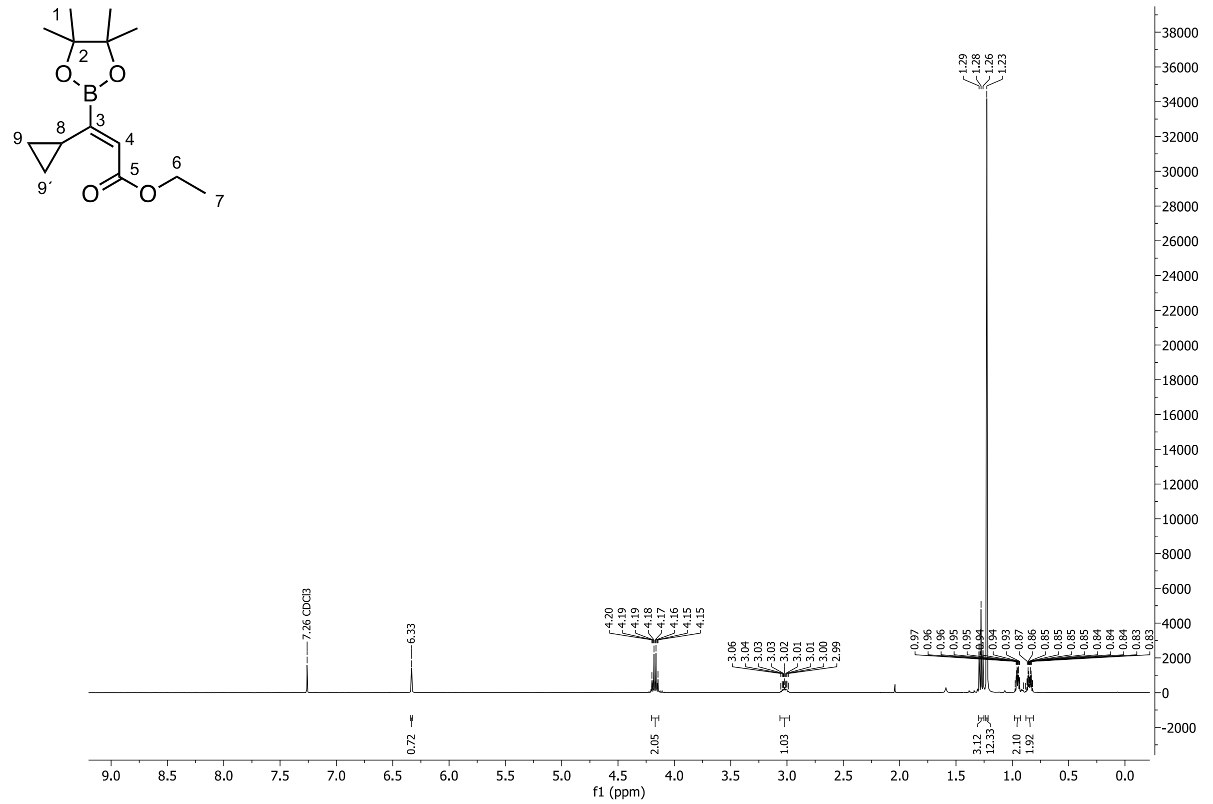

^{13}C-NMR of S22 (151 MHz, $CDCl_3$)

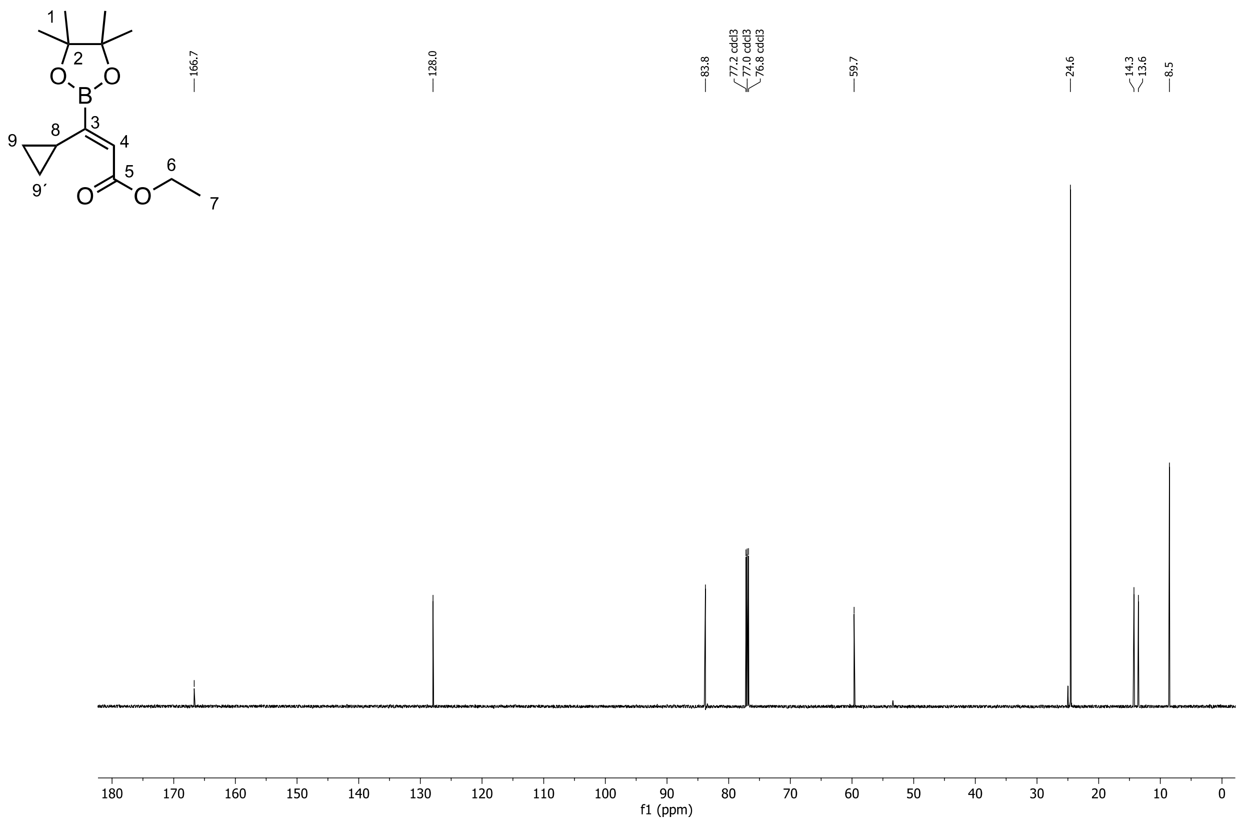

^{1}H-NMR of S23 (500MHz, $CDCl_3$)

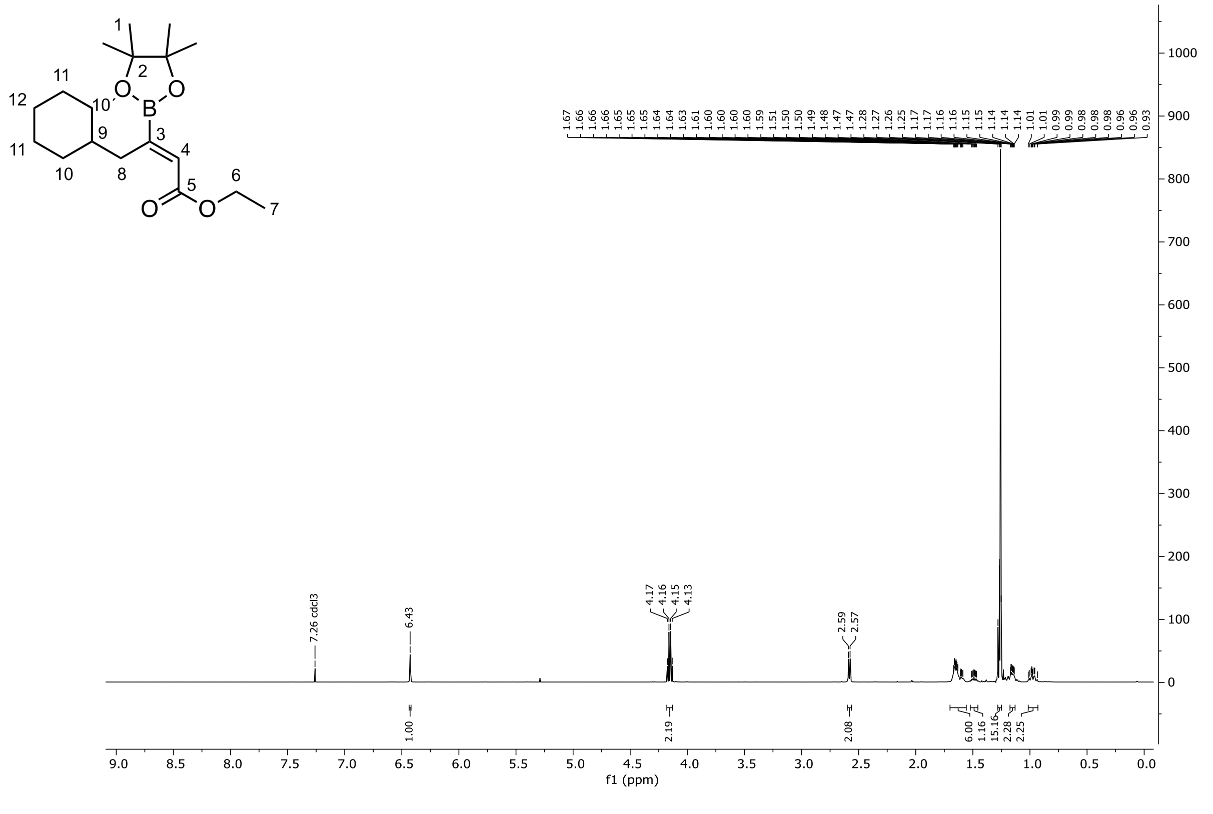

^{13}C-NMR of S23 (126 MHz, $CDCl_3$)

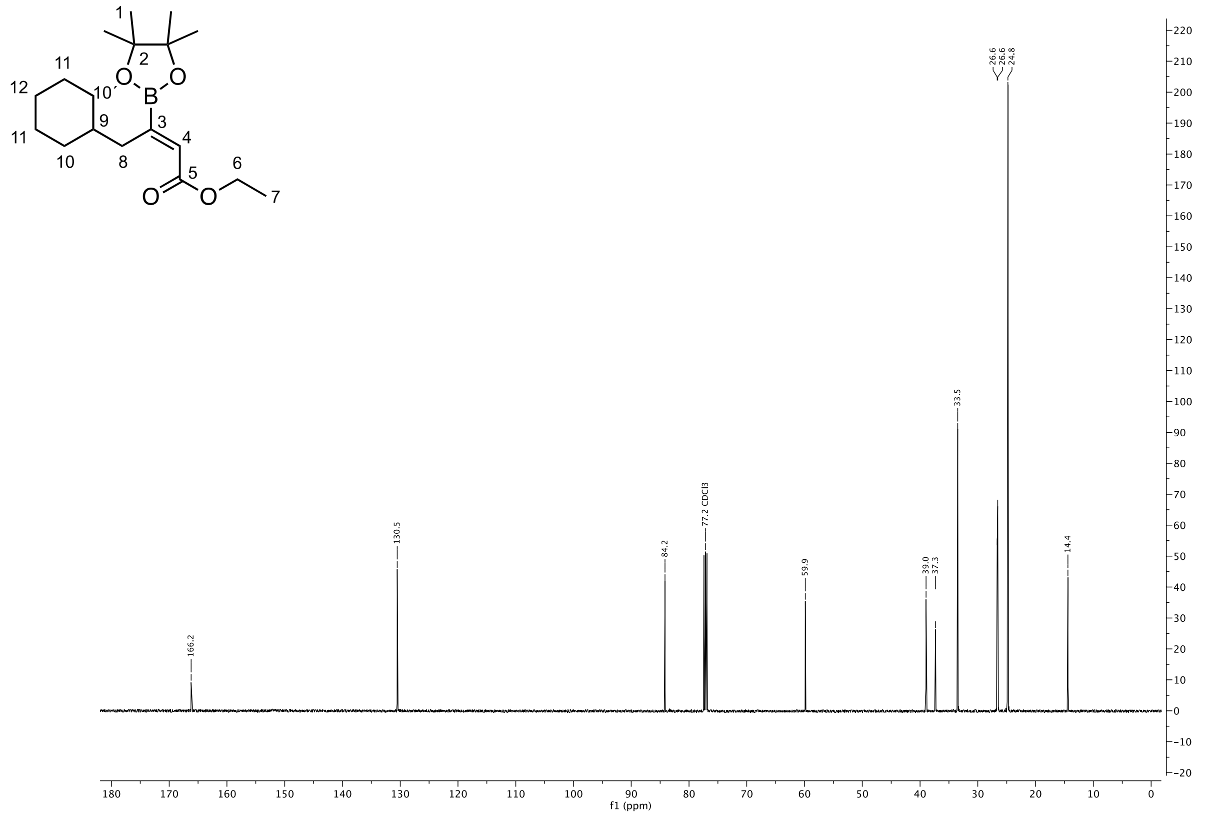

^{1}H-NMR of S24 (500MHz, $CDCl_3$)

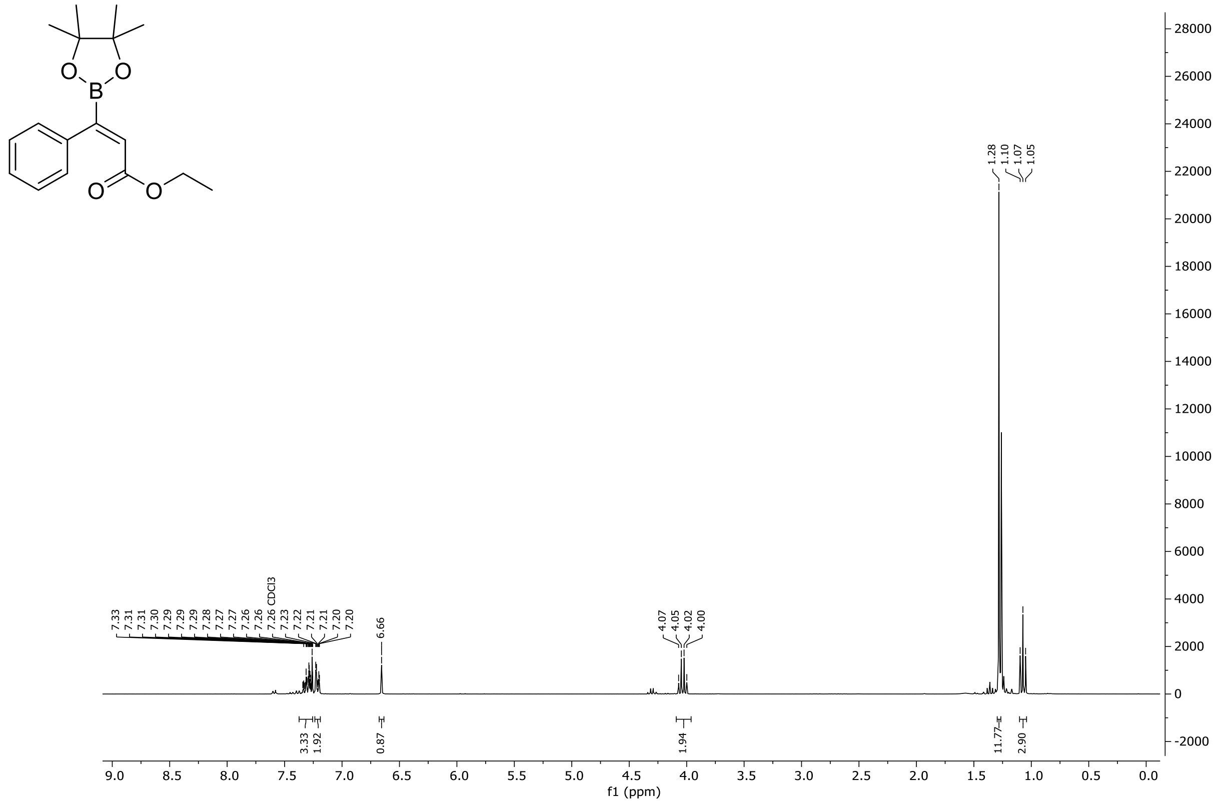

¹H-NMR of S25 (400MHz, $CDCl_3$)

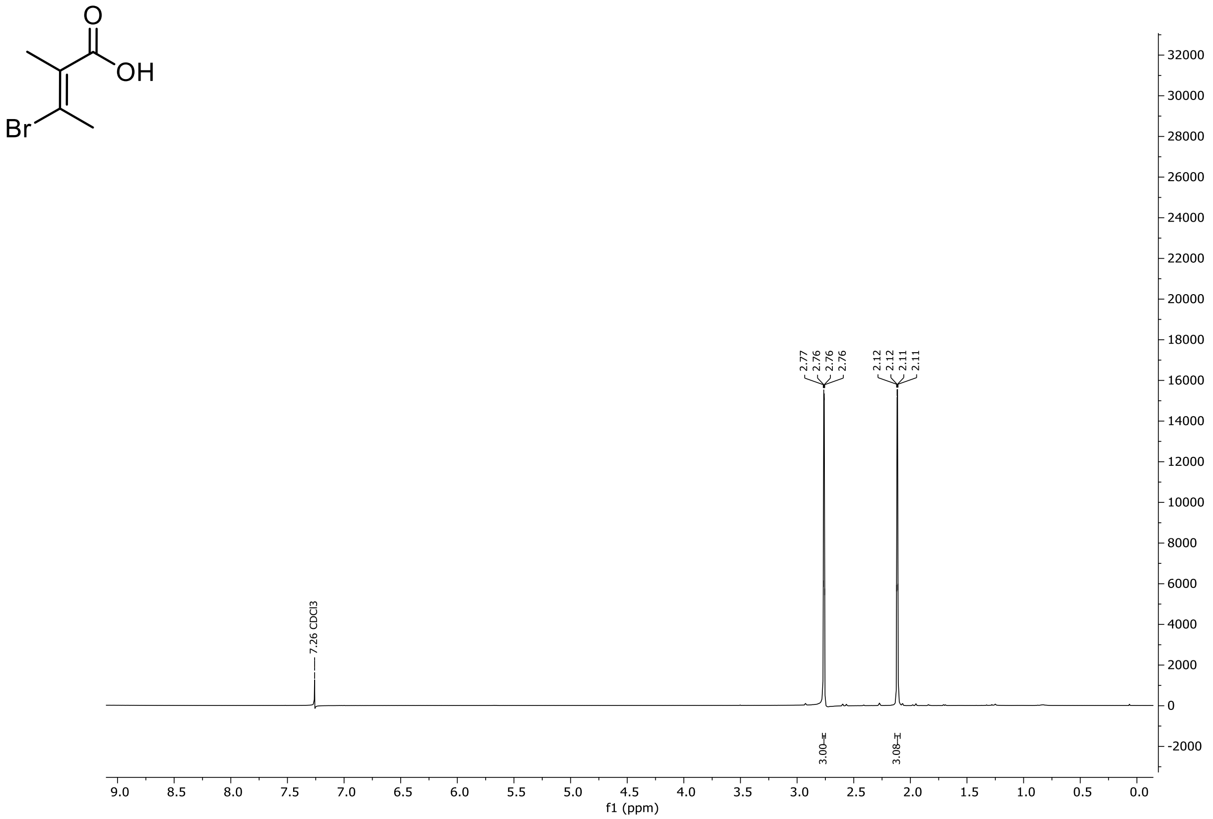

¹H-NMR of S26 (500MHz, $CDCl_3$)

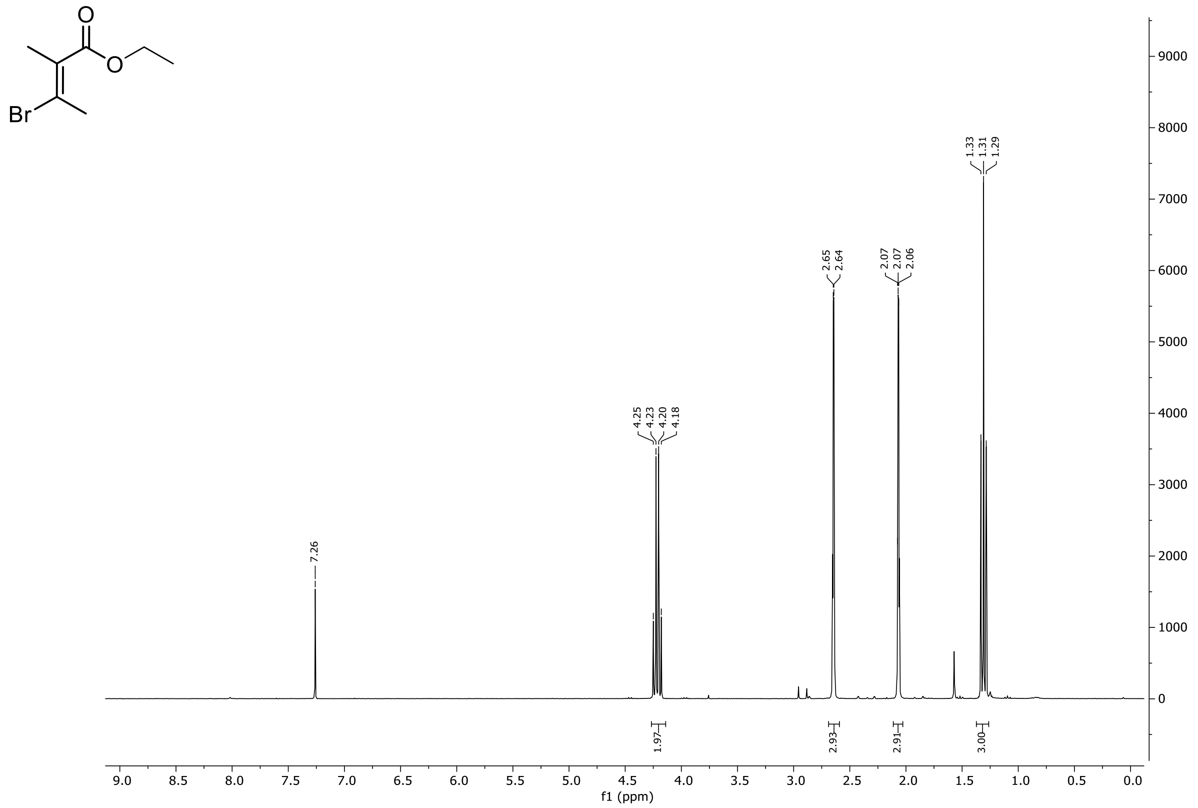

SUPPORTING INFORMATION

^{13}C-NMR of S26 (500MHz, $CDCl_3$)

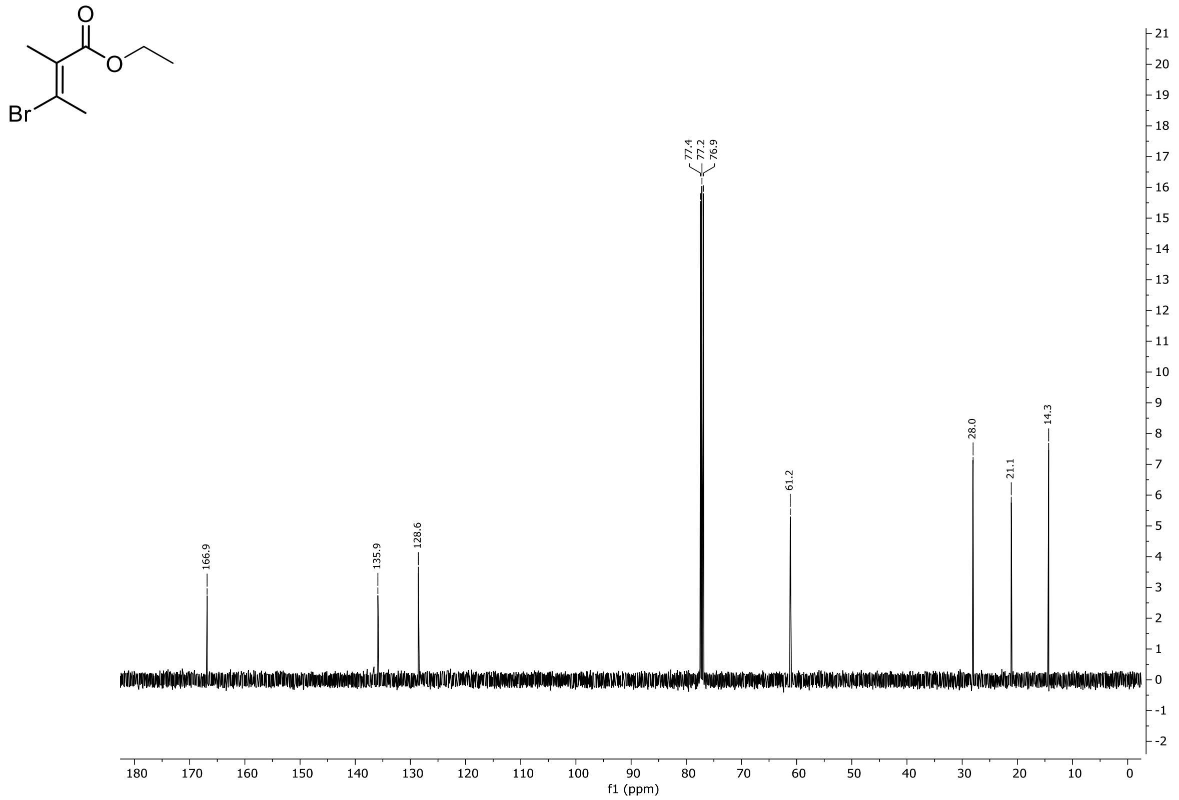

^{1}H-NMR of S27 (500MHz, $CDCl_3$)

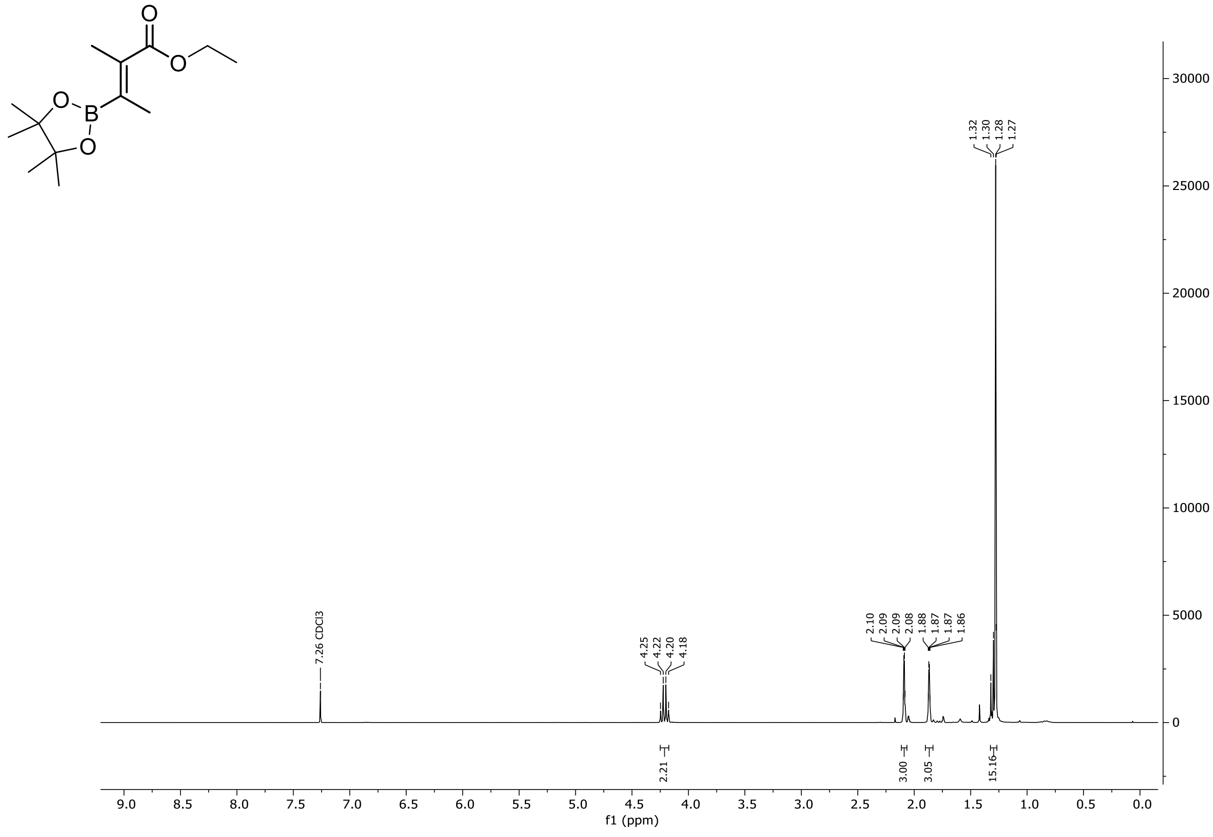

^{13}C-NMR of S27 (500MHz, $CDCl_3$)

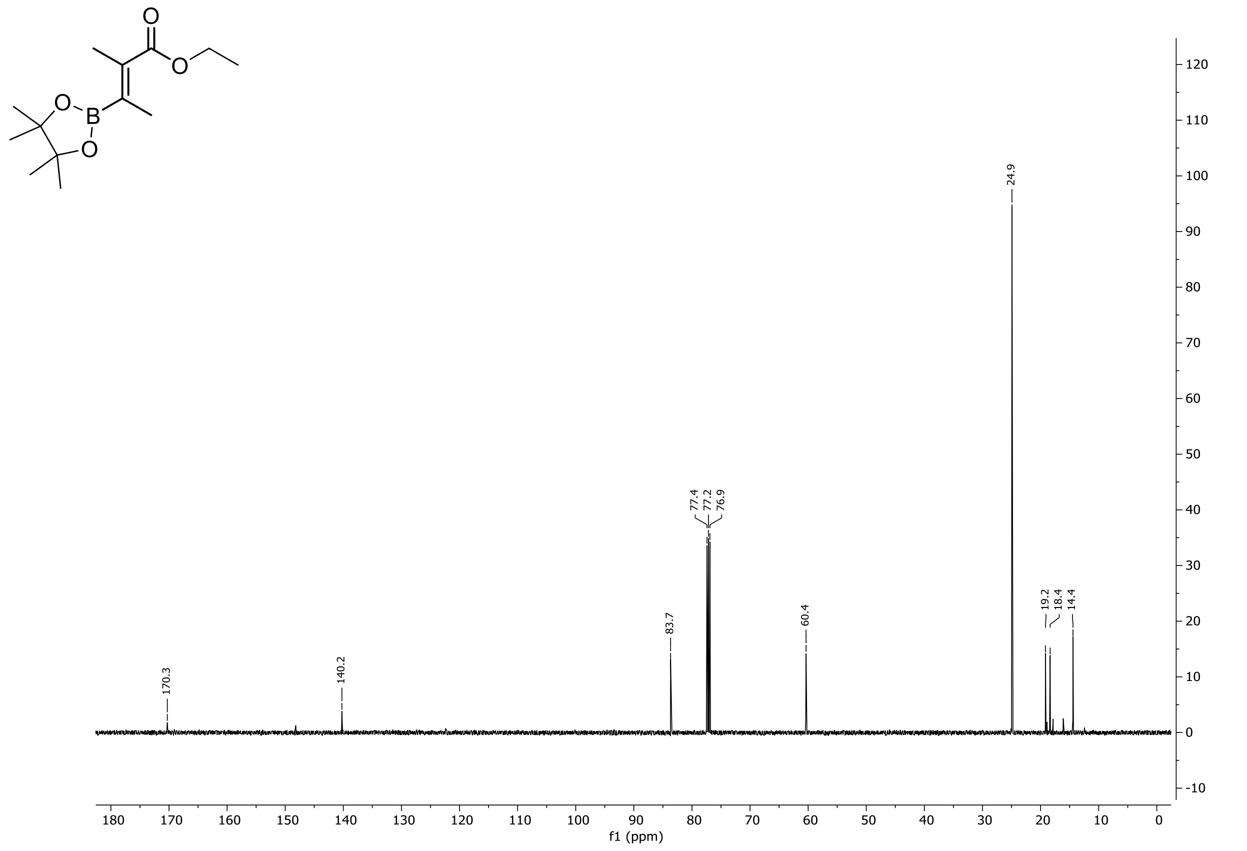

^{1}H-NMR of *E*-20 (300MHz, $CDCl_3$)

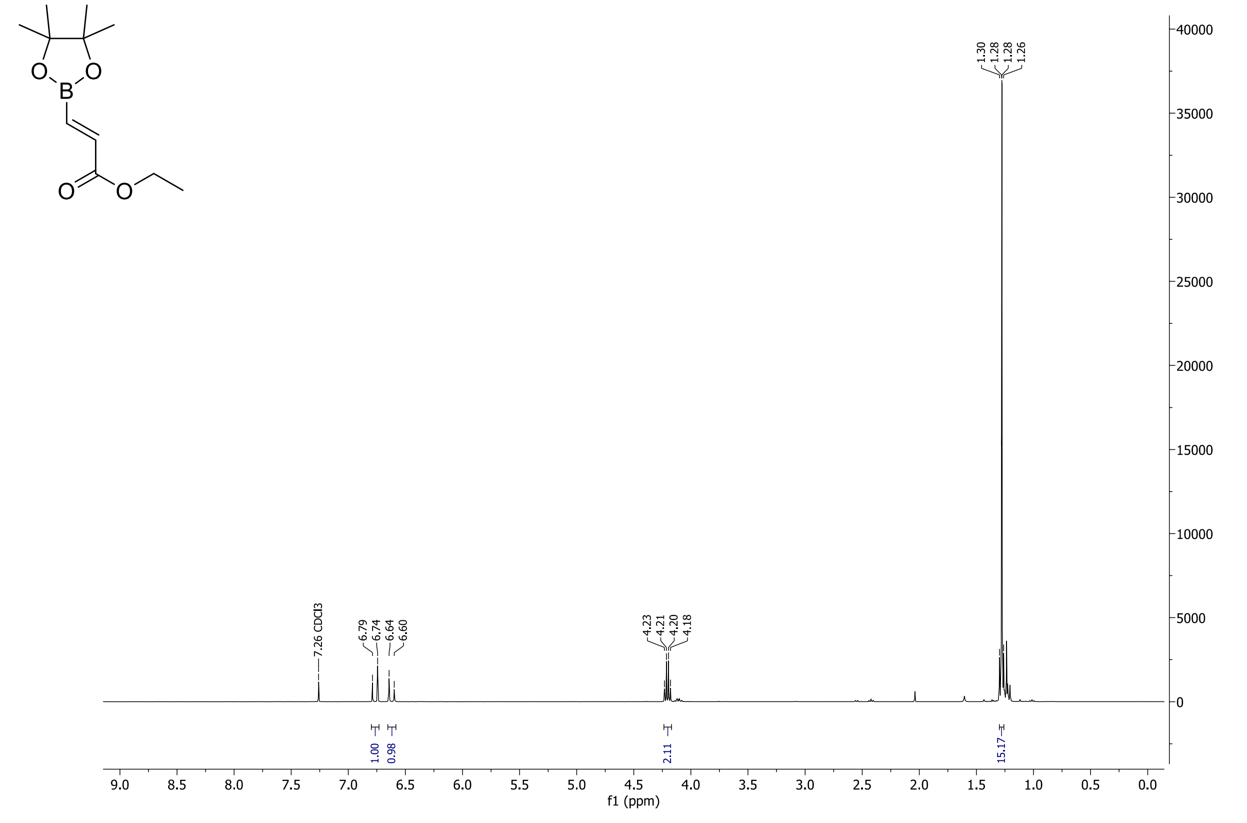

^{1}H-NMR of S28 (500 MHz, $CDCl_3$)

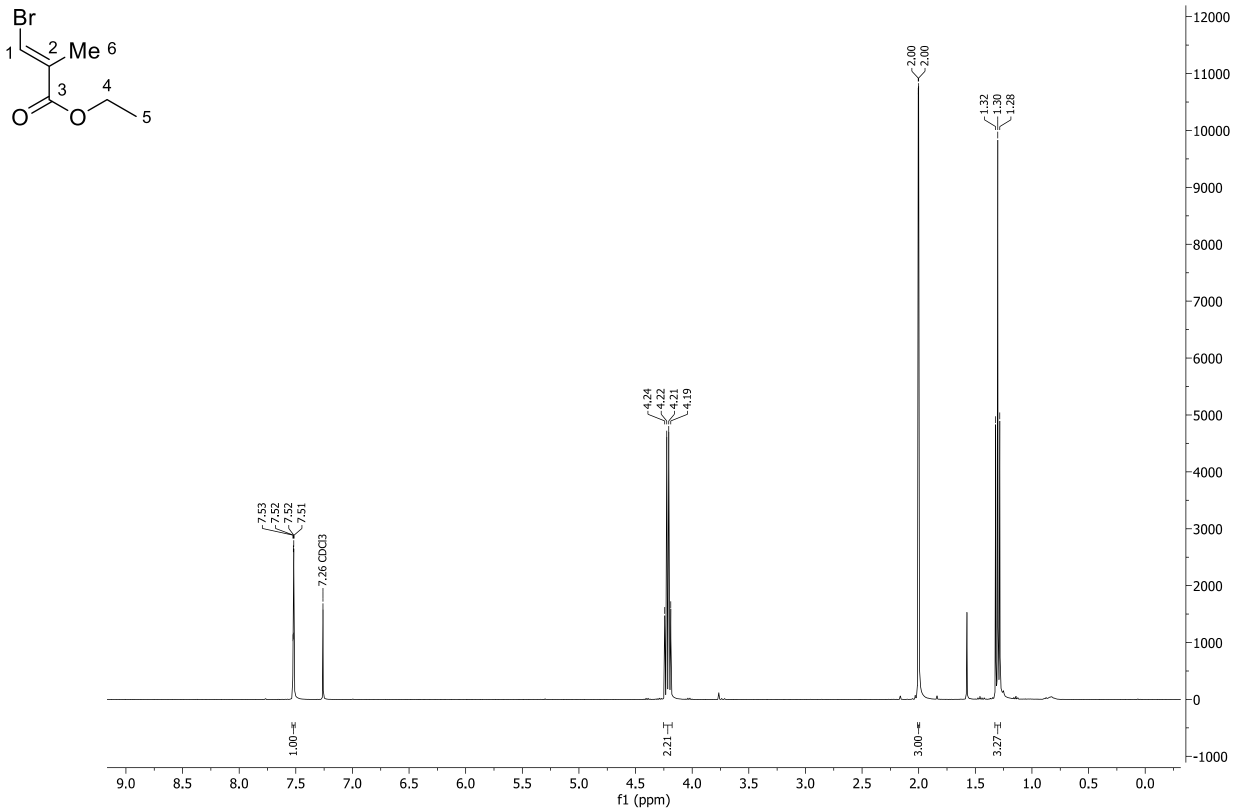

^{13}C-NMR of S28 (101 MHz, $CDCl_3$)

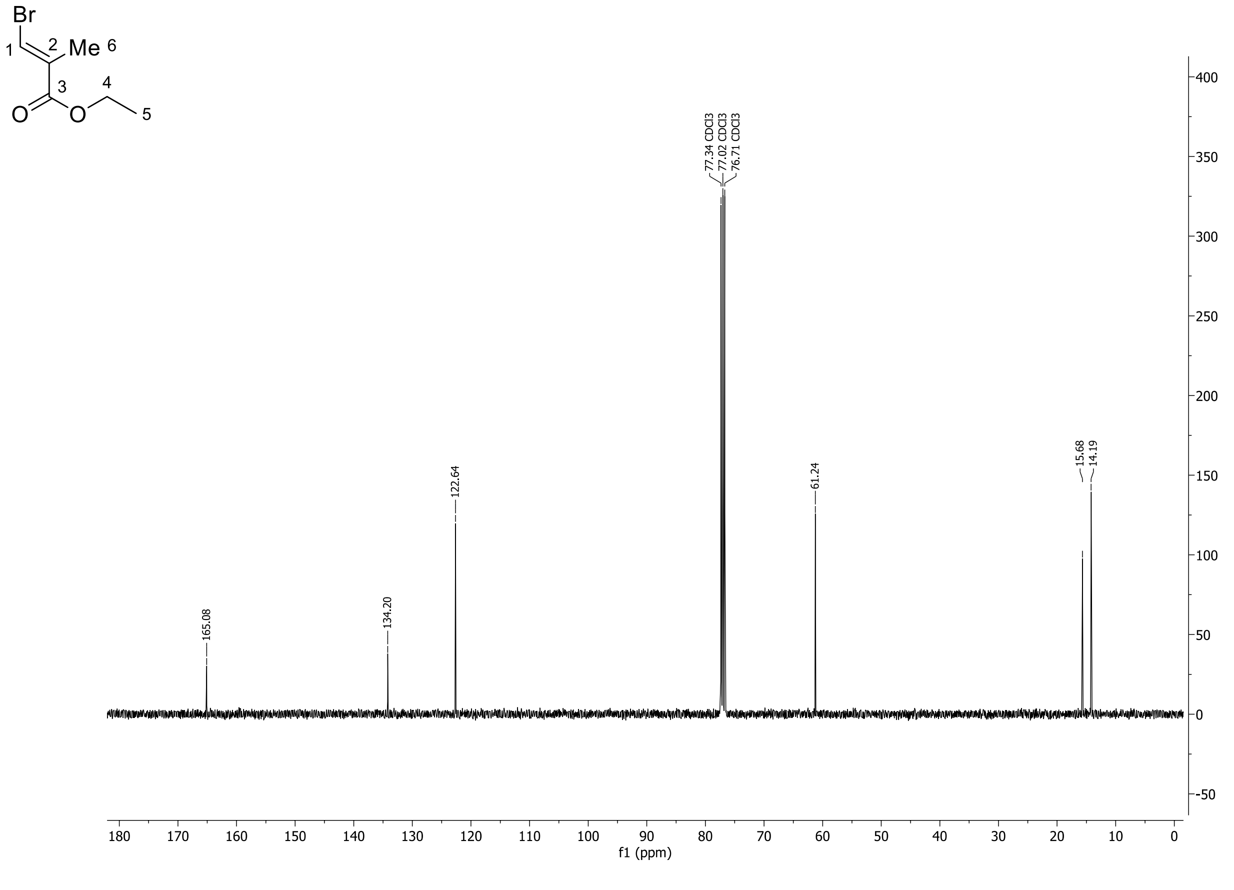

^{1}H-NMR of *E*-21 (599 MHz, $CDCl_3$)

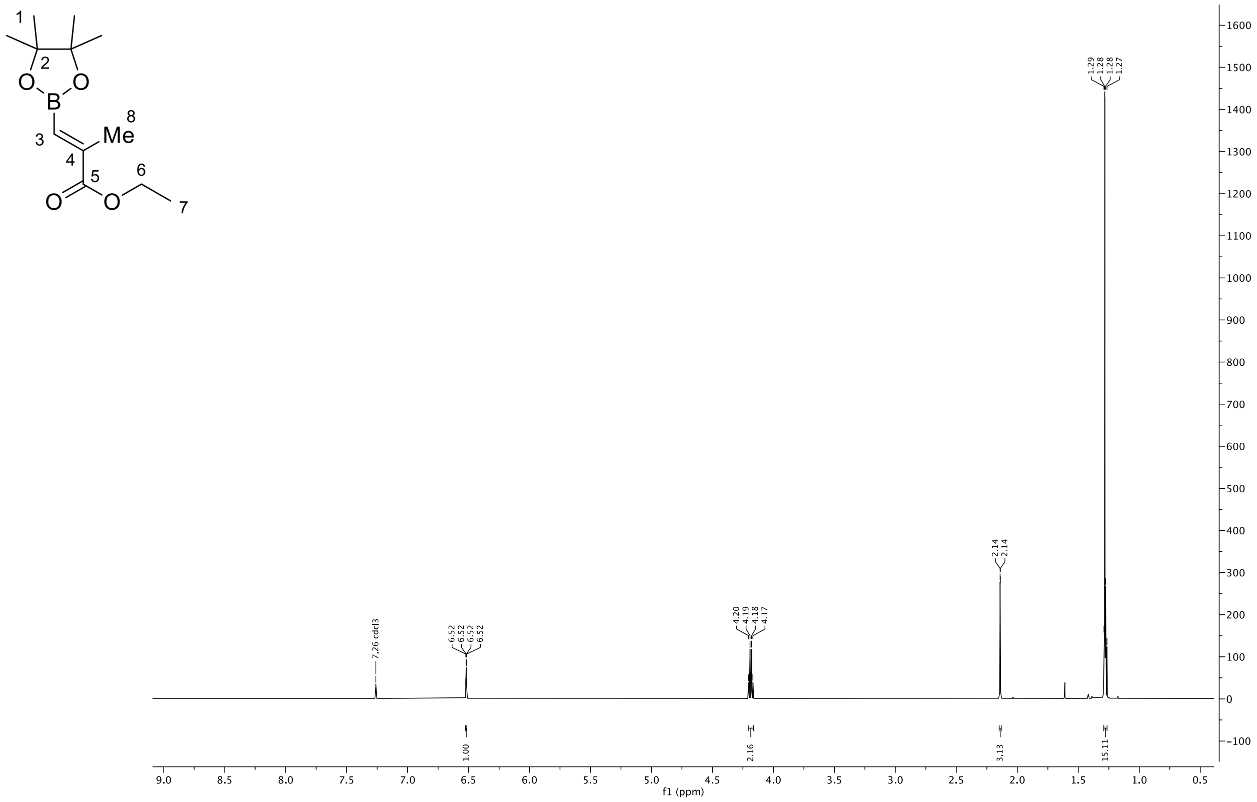

^{13}C-NMR of *E*-21 (151 MHz, $CDCl_3$)

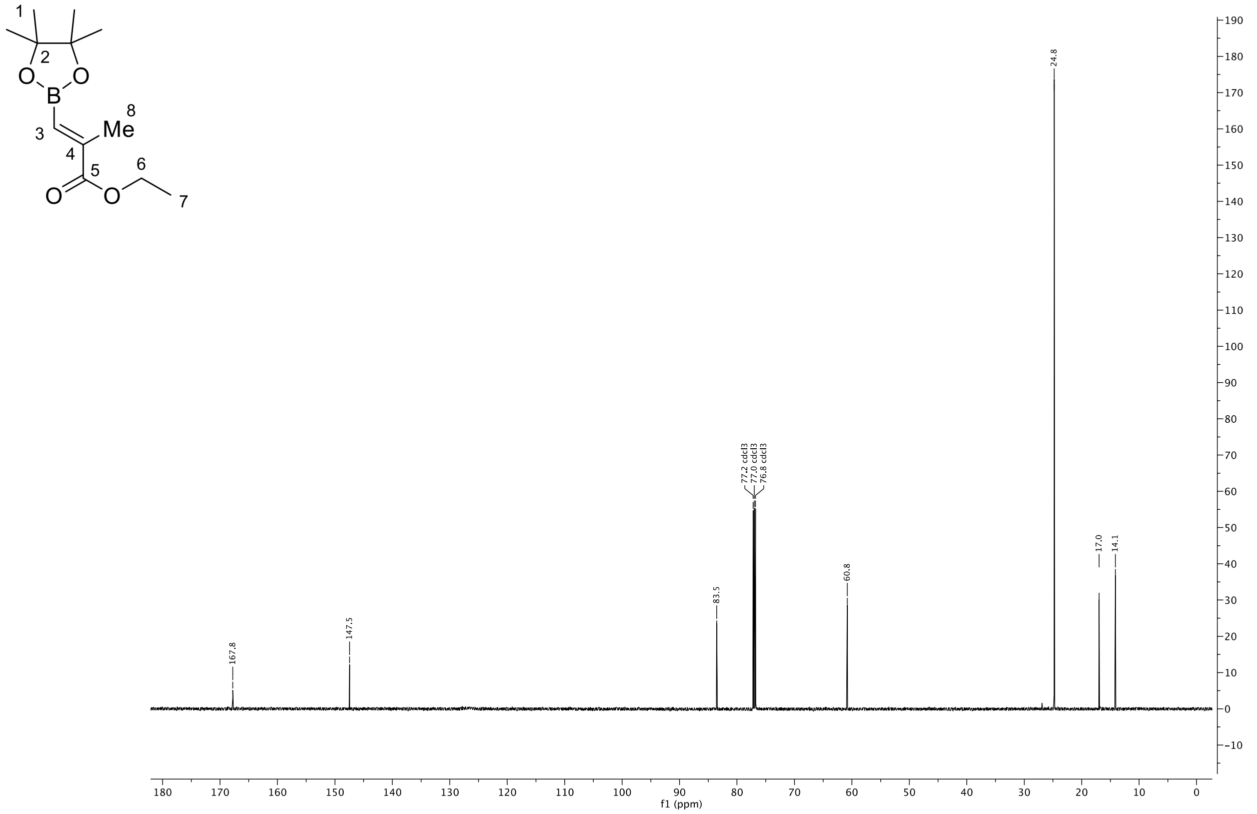

^{1}H-NMR of *Z*-20 (599 MHz, $CDCl_3$)

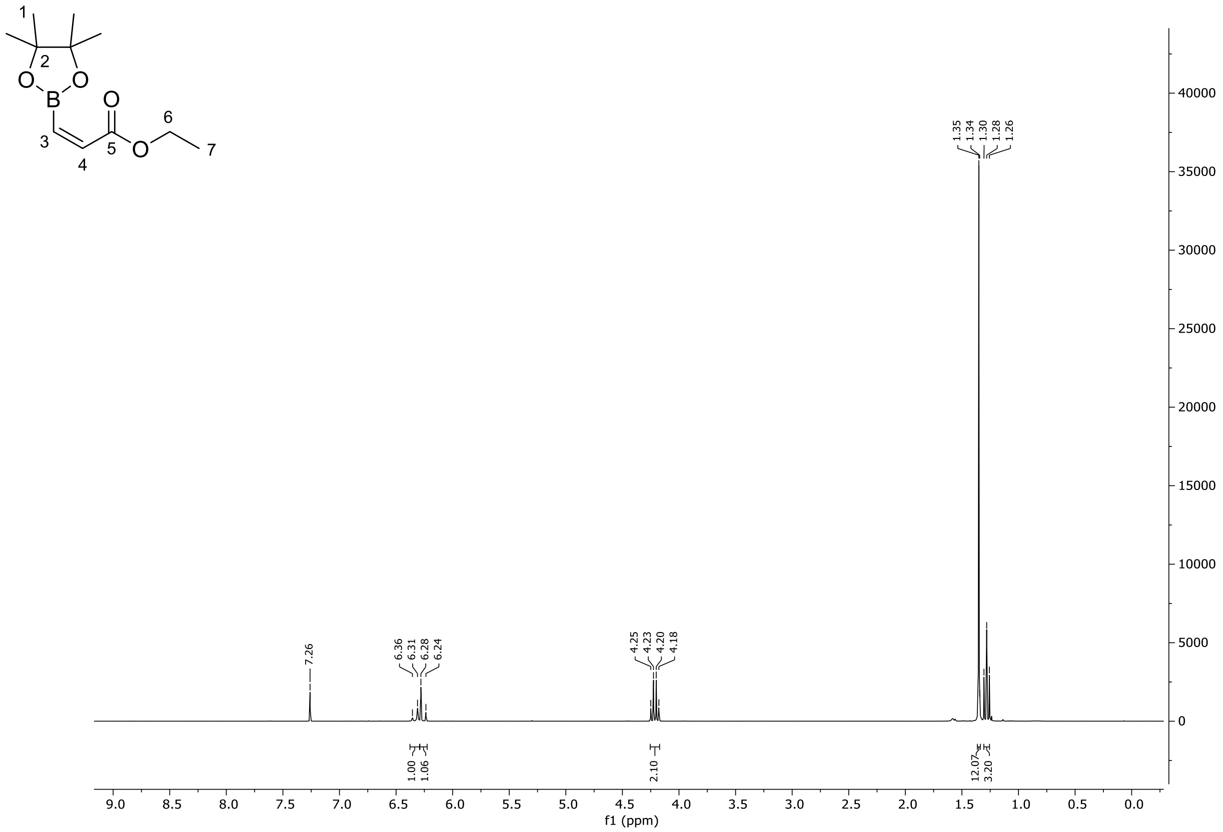

^{13}C-NMR of *Z*-20 (151 MHz, $CDCl_3$)

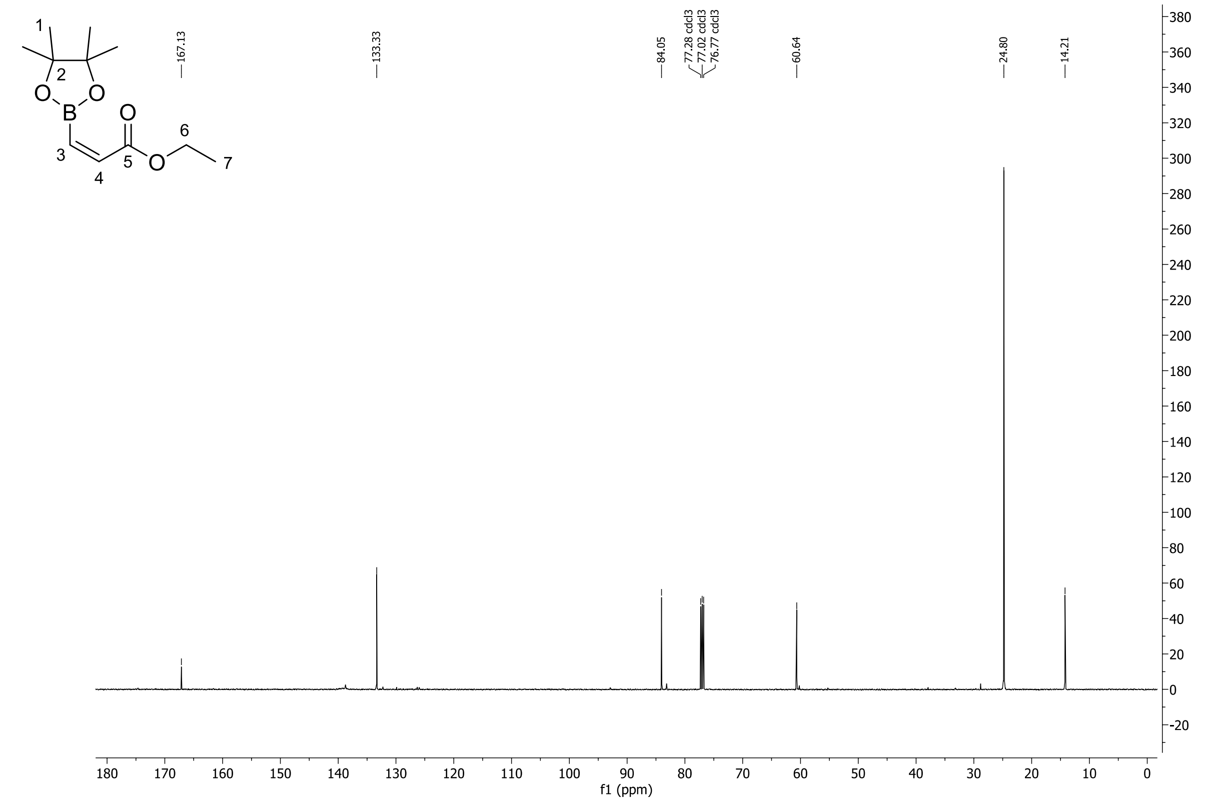

^{1}H-NMR of *Z*-21 (599 MHz, $CDCl_3$)

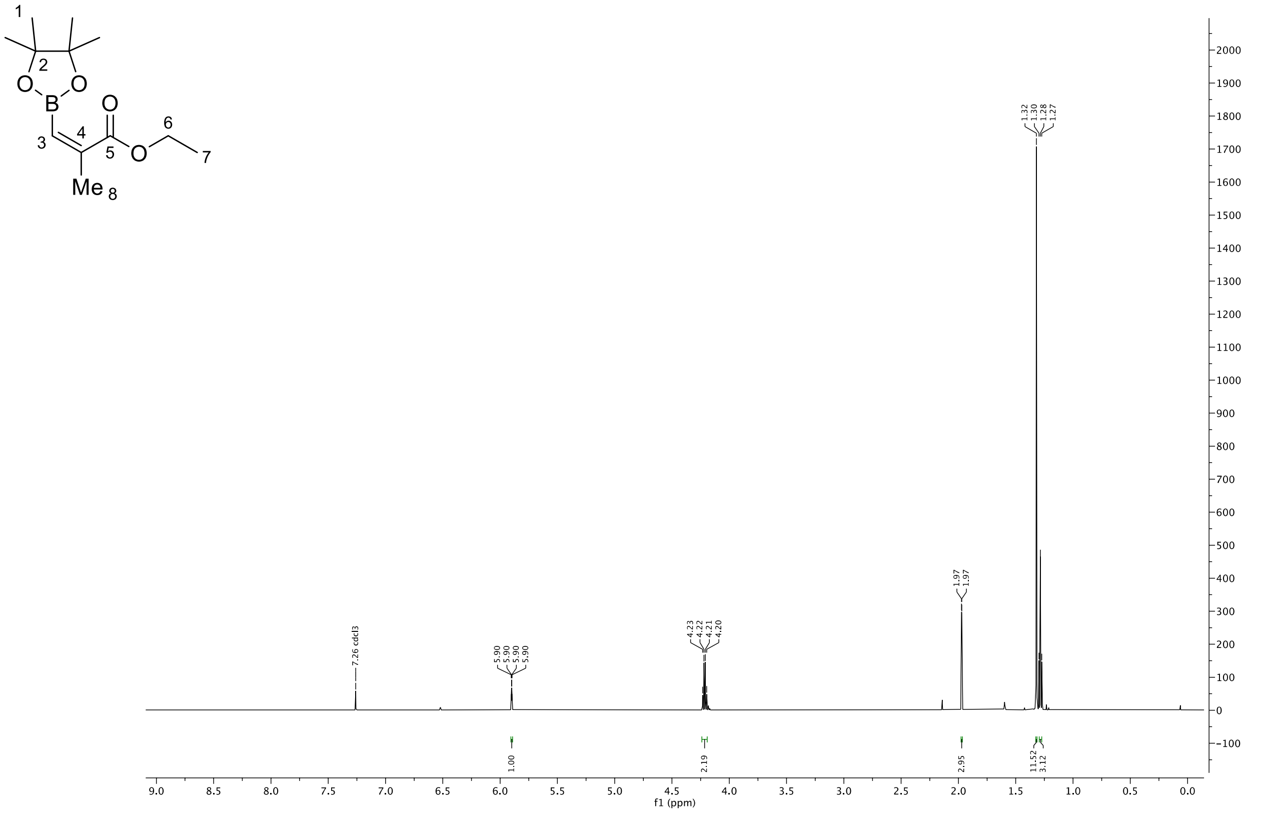

^{13}C-NMR of *Z*-21 (151 MHz, $CDCl_3$)

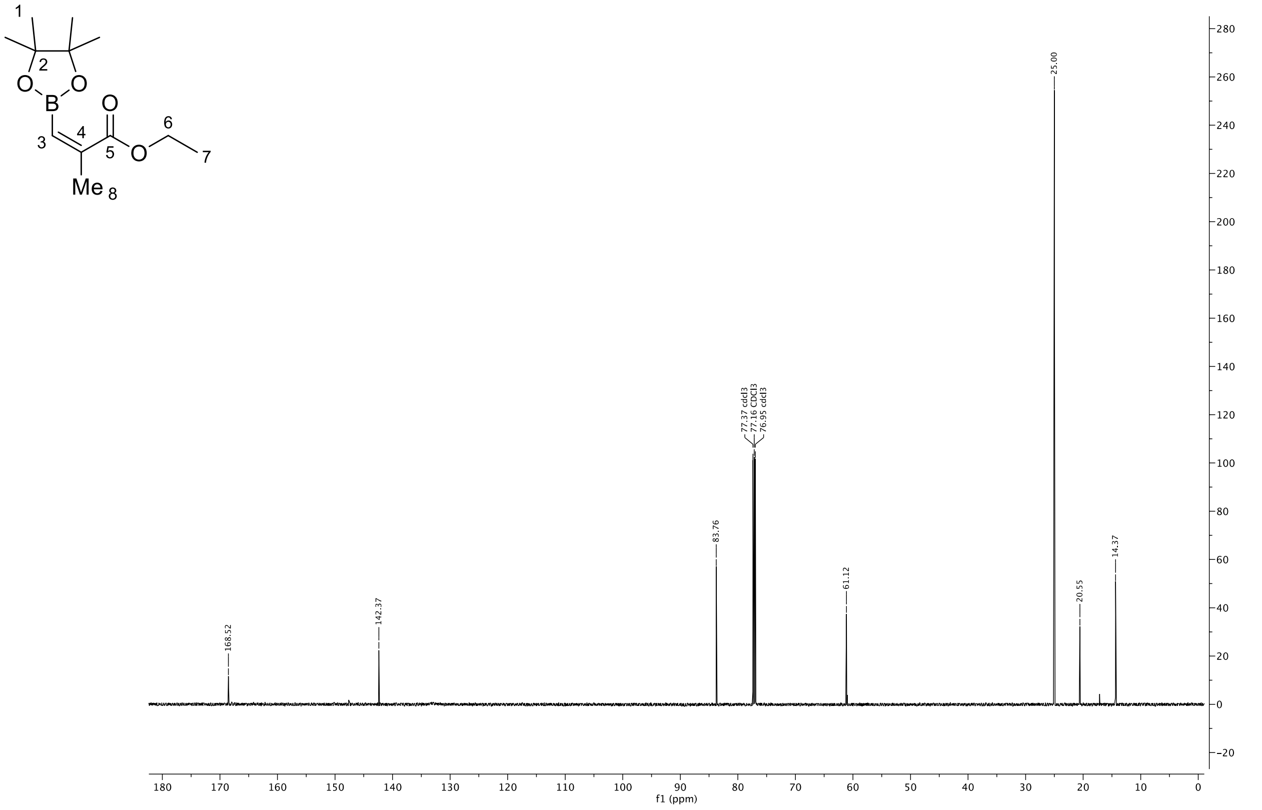

SUPPORTING INFORMATION

¹H-NMR of 3 (300 MHz, $CDCl_3$)

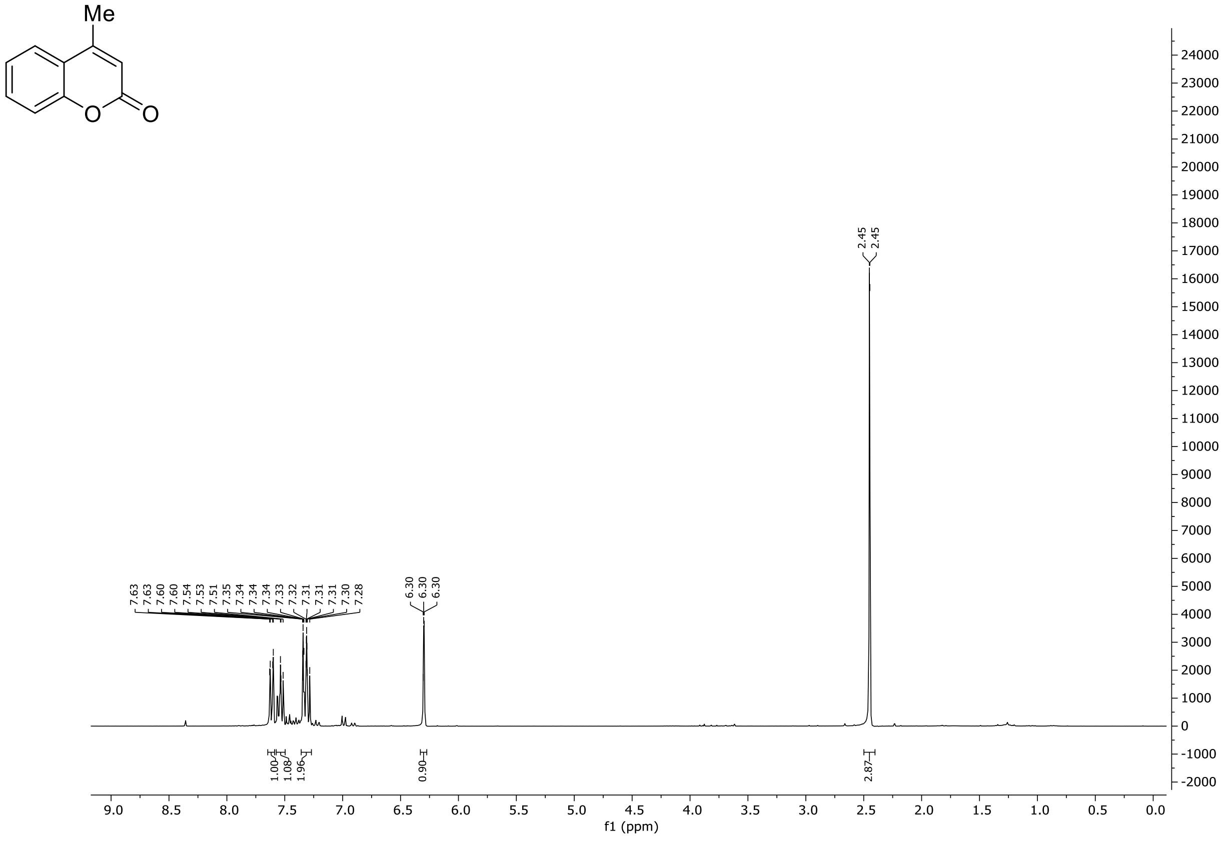

¹H-NMR of 4 (400 MHz, $CDCl_3$)

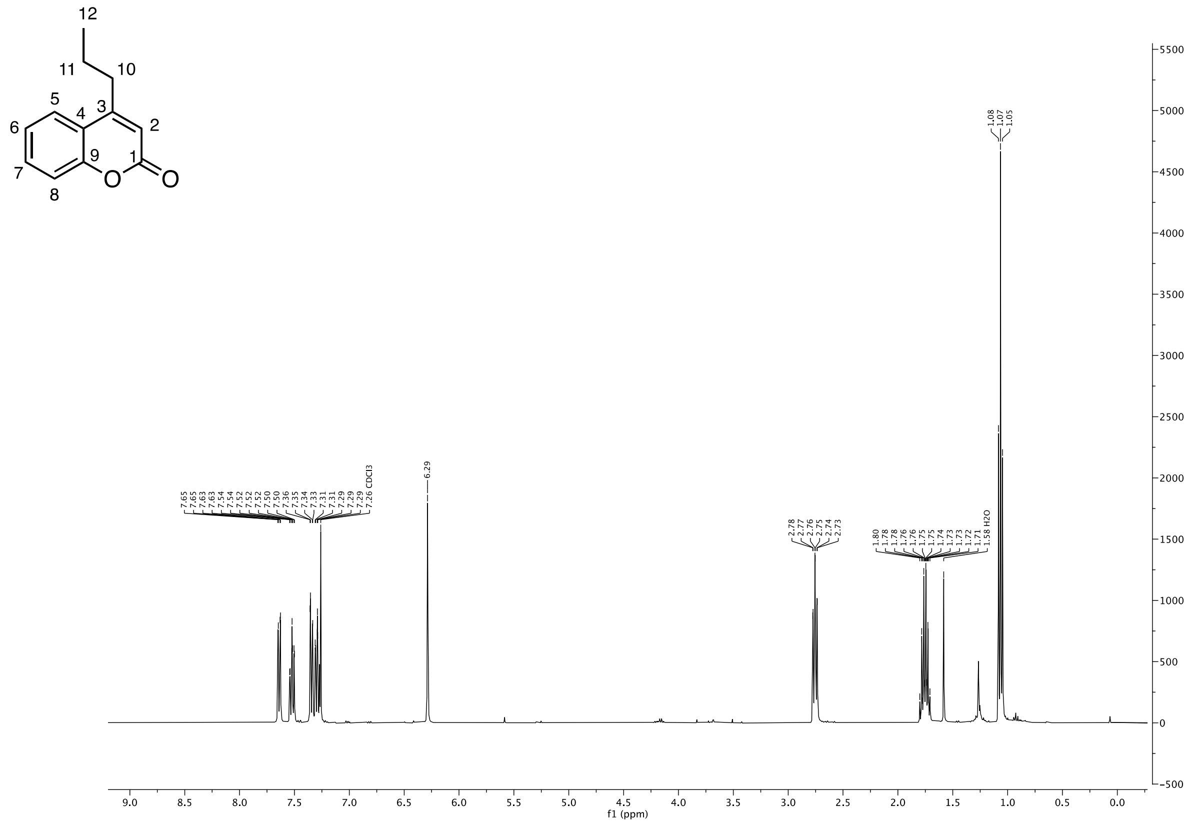

^{13}C-NMR of 4 (101 MHz, $CDCl_3$)

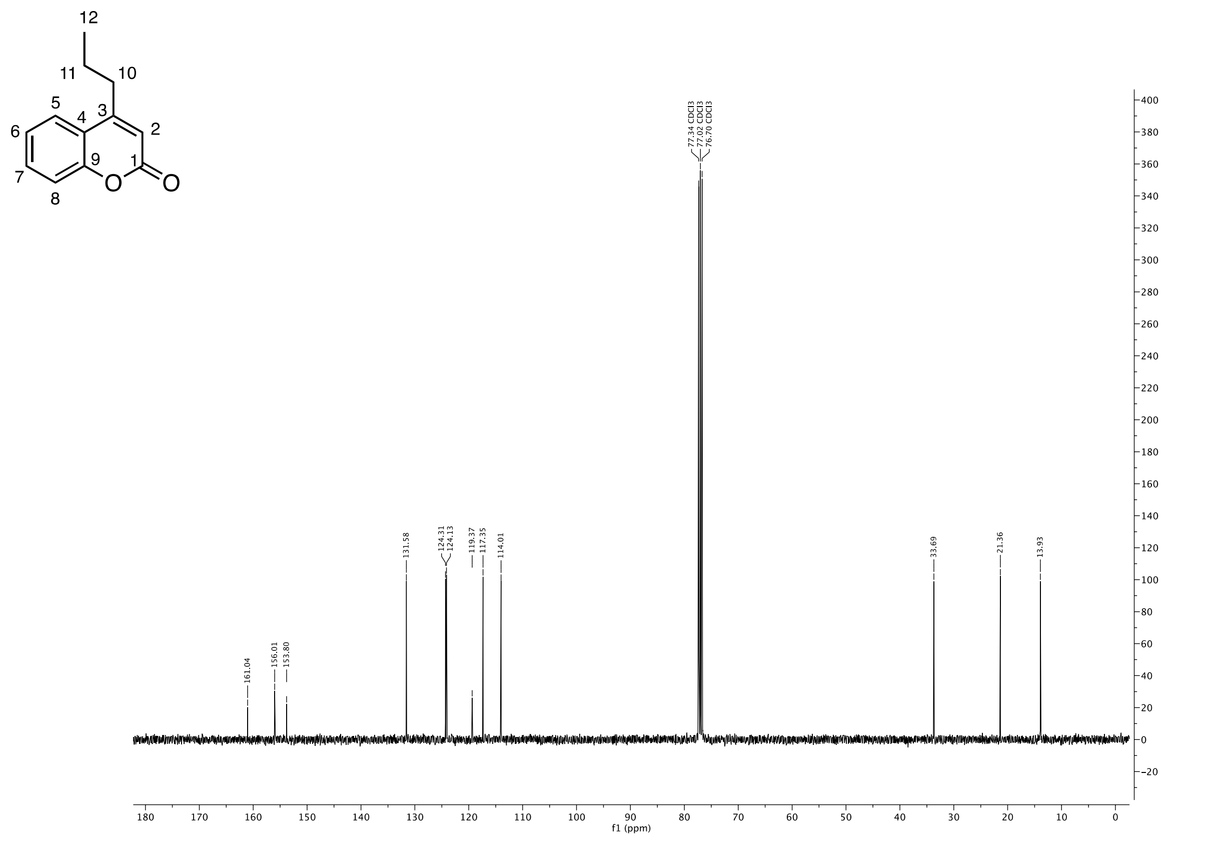

^{1}H-NMR of 5 (400 MHz, $CDCl_3$)

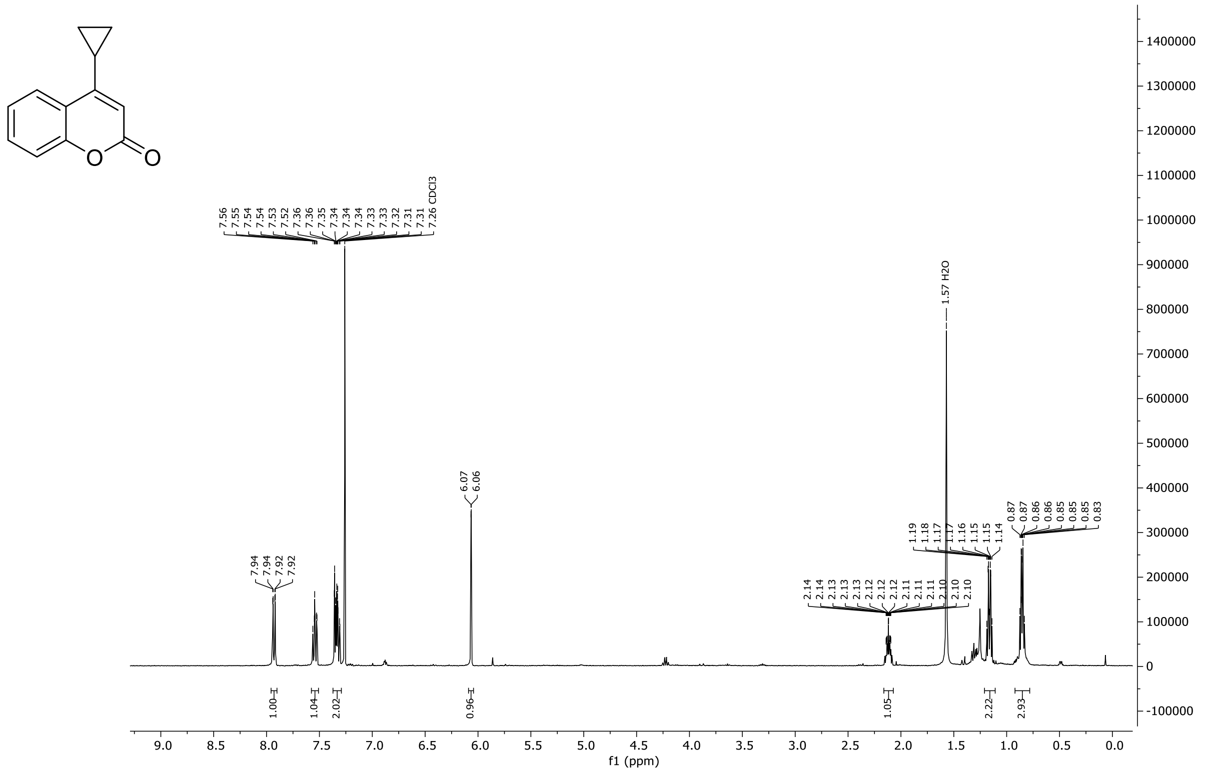

^{1}H-NMR of 6 (500 MHz, $CDCl_3$)

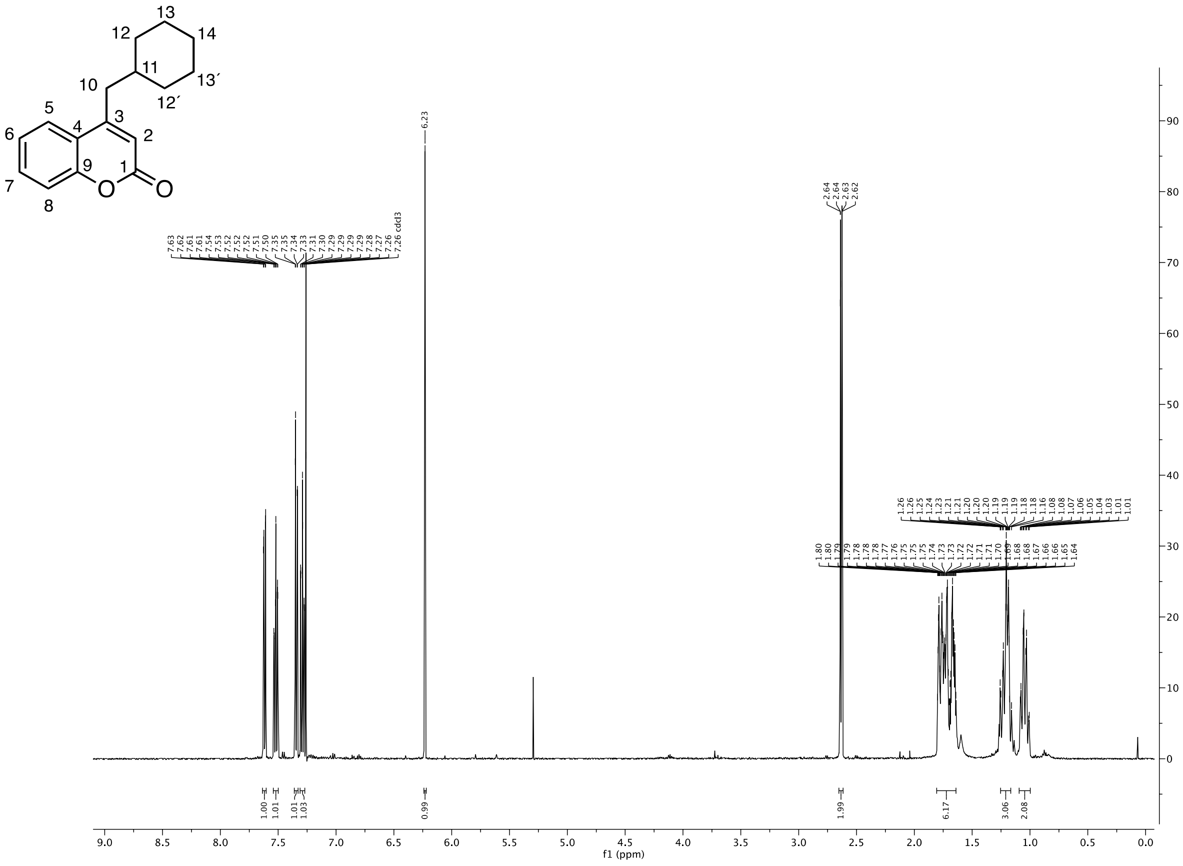

^{13}C-NMR of 6 (126 MHz, $CDCl_3$)

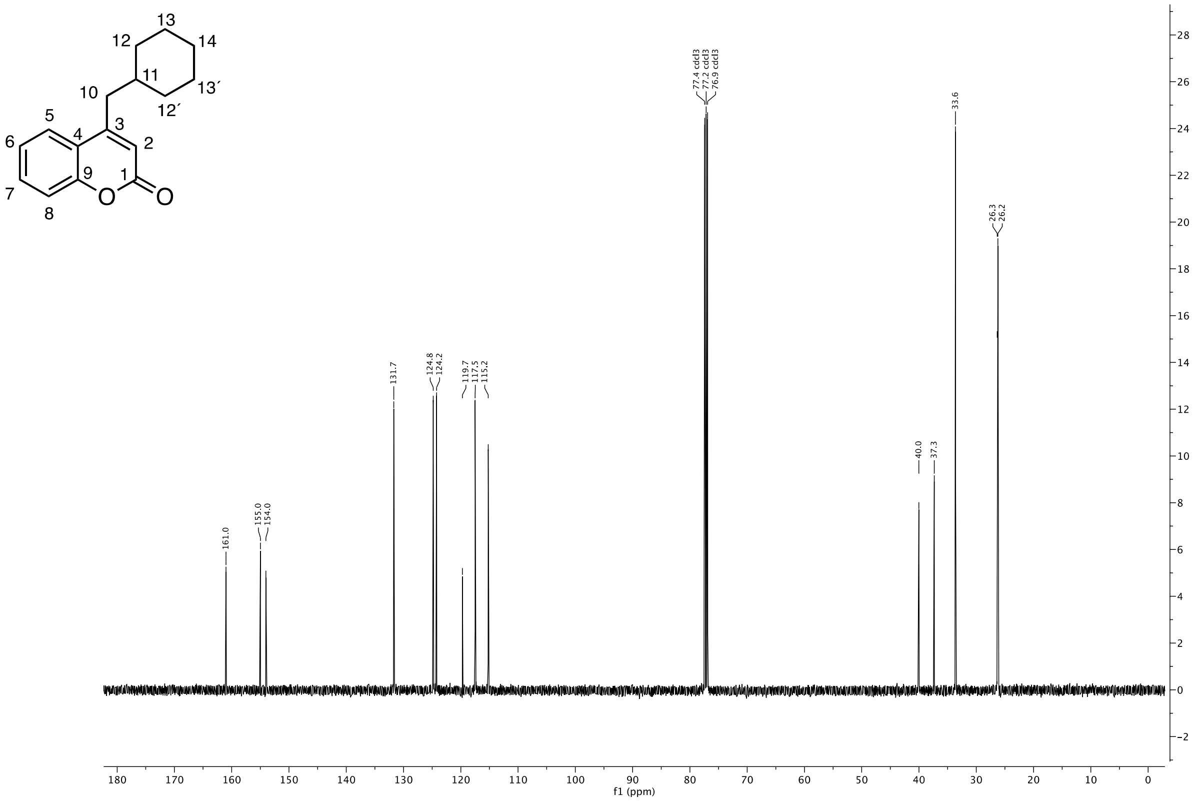

^{1}H-NMR of 7 (400 MHz, $CDCl_3$)

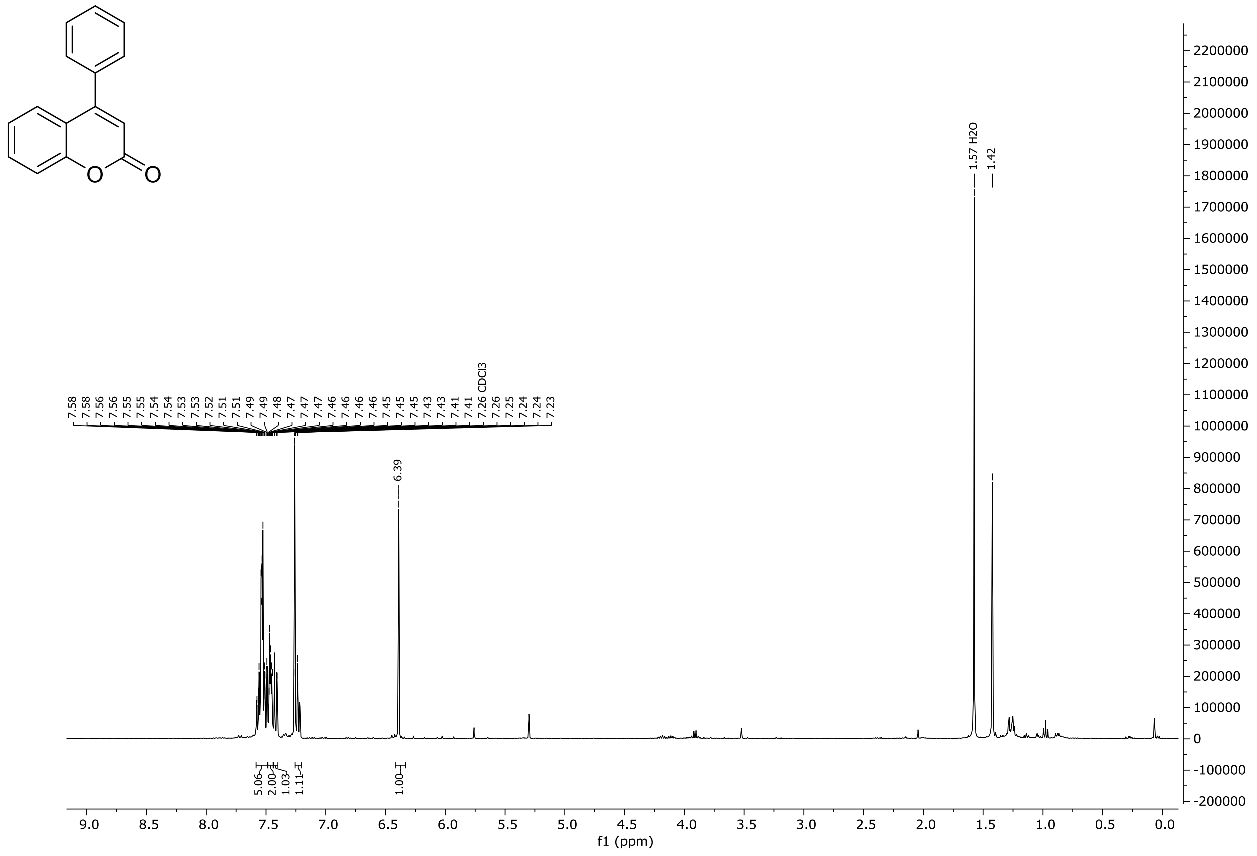

^{1}H-NMR of 8 (300 MHz, $CDCl_3$)

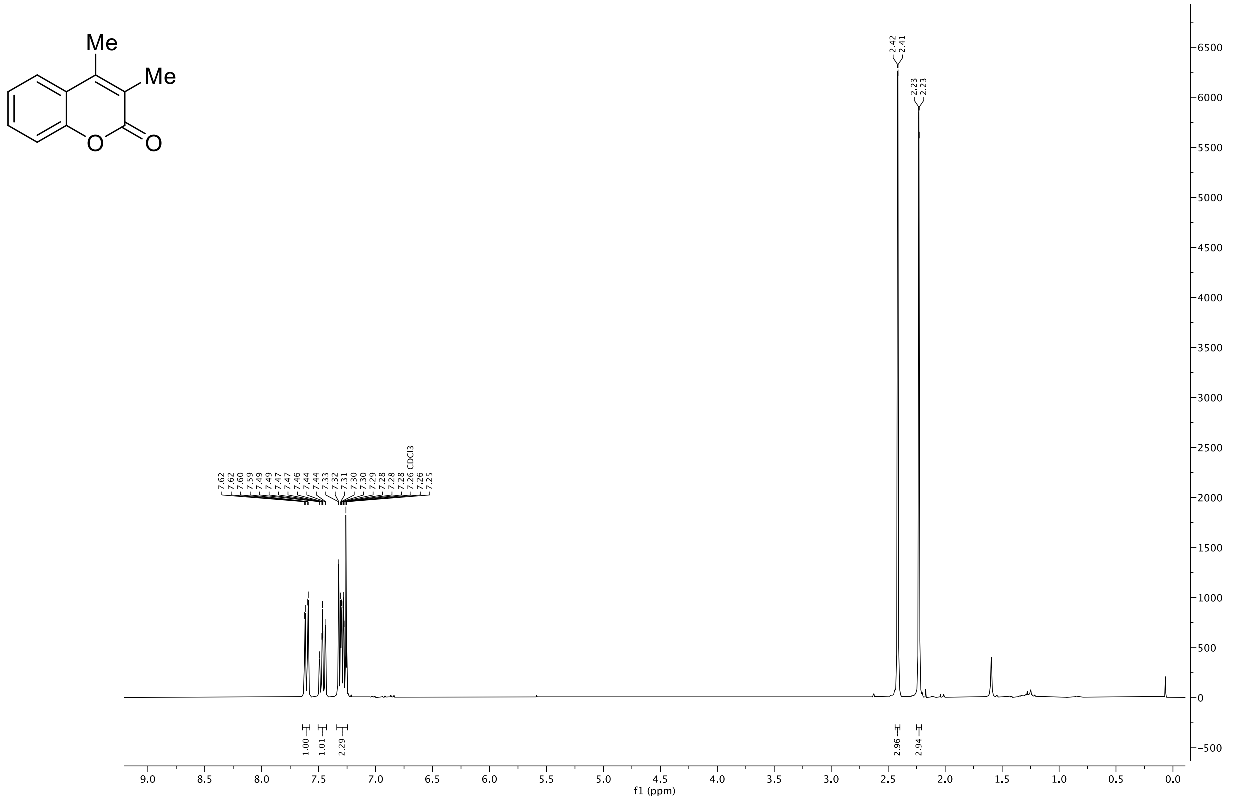

¹H-NMR of 9 (400 MHz, $CDCl_3$)

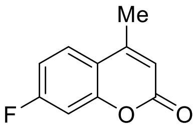

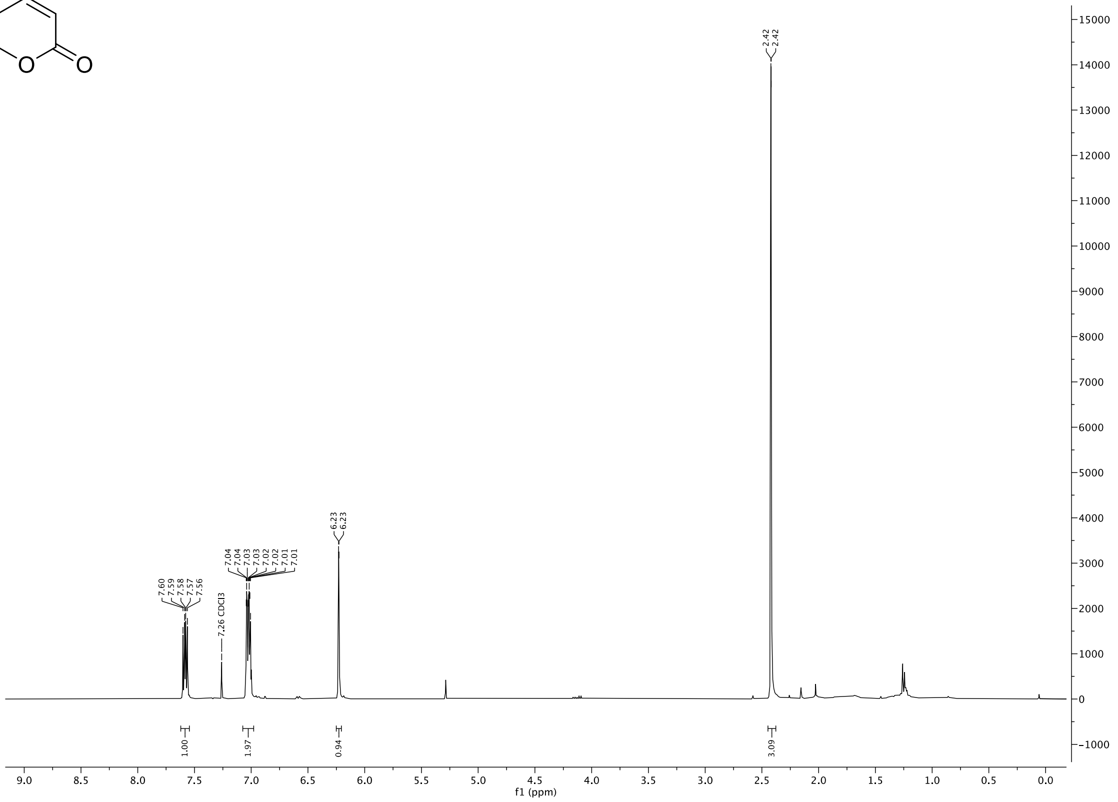

¹⁹F-NMR of 9 (376 MHz, $CDCl_3$)

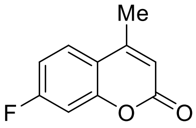

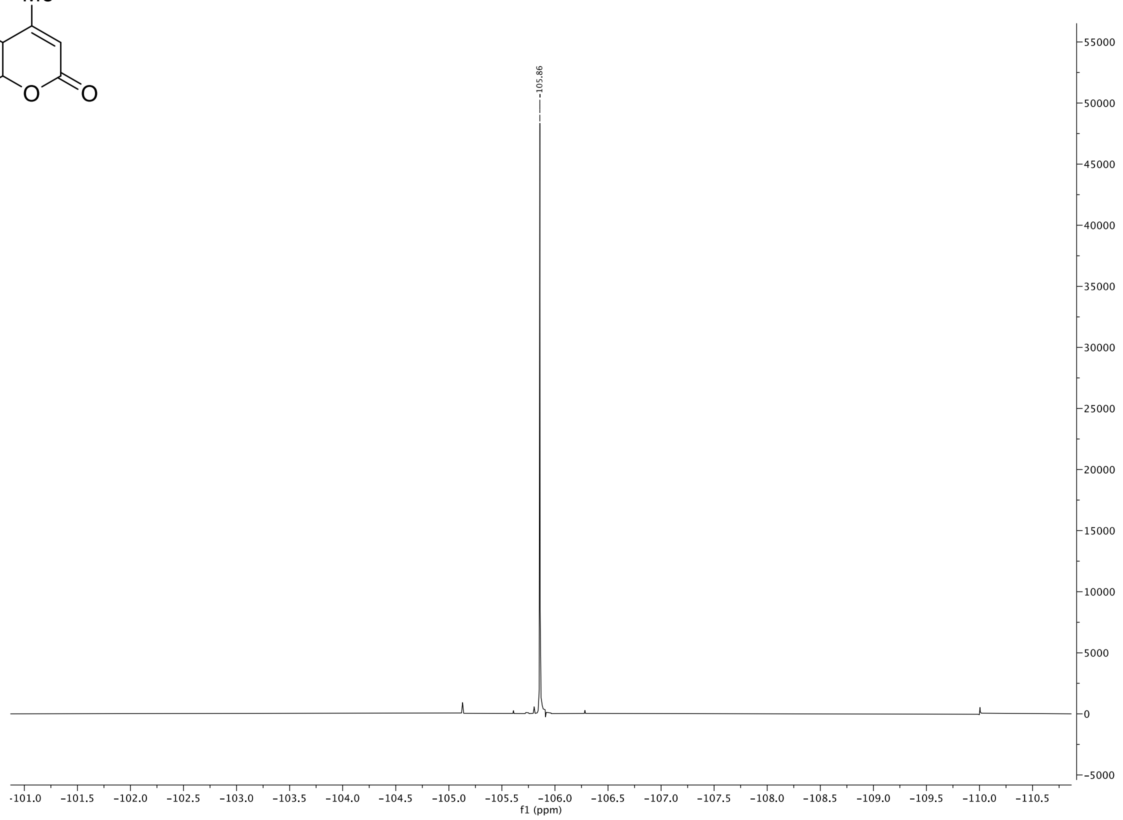

SUPPORTING INFORMATION

^{1}H-NMR of 10 (300 MHz, DMSO-d_6)

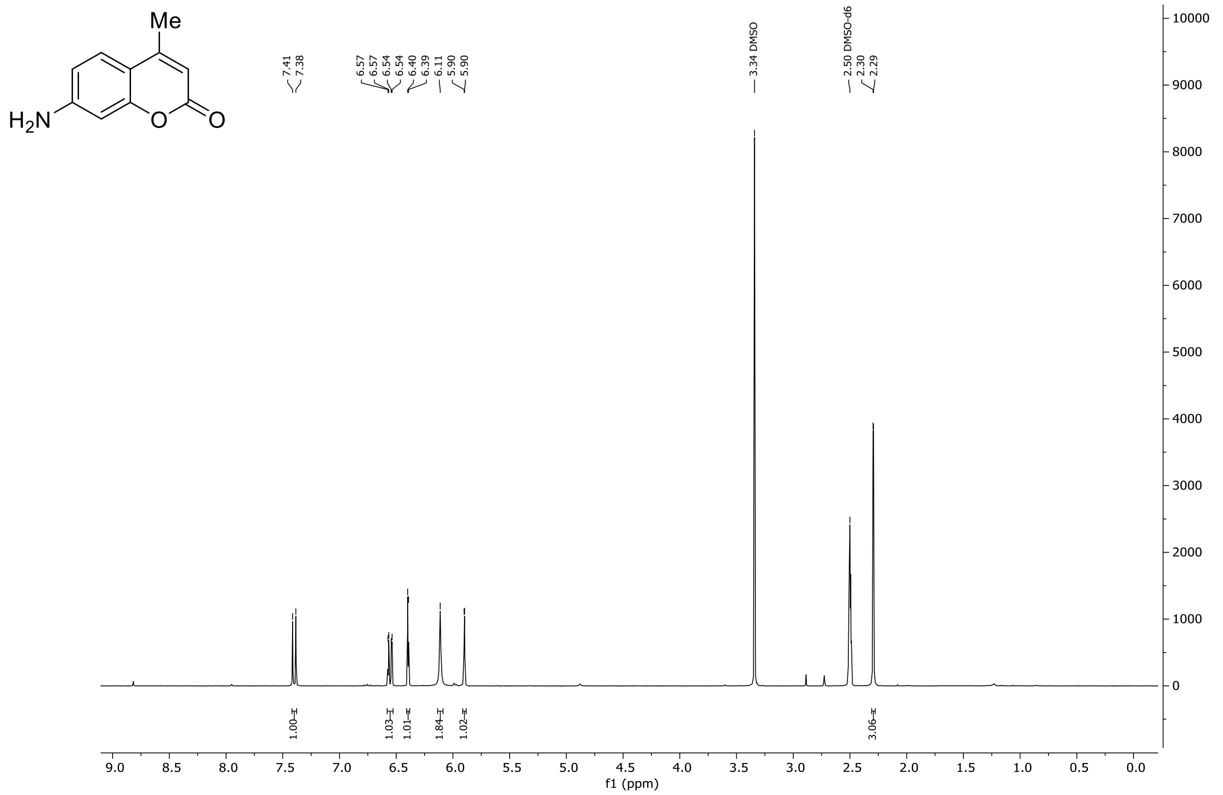

^{1}H-NMR of 11 (599 MHz, $CDCl_3$)

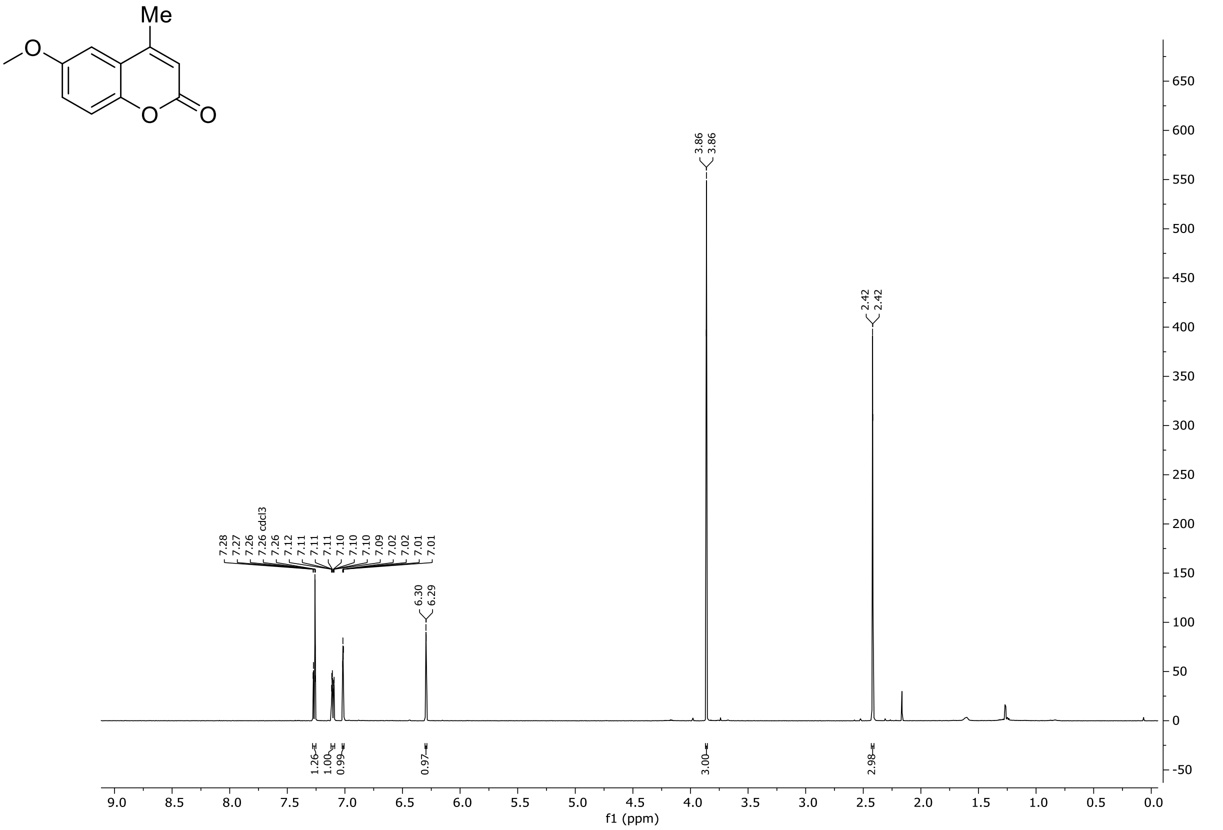

^{1}H-NMR of 12 (300 MHz, $CDCl_3$)

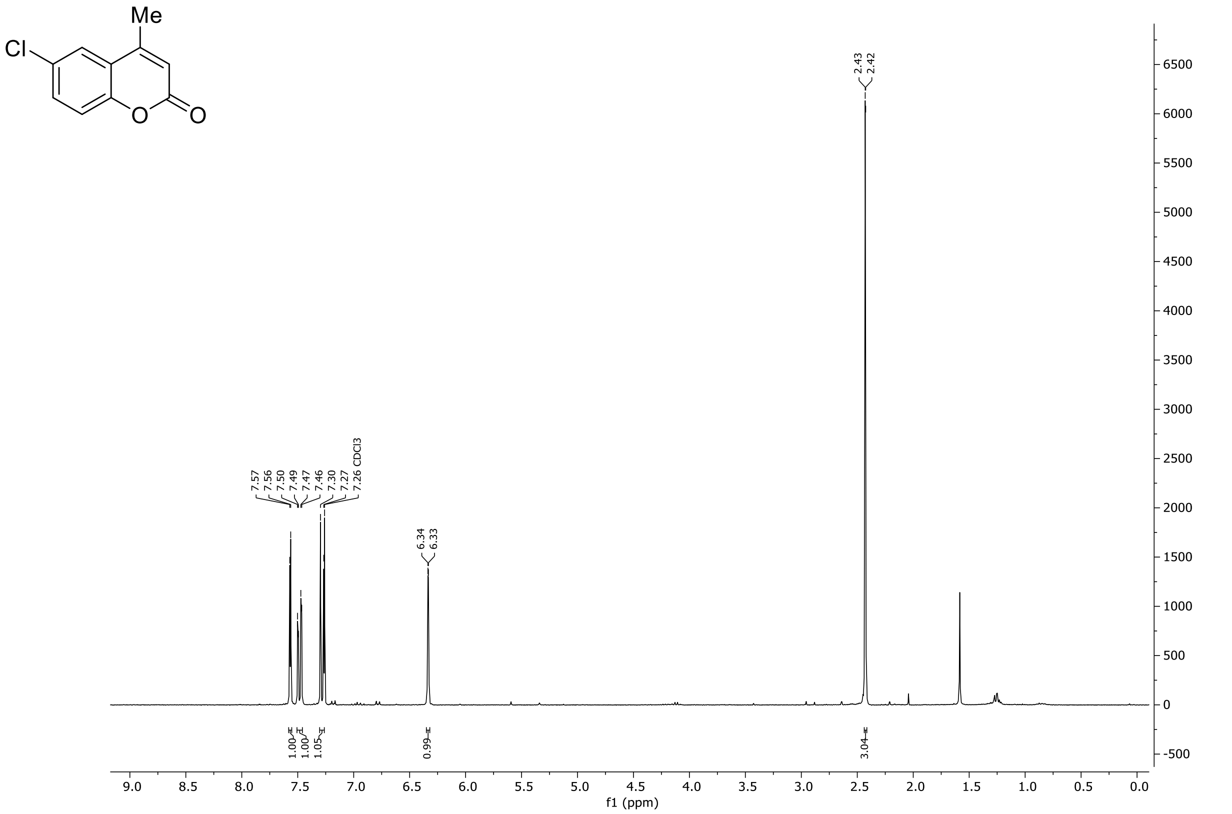

^{1}H-NMR of 13 (300 MHz, $CDCl_3$)

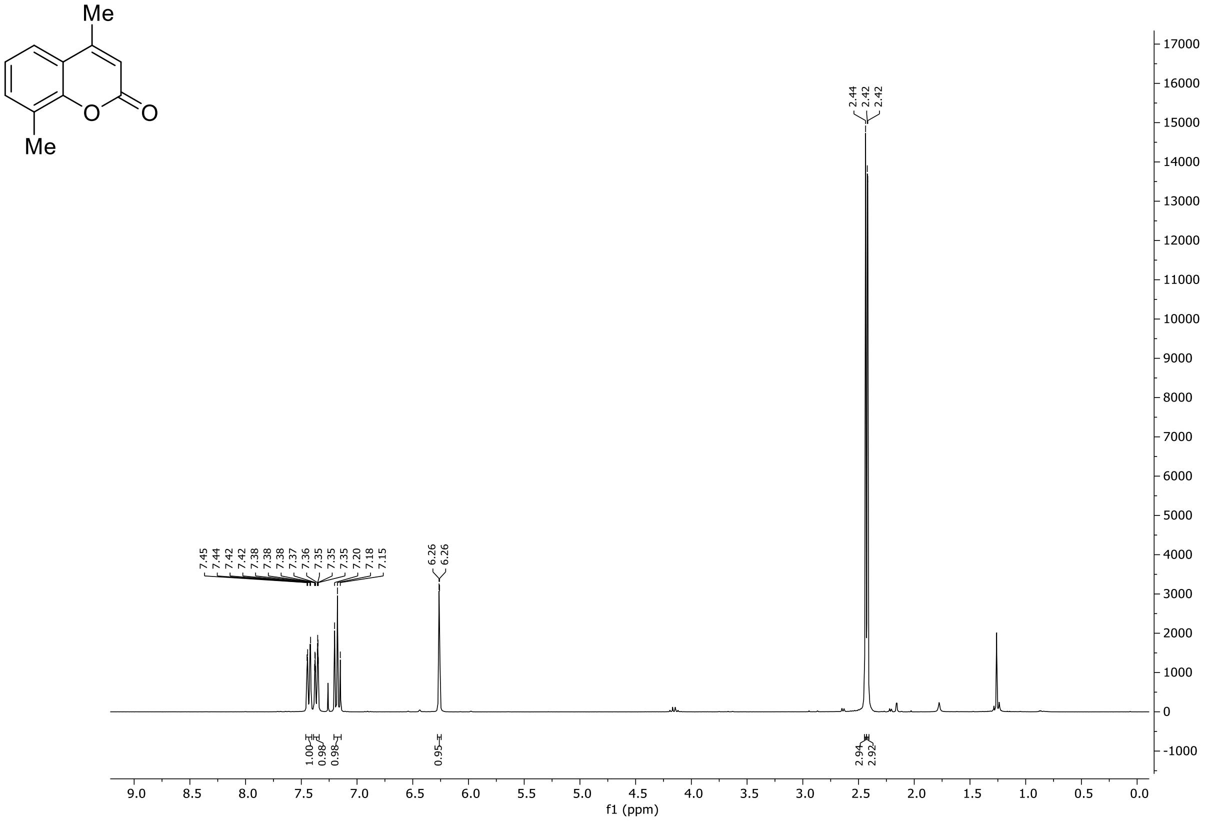

^{13}C-NMR of 13 (300 MHz, CDCl$_3$)

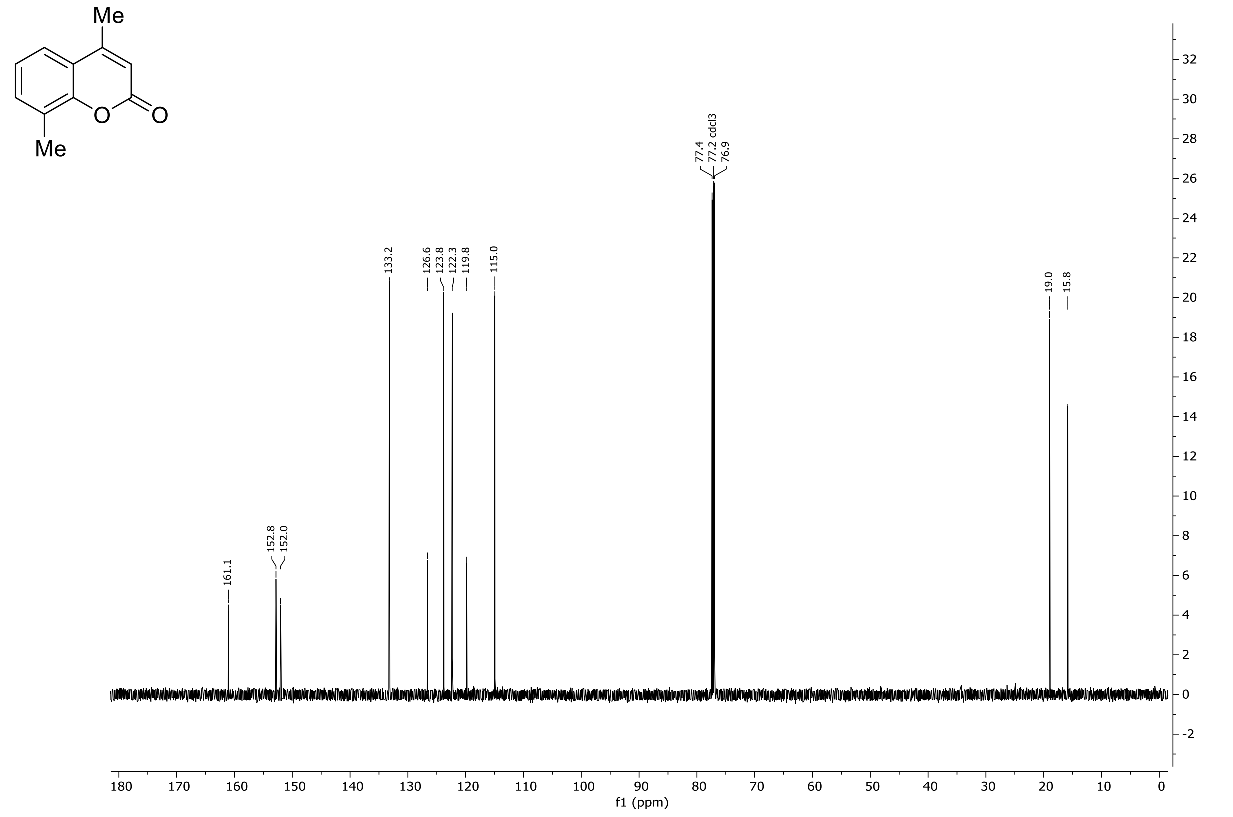

^{1}H-NMR of 14 (300 MHz, CDCl$_3$)

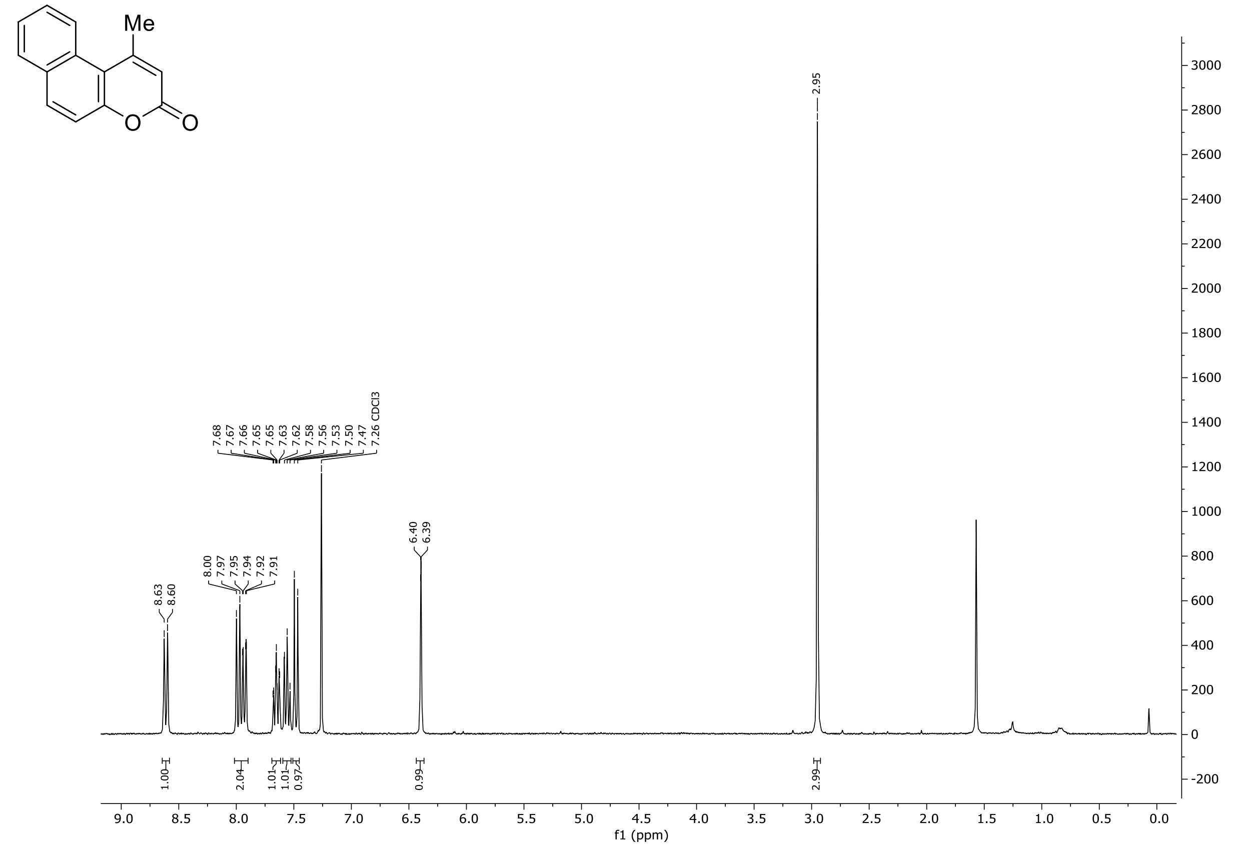

SUPPORTING INFORMATION

^{1}H-NMR of 15 (500 MHz, $CDCl_3$)

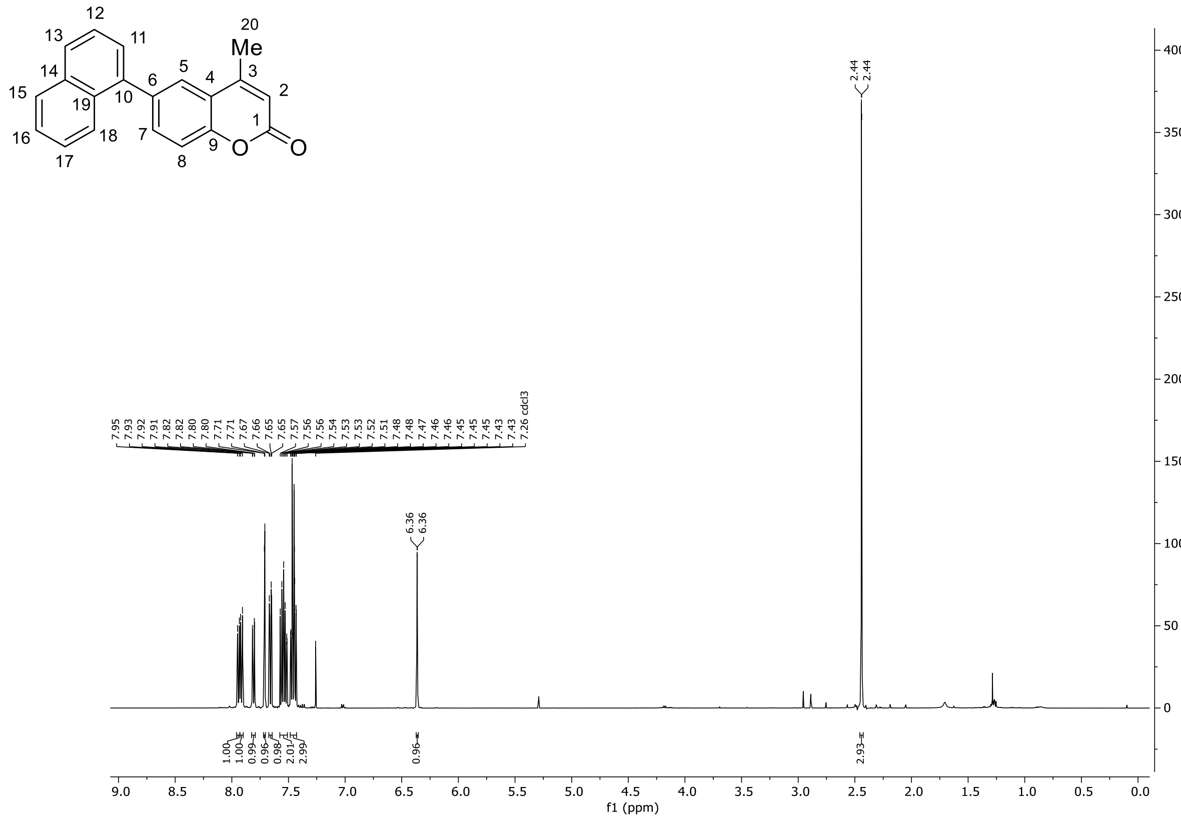

^{13}C-NMR of 15 (126 MHz, $CDCl_3$)

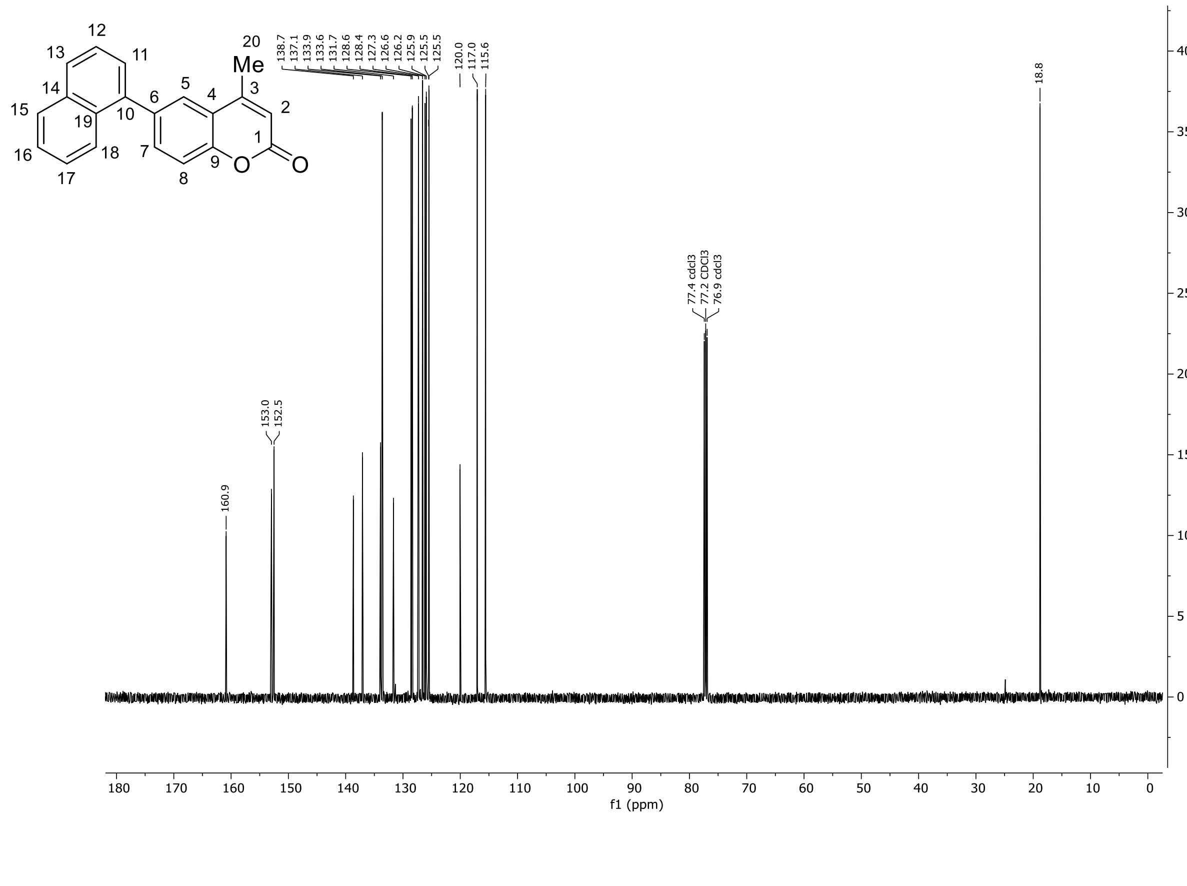

^{1}H-NMR of 16 (500 MHz, $CDCl_3$)

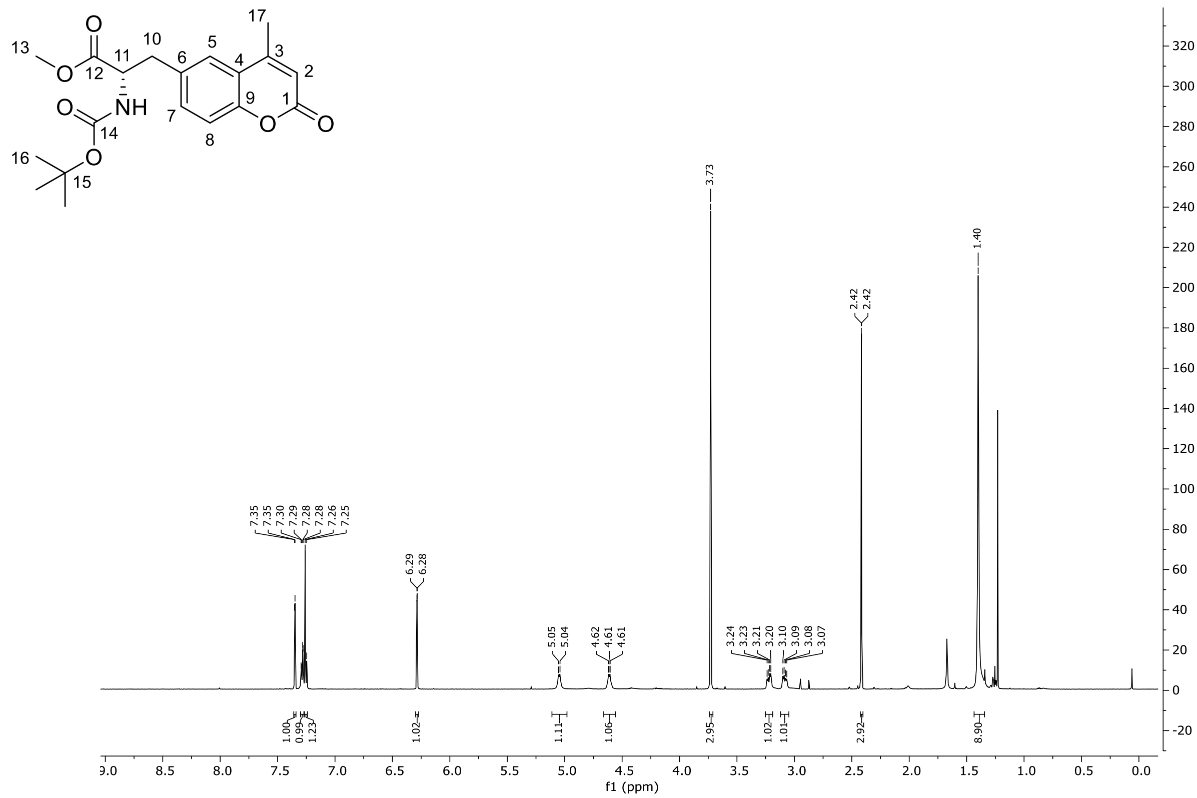

^{13}C-NMR of 16 (126 MHz, $CDCl_3$)

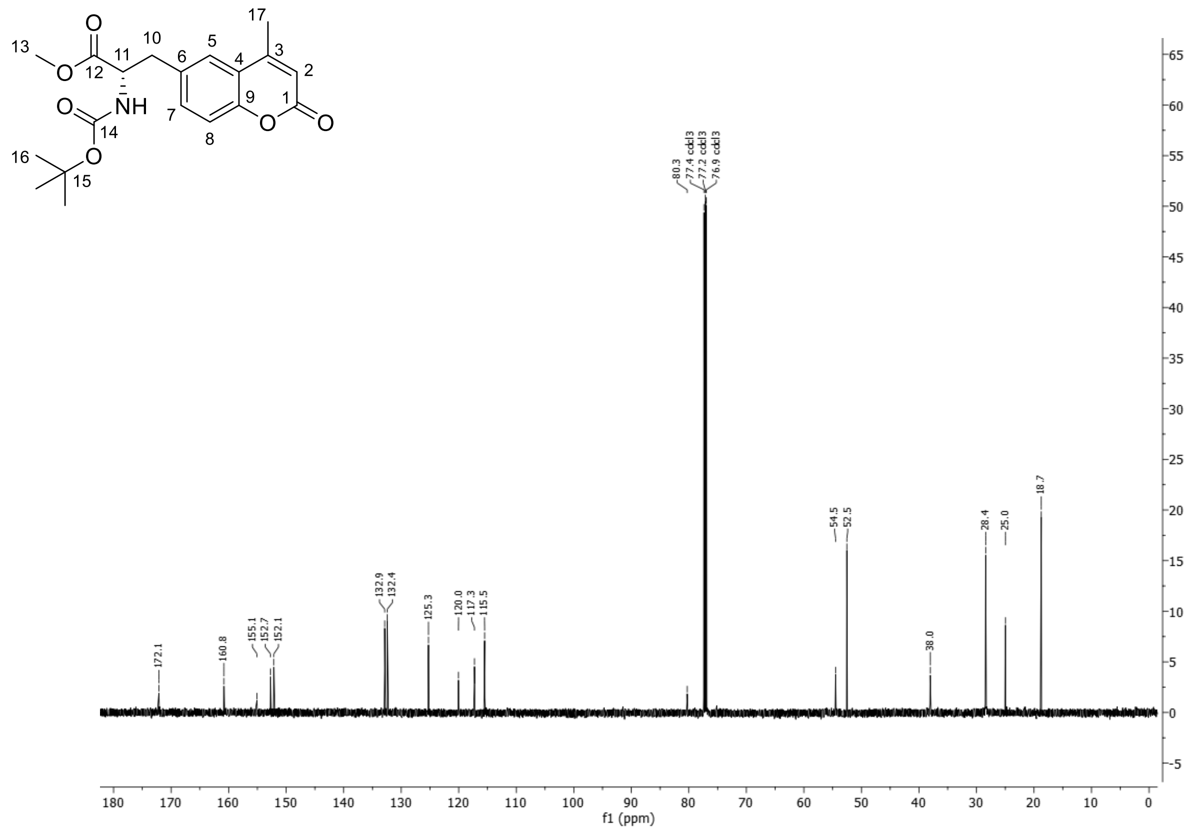

^{1}H-NMR of 17 (599 MHz, $CDCl_3$)

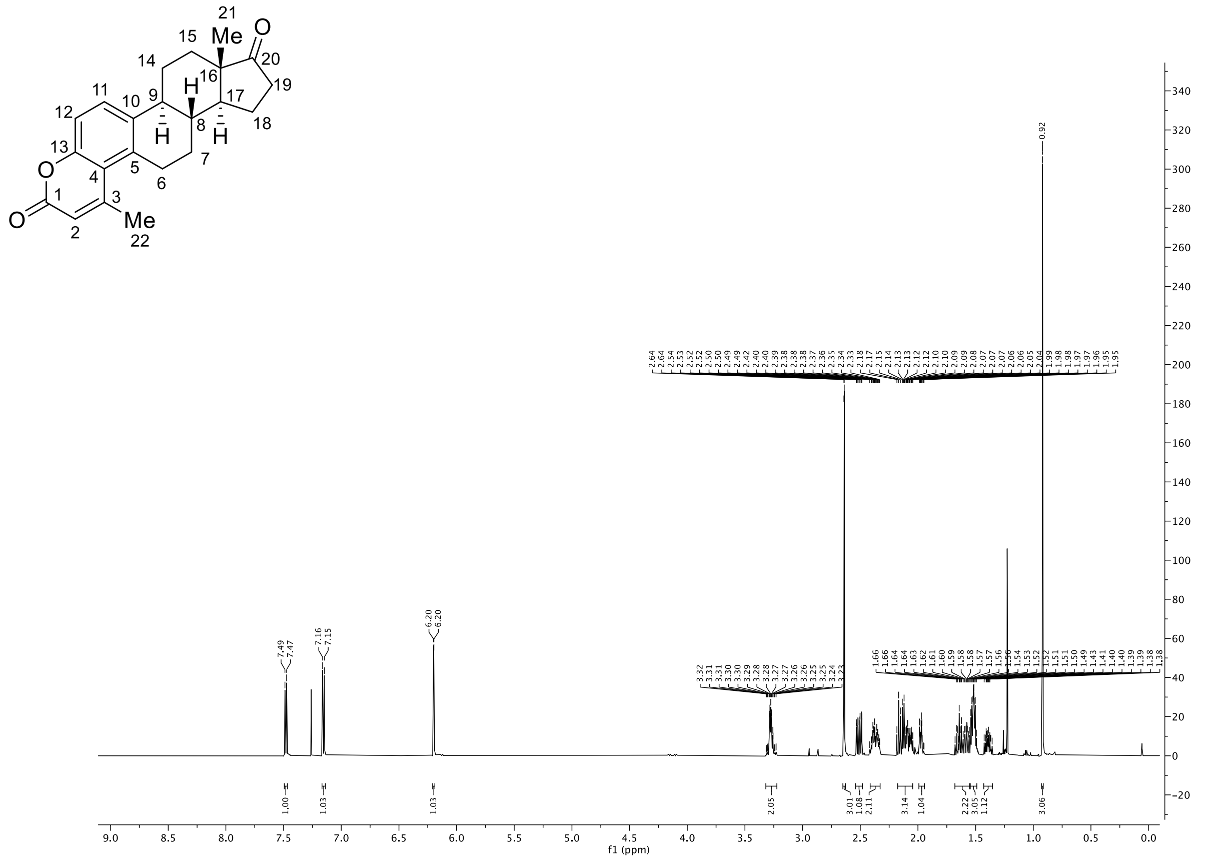

^{13}C-NMR of 17 (151 MHz, $CDCl_3$)

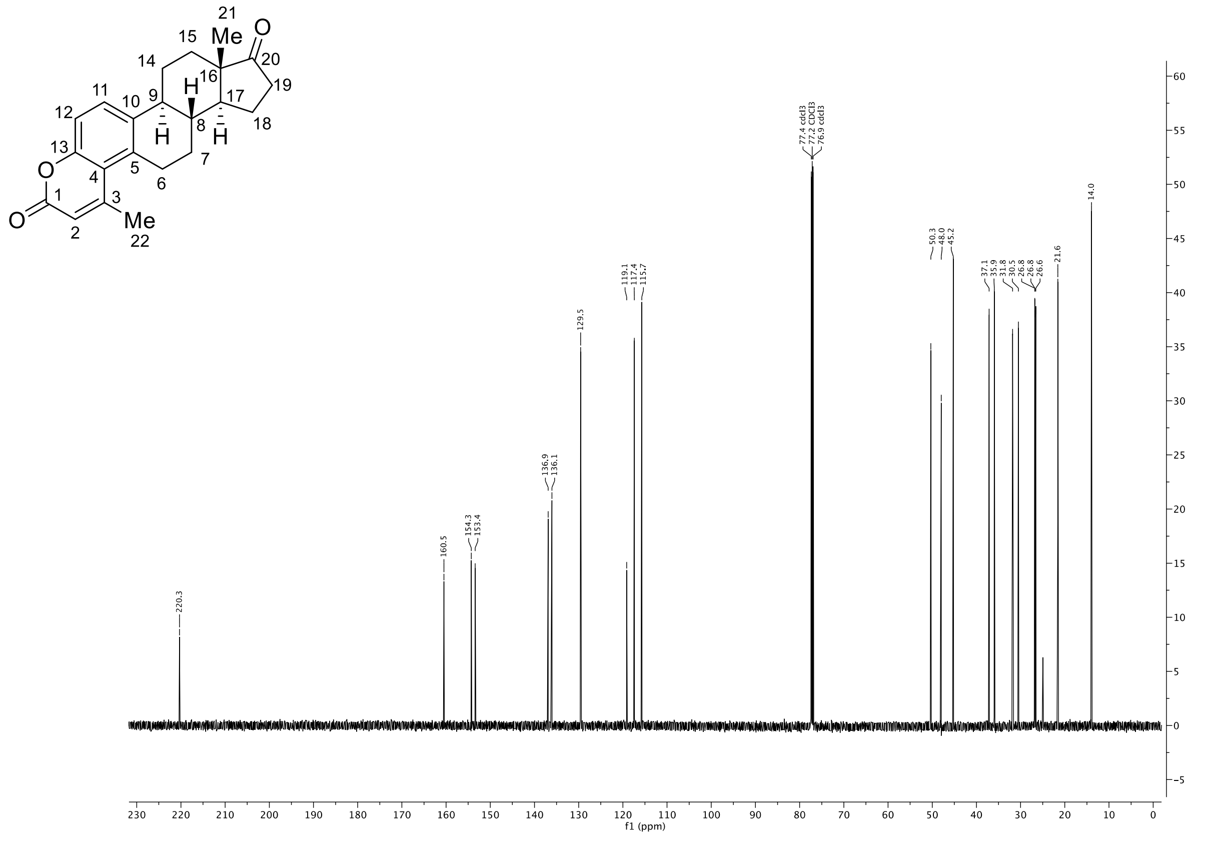

^{1}H-NMR of 18 (599 MHz, $CDCl_3$)

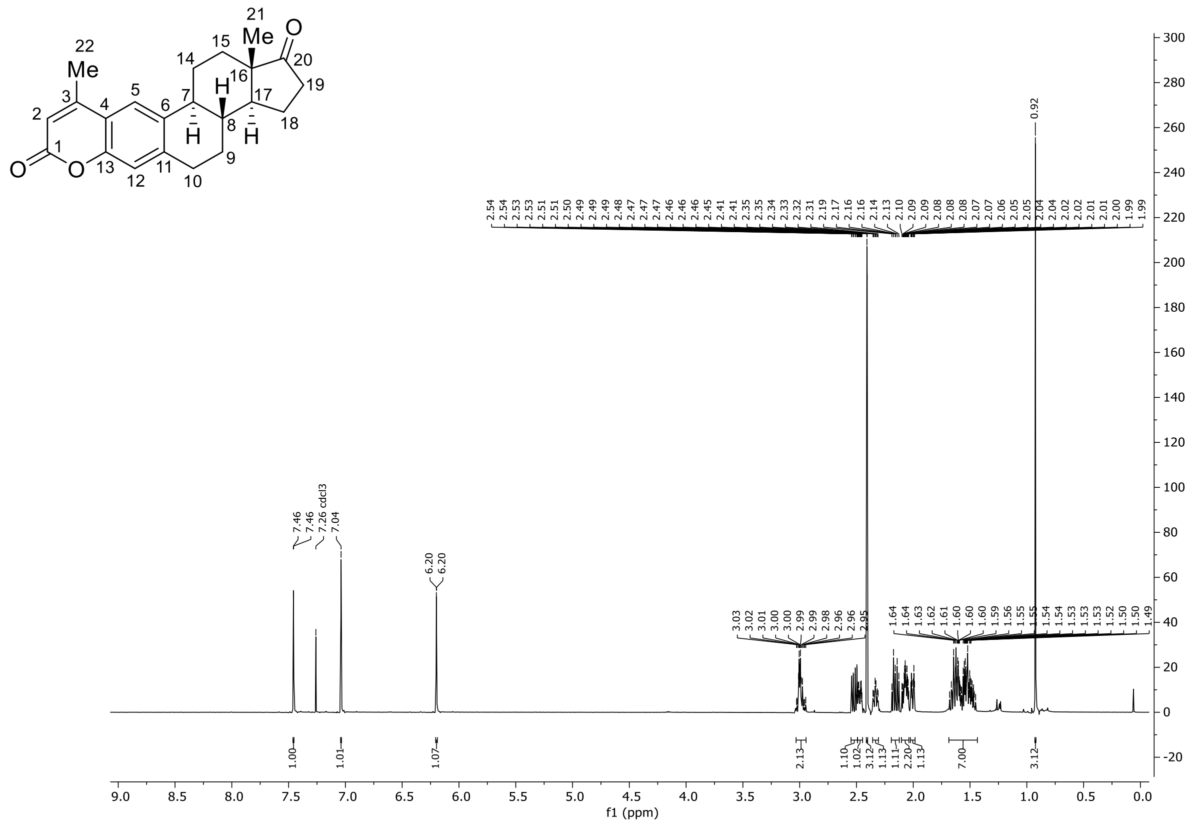

^{13}C-NMR of 18 (151 MHz, $CDCl_3$)

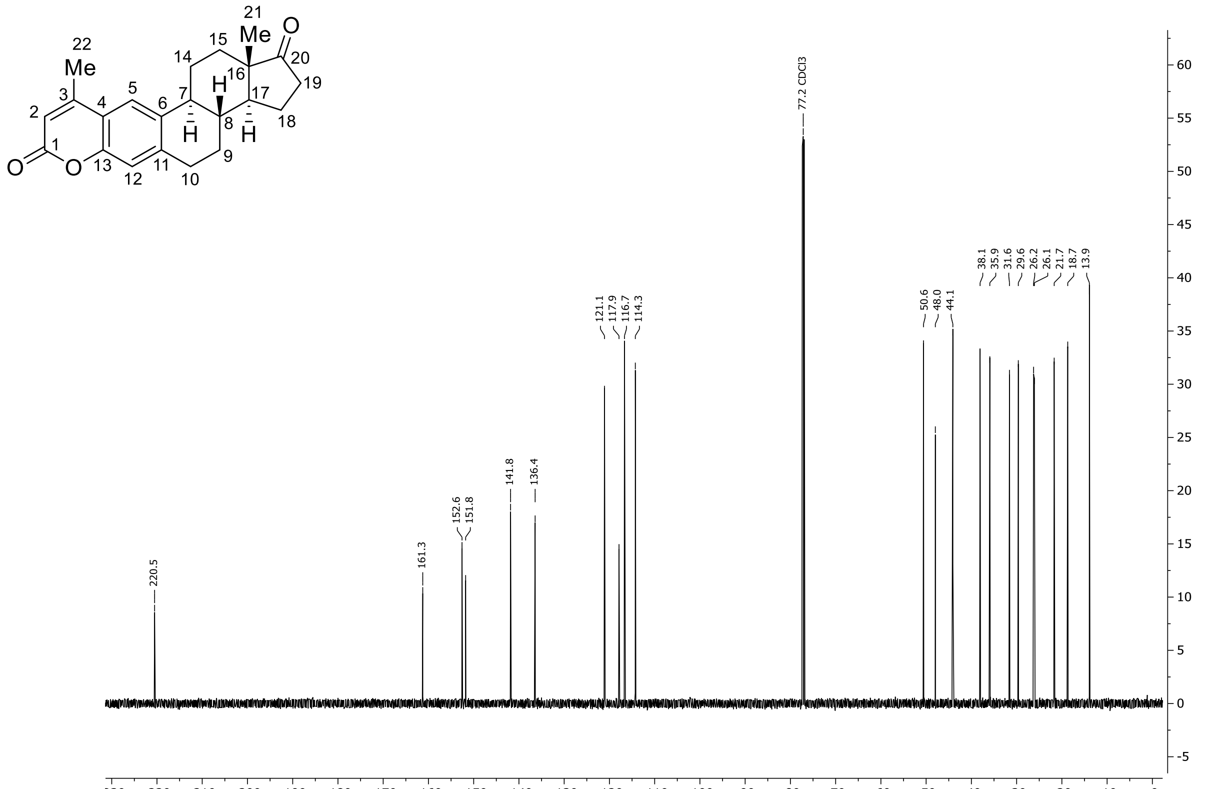

^{1}H-NMR of 19 (599 MHz, $CDCl_3$)

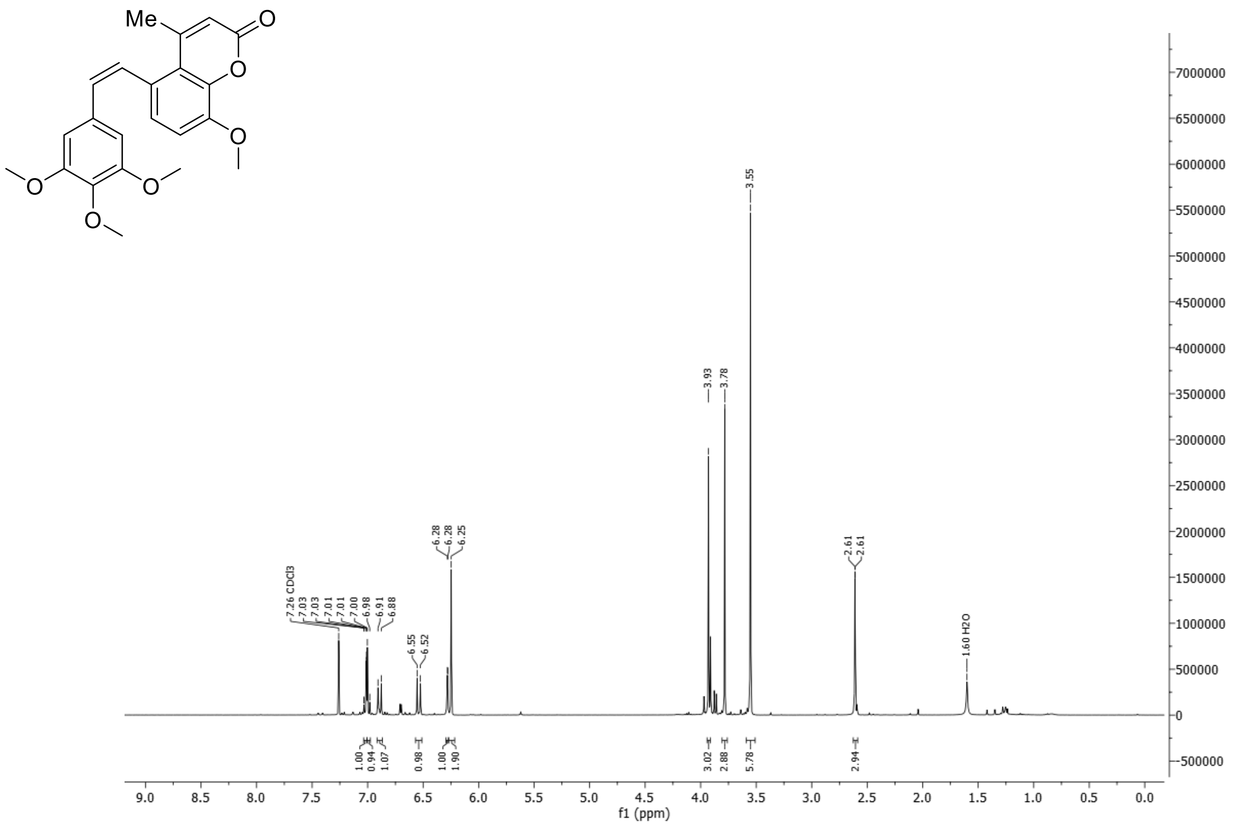

^{13}C-NMR of 19 (126 MHz, $CDCl_3$)

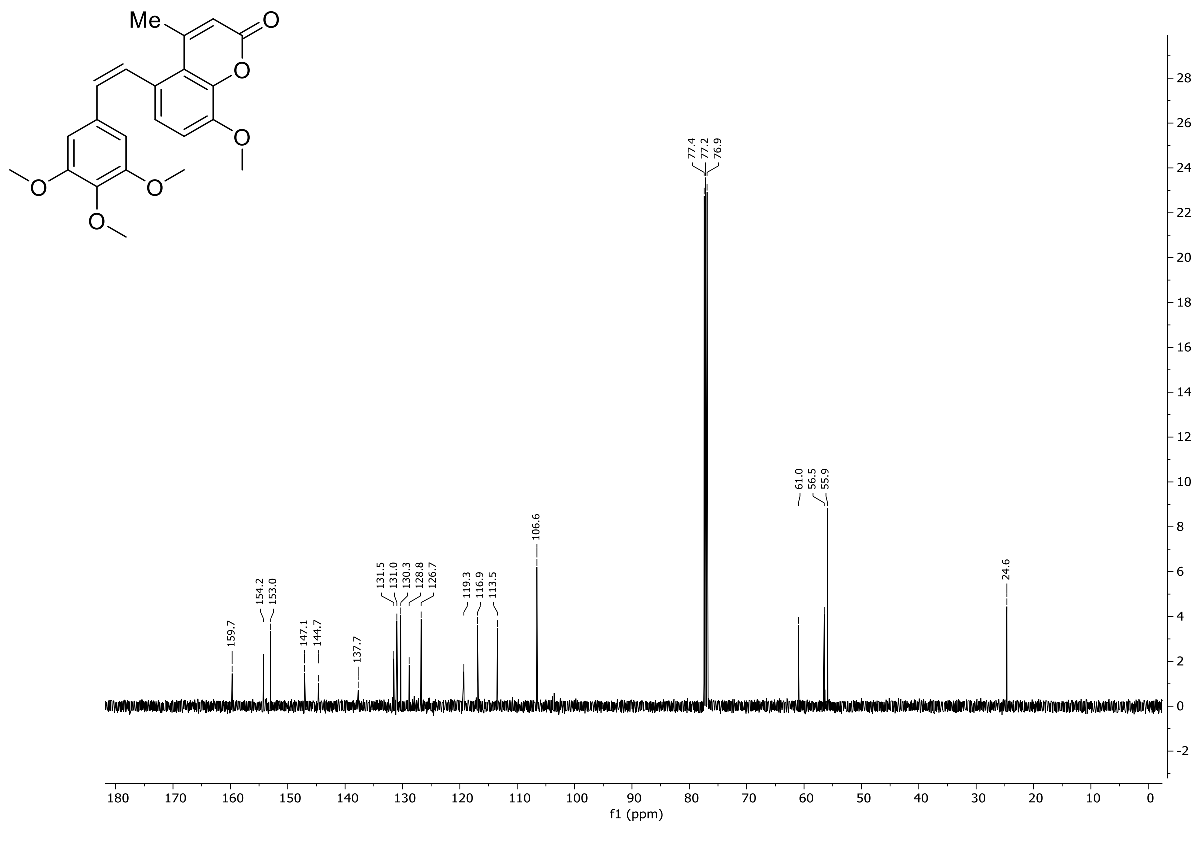

^{1}H-NMR of 22 (400 MHz, $CDCl_3$)

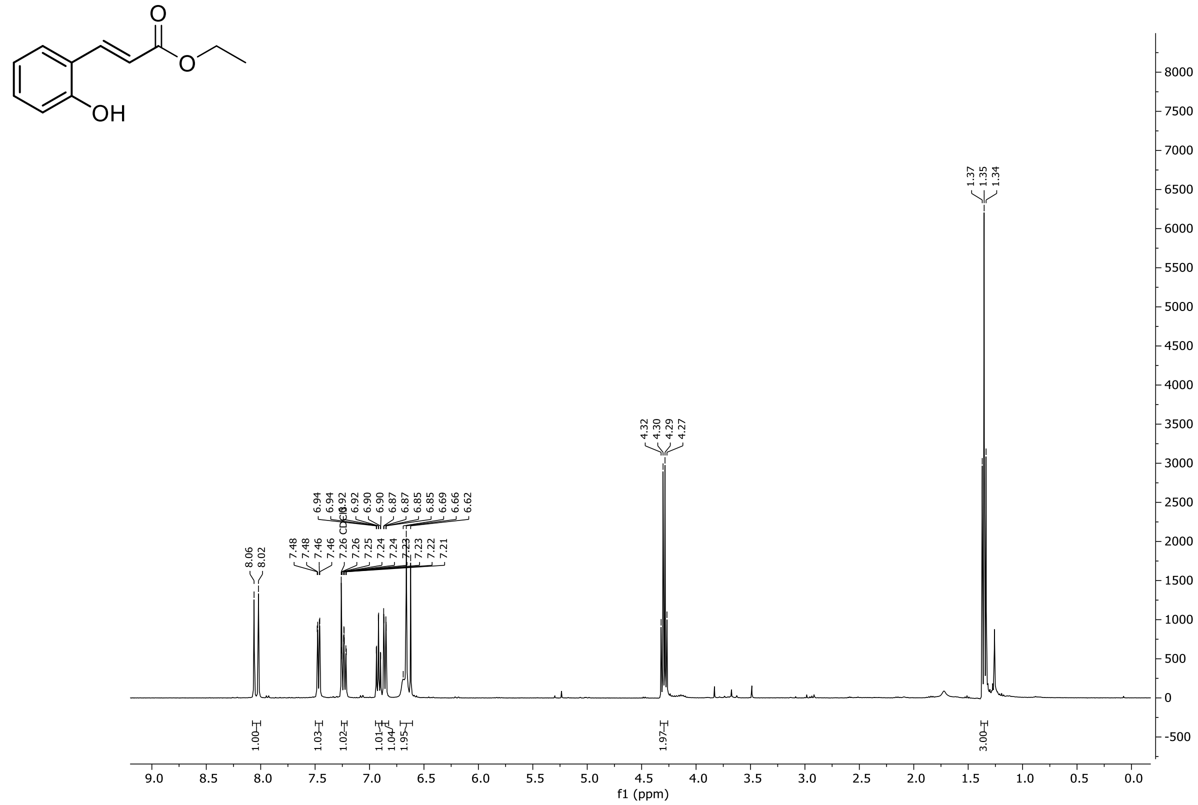

^{1}H-NMR of 23 (400 MHz, $CDCl_3$)

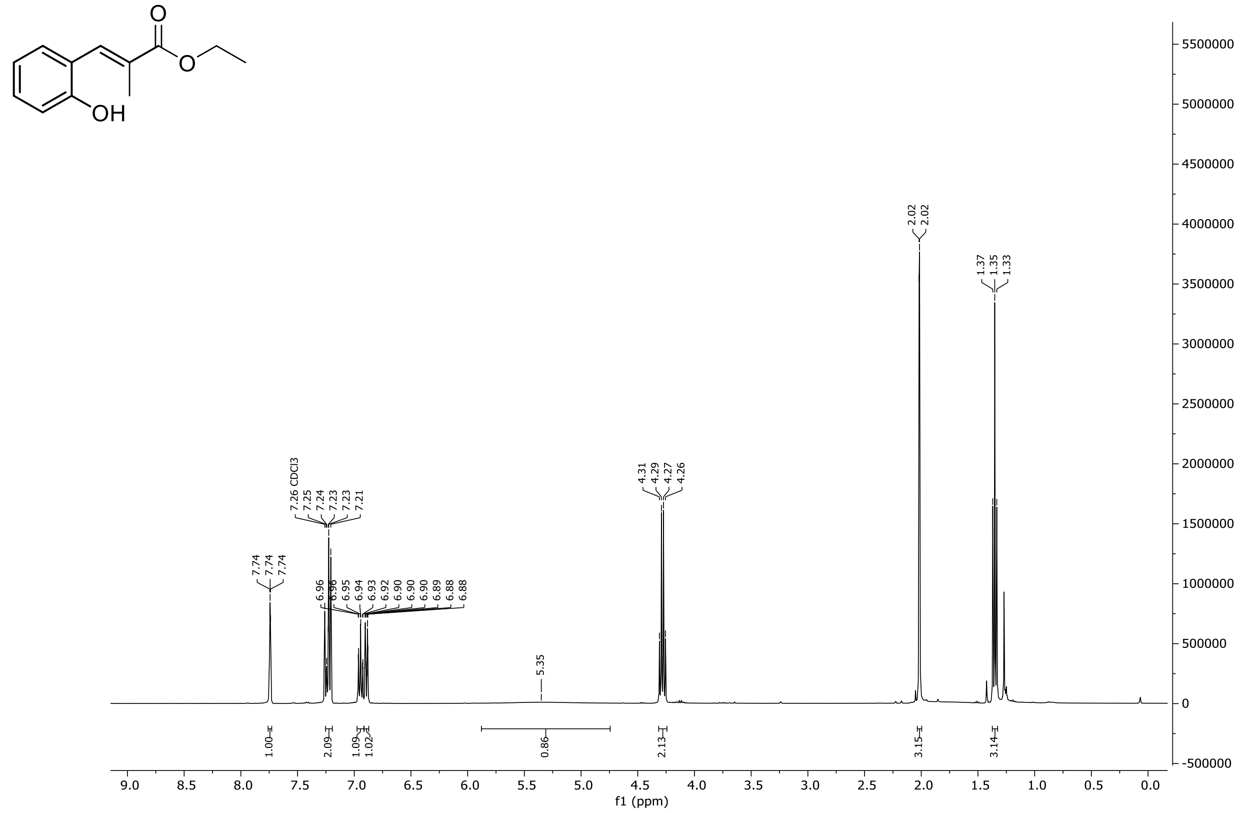

^{13}C-NMR of 23 (151 MHz, $CDCl_3$)

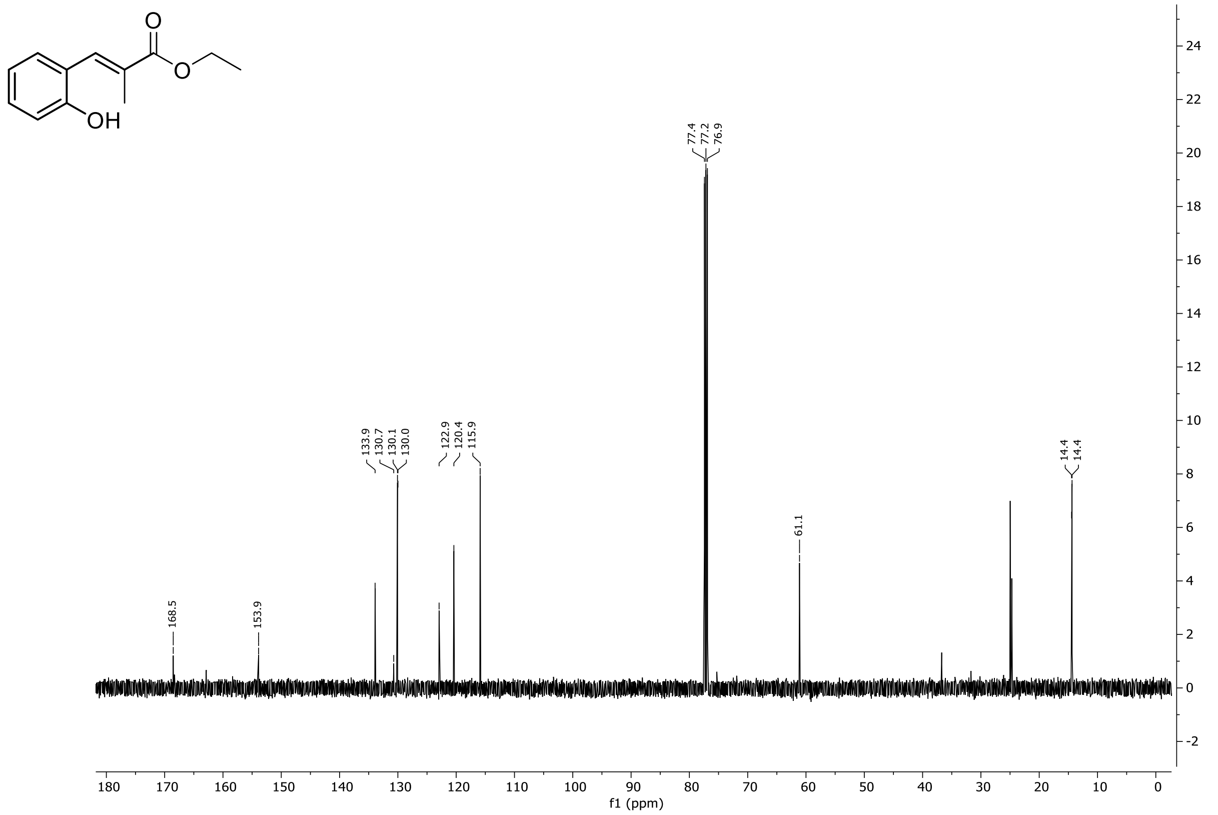

^{1}H-NMR of 24 (400 MHz, $CDCl_3$)

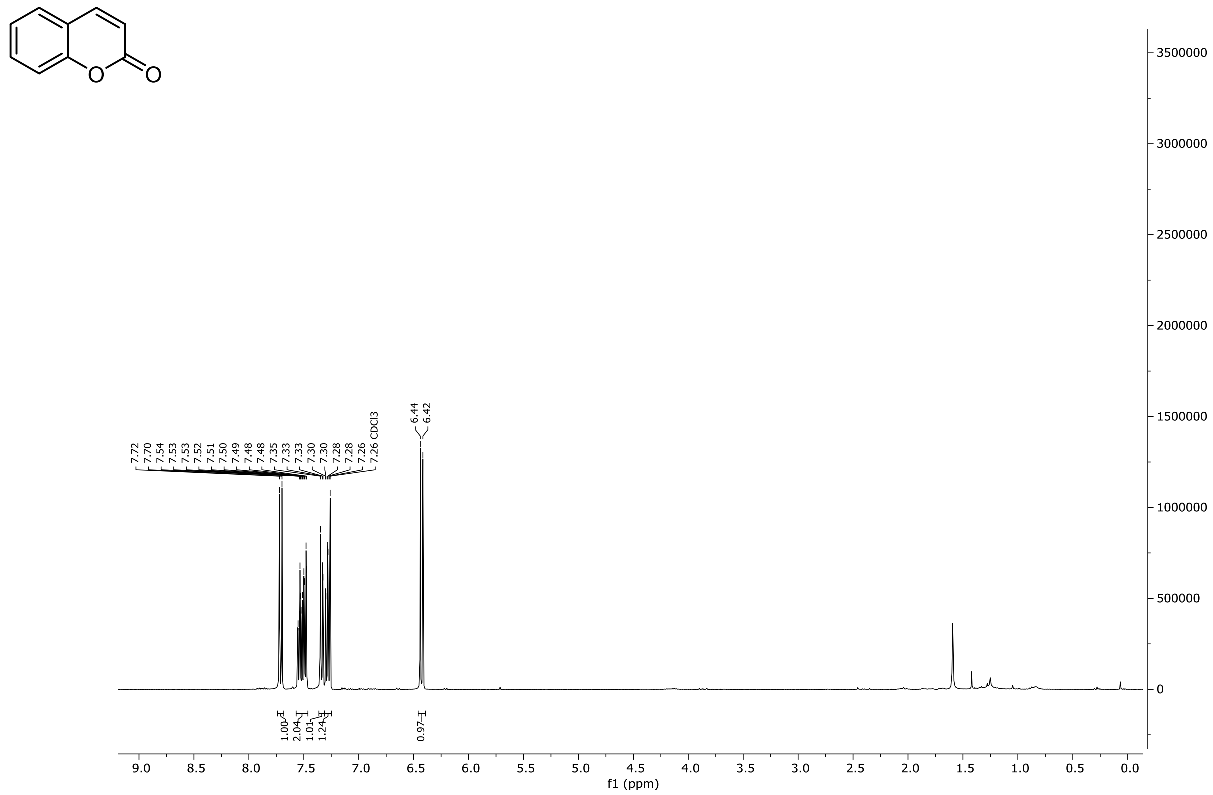

¹H-NMR of 25 (400 MHz, $CDCl_3$)

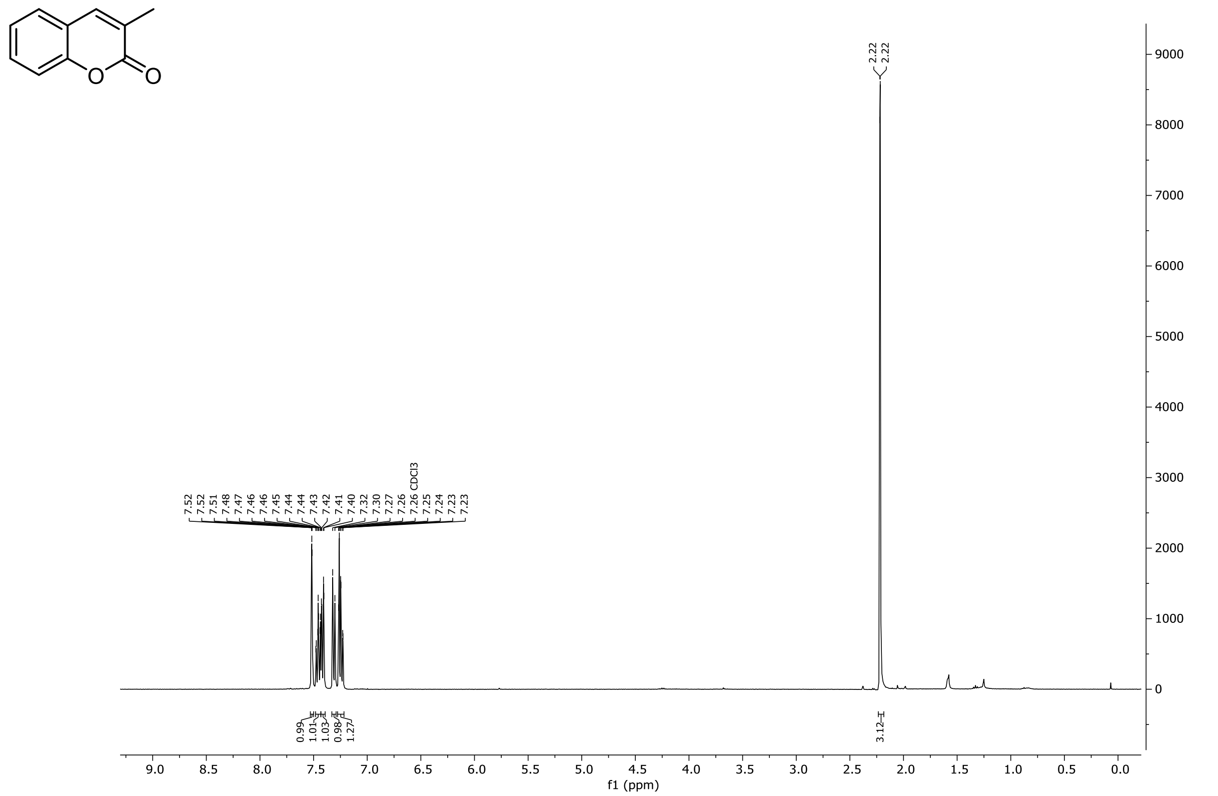

¹H-NMR of 26 (400 MHz, $CDCl_3$)

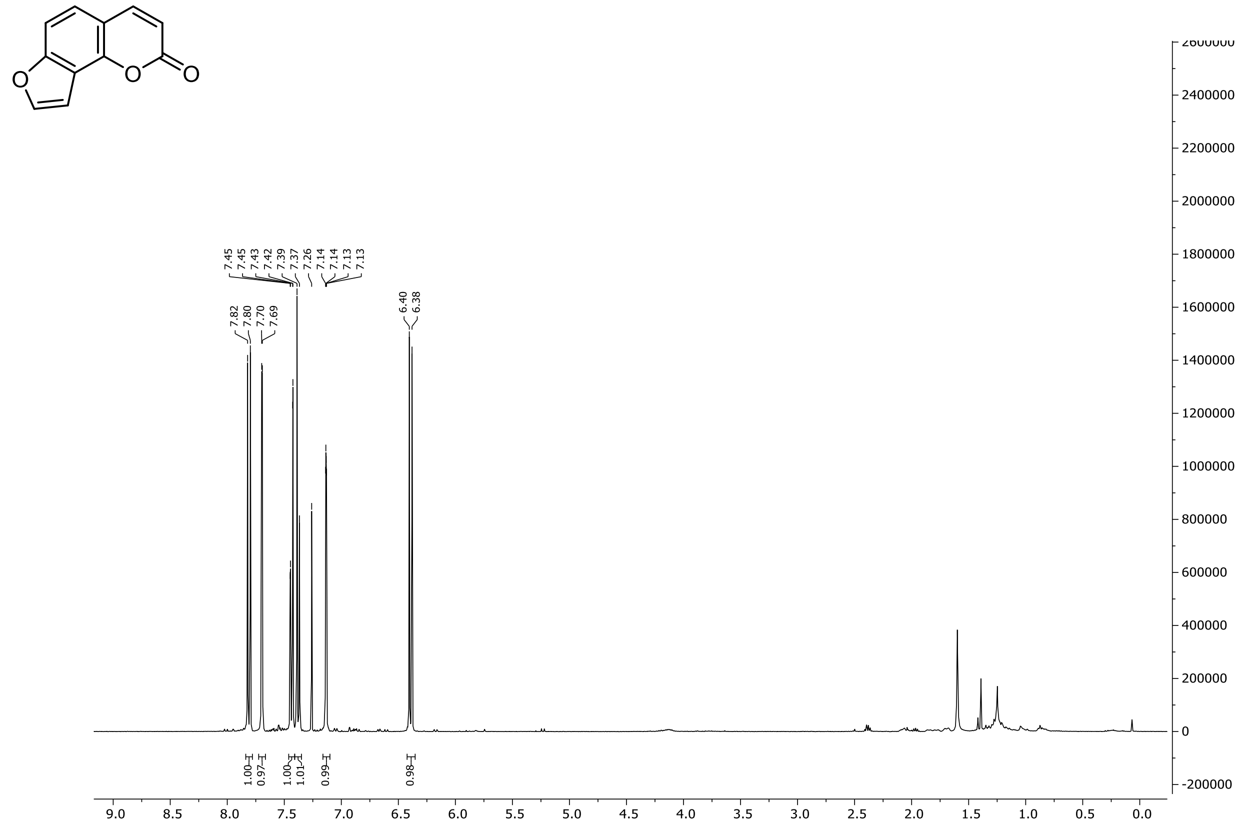

^{1}H-NMR of 27 (400 MHz, Methanol-d_4)

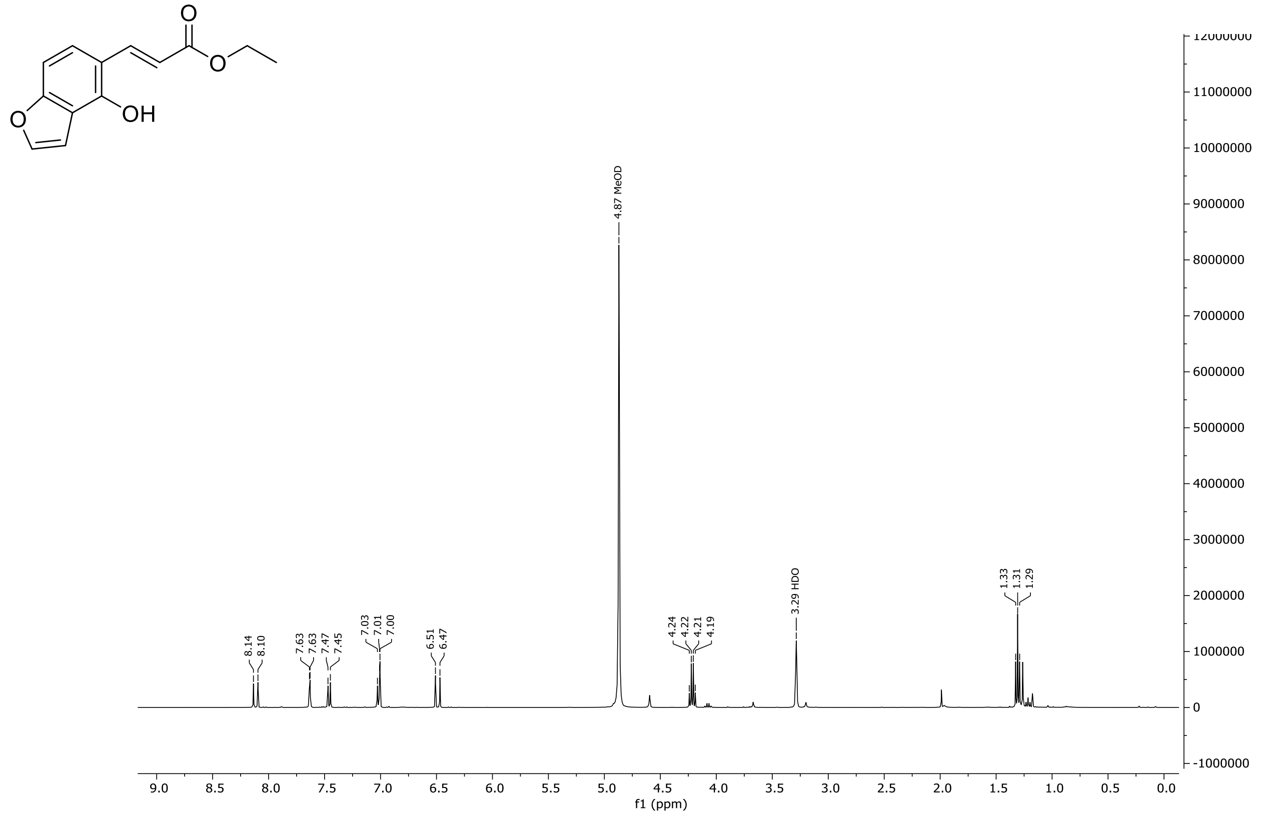

^{13}C-NMR of 27 (400 MHz, Methanol-d_4)

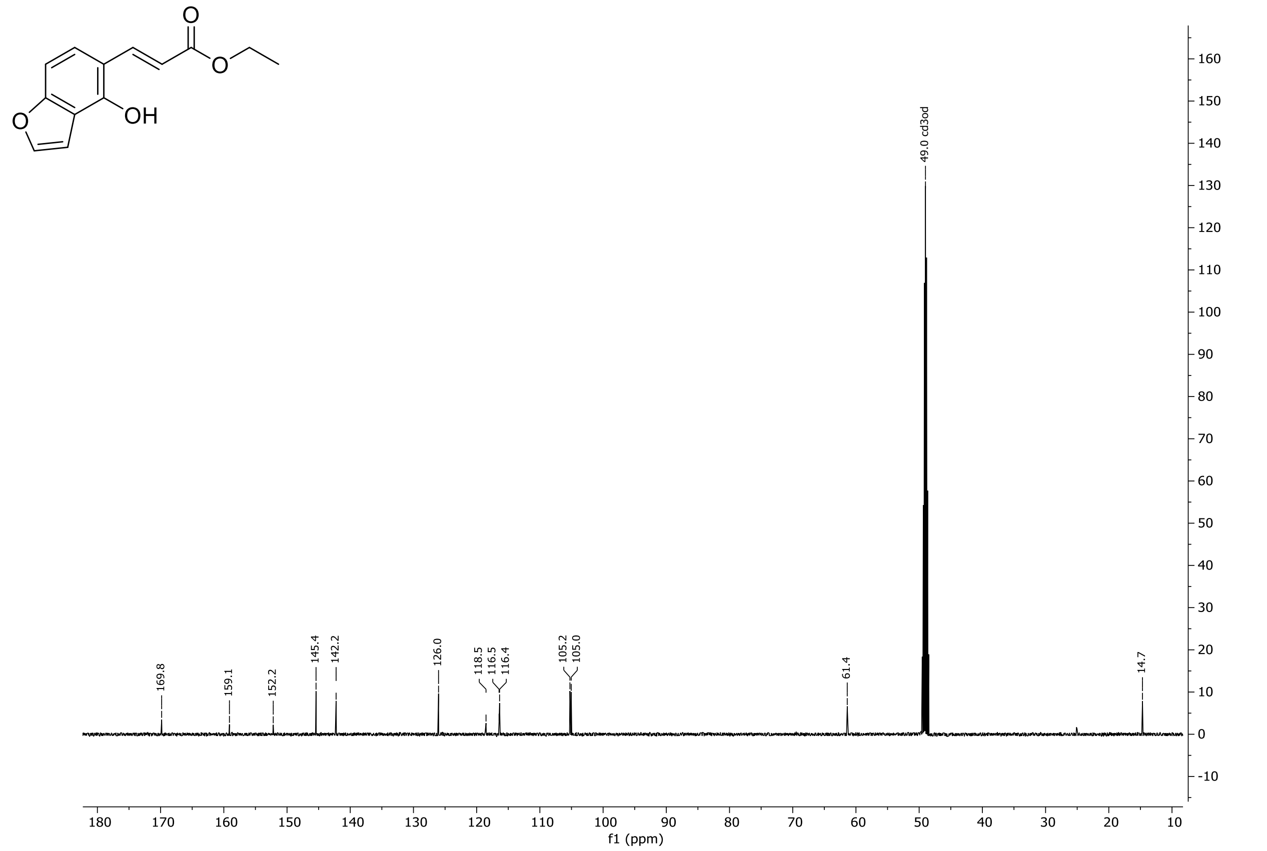

5. References

[1] J. Sileikyte, J. Devereaux, J. de Jong, M. Schiavone, K. Jones, A. Nilsen, P. Bernardi, M. Forte, M. S. Cohen, *ChemMedChem* **2019**, *14*, 1771 – 1782.

[2] T. H. McLean, J. C. Parrish, M. R. Braden, D. Marona-Lewicka, A. Gallardo-Godoy, D. E. Nichols, *J. Med. Chem.* **2006**, *49*, 5794 – 5803.

[3] Y. Tan, A. Ali, S. Tee, J., Teo, Y. Xi, M. Go, Y. Lam, *Eur. J. Med. Chem.* **2019**, *182*, 111597.

[4] M.P. Restrepo, E.G. Jaramillo, A.M. Martinez, S.R. Restrepo, *Medicinal Chemistry Research* **2018**, *27*, 2454 – 2565.

[5] S. Wiegand, H. J. Schäfer, *Tetrahedron* **1995**, *18*, 5341 – 5350.

[6] Y. Mace, E. Bony, D. Delvaux, A. Pinto, V. Mathieu, R. Kiss, O. Feron, J. Quetin-Leclercq, O. Riant, *Med. Chem. Res.* **2015**, *24*, 3143 – 3256.

[7] N. J. Lawrence, F. A. Ghani, L. A. Hepworth, J. A. Hadfield, A. T. McGohn, R. G. Pritchard, *Synthesis* **1999**, *9*, 1656 – 1660.

[8] L. Deng, A. W. Kleij, W. Yang, *Chem Eur J.* **2018**, *24*, 19156 – 19161.

[9] Z. Li, N. Liu, J. Tu, C. Ji, G. Han, C. Sheng, *ACS Infect. Dis.* **2019**, *8*, 1376 – 1384.

[10] T. Yoshikawa, M. Shindo, *Org. Lett.* **2009**, *23*, 5378 – 5381.

[11] P. Maity, S. D. Lepore, *J. Org. Chem*. **2009**, *74*, 158 – 162.

[12] R. L. Sahani, R. Liu, *Angew. Chem. Int. Ed.* **2017**, *56*, 1026 – 1030; *Angew. Chem.* **2017**, *129*, 1046 – 1050.

[13] E. Piers, T. Wong, K. A. Ellis *Can. J. Chem*. **1992**, *70*, 2058 – 2064.

[14] C. L. Peck, J. A. Calderone, W. L. Santos, *Synthesis* **2015**, *47*, 2242 – 2248.

[15] R. Volpe, R. J. Lepage, J. M. White, E. H. Krenske, B. L. Flynn, *Chem. Sci*., **2018**, *9*, 4644 – 4649.

[16] J. J. Molloy, J. B. Metternich, C. G. Daniliuc, A. J. B. Watson, R. Gilmour, *Angew. Chem. Int. Ed.* **2018**, *57*, 3168; *Angew. Chem*. **2018**, *130*, 3222 – 3226.

[17] C Feng, H. Wang, L. Xu, P. Li, *Org. Biomol. Chem.* **2015,** *13*, 7136 – 7139.

[18] S. K. Gadakh, S. Dey, A. Sudalai, *J. Org. Chem.* **2015**, *80*, 11544 – 11550.

[19] M. Kojima, K. Oisaki, M. Kanai, *Chem. Comm.* **2015**, *51*, 9718 – 9721.

[20] S. Zhang, L. Li, H. Wang, W. Liu, K. Xu, C. Zeng, *Org. Lett.* **2018**, *1*, 252 – 255.

[21] D.C. Dittmer, Q. Li, D.V. Avilov, *J. Org. Chem.* **2005**, *70*, 4682 – 4686.

[22] X. Liu, S. Zhang, C. Jiang, J. Wu, Q. Li, H. Wang, *Org. Lett.* **2015**, *21,* 5404 – 5407.

[23] A. Peyret, E. Ibarboure, A. Tron, L. Beaut, R. Rust, O. Sandre, N. D. McClenaghan, S. Lecommandoux, *Angew. Chem. Int. Ed.* **2017**, *56*, 1566 – 1570; *Angew. Chem.* **2017**, *129*, 1588 – 1592.

[24] N. Myung, S. Connelly, B. Kim, S. J. Park, I. A. Wilson, J. W. Kelly, S. Cho, *Chem. Commun.* **2010**, *49*, 9188 – 9190.

[25] C. E. Song, D. Jung, S. Y. Choung, E. J. Roh, S. Lee, *Angew. Chem. Int. Ed.* **2004**, *43*, 6183 – 6185.

[26] M. L. N. Rao, B. S. Ramakrishna, *J. Org. Chem.* **2019**, *84*, 5677 – 5683.

[27] K. Zeitler, C. A. Rose, *J. Org. Chem.* **2009**, *74*, 1759 – 1762.

[28] C. Jin, Z. Yan, B. Sun, J. Yang, *Org. Lett.* **2019**, *7*, 2064 – 2068.

[29] C. Schultze, B. Schmidt, *Beilstein J. Org. Chem.* **2018,** *14,* 2991 – 2998.
